# NUB1 reduction promotes PCNA-mediated tumor growth by disturbing the PCNA polyubiquitination/NEDDylation in hepatocellular carcinoma cells

**DOI:** 10.1038/s41419-025-07567-3

**Published:** 2025-03-31

**Authors:** Dongnian Du, Wenming Zhang, Dandan Zhang, Lingpeng Liu, Jiajuan Li, Zehao Chen, Xuzhe Yu, Miao Ye, Wei Wang, Zijing Li, Jianghua Shao

**Affiliations:** 1 Department of General Surgery, The Second Affiliated Hospital of Nanchang University, Nanchang, 330000 China; 2 Jiangxi Province Key Laboratory of Molecular Medicine, Second Affiliated Hospital of Nanchang University, Nanchang, 330000 China; 3 Liver Cancer Institute, Nanchang University, Nanchang, 330000 China; 4 Jiangxi Province Clinical Research Center of General Surgery, Second Affiliated Hospital of Nanchang University, Nanchang, 330000 China; 5 The MOE Basic Research and Innovation Center for the Targeted Therapeutics of Solid Tumors, Nanchang University, Nanchang, China

**Keywords:** Tumour-suppressor proteins, Neddylation

## Abstract

Negative regulator of ubiquitin-like protein 1 (NUB1), an inhibitor of neural precursor cells expressed developmentally downregulated 8 (NEDD8), is implicated in tumor growth. However, the expression of NUB1 in hepatocellular carcinoma (HCC) and its effects on HCC growth remain unclear. In this study, our findings revealed reduced NUB1 protein expression in HCC tissues and cells, leading to increased proliferating cell nuclear antigen (PCNA) protein stability through upregulating NEDD8 to promote HCC cell growth. Mechanistically, NUB1 reduction upregulated NEDD8 to promote PCNA NEDDylation at lysine 164 (Lys164), in turn, antagonized PCNA K48-linked polyubiquitination, thereby increasing the stability of PCNA in HCC cells. Finally, the results of the in vitro and in vivo experiments revealed that the NEDDylation inhibitor TAS4464 could inhibit PCNA NEDDylation to decrease PCNA protein expression, thereby suppressing HCC cell growth. Collectively, our results identified NUB1 as a negative regulator of HCC proliferation and confirmed that PCNA NEDDylation promotes PCNA protein stability by antagonizing PCNA polyubiquitination. This study provides a new perspective on the specific mechanism of HCC growth. It expands our understanding of the role of NEDDylation in the regulation of substrate proteins and their functions.

## Introduction

Hepatocellular carcinoma (HCC) is one of the most commonly diagnosed cancers [1]. Despite advances in the treatment of tumors over the past decades, the mortality rate of patients with HCC continues to rise, and the current 5 year survival rate is only ~18% [2, 3]. The uncontrolled proliferation of HCC cells is a key factor that leads to tumor spread and poor prognosis in patients with HCC [4]. Therefore, there is an urgent need to elucidate the regulatory mechanisms of HCC proliferation further and discover more effective intervention targets to provide effective strategies for the treatment of HCC.

Negative regulator of ubiquitin-like protein 1 (NUB1), also known as the neural precursor cell expressed developmentally downregulated 8 (NEDD8) ultimate buster-1, consists of a ubiquitin-like (UBL) domain at its N-terminus and two ubiquitin-associated (UBA) domains at its C-terminus [5]. NUB1 can directly bind to the proteasome subunit S5a at its C-terminus, thereby facilitating the proteolytic degradation of proteins that directly bind to NUB1, such as NEDD8 [6–8]. Previous studies have indicated that dysregulated NUB1 expression may contribute to rheumatoid arthritis and a range of neurodegenerative diseases [8–11]. Recently, the role of NUB1 in tumor growth has attracted considerable attention. For example, NUB1 knockdown suppressed the growth of breast cancer cells in vitro by inducing cell cycle arrest [12]. In addition, studies have revealed that NUB1 knockdown promotes tumor growth in renal cell carcinoma, osteosarcoma, and gastric cancer by upregulating NEDD8 and its conjugation system [13–15]. However, the expression of NUB1 in HCC and its effects on HCC growth have not yet been reported.

Proliferating cell nuclear antigen (PCNA), a master regulator of cell proliferation, is highly expressed in various tumors, including HCC tissues, and is closely associated with poor patient prognosis [16–20]. As a moving scaffold, PCNA functions primarily through the protein itself. In the context of rapid cell proliferation or stimulation by hypoxia, the PCNA protein is significantly upregulated, and PCNA promotes various cellular processes through interactions with diverse proteins [21–23]. Therefore, an in-depth investigation of the factors regulating PCNA protein expression is of great significance for a comprehensive understanding of cellular biological processes. The UPS degrades PCNA, and various factors regulate its stability [24–26]. Studies have demonstrated that crosstalk between other PTMs, such as phosphorylation, methylation, and acetylation, and the ubiquitination of PCNA plays important roles in regulating its stability and function in various cells [27]. As an important type of PTM, NEDDylation is involved in regulating the stability and function of several substrate proteins under the successive action of E1, E2, and E3 enzymes [28]. A recent study indicated that NEDDylation also plays an important role in regulating the function of PCNA, and increased PCNA NEDDylation antagonizes PCNA monoubiquitylation, thereby impairing the DNA damage tolerance of cells [29]. However, whether PCNA NEDDylation affects PCNA stability and its underlying mechanisms remains unclear. In this study, we aimed to answer these questions.

## Materials and methods

### Human tissue specimens

Human HCC specimens were collected from 60 patients who underwent HCC resection at the Second Affiliated Hospital of Nanchang University between January 2021 and June 2022. Informed consent was obtained from each patient, and the study protocol was approved by the Ethics Committee of the Second Affiliated Hospital of Nanchang University.

### Cell culture and reagents

A human-derived liver cell line (HL-7702) and human hepatocellular carcinoma cell lines (HepG2, SMMC7721, Huh-7, Hep3B, HCCLM3 and MHCC97H) were purchased from the Shanghai Institute of Cell Biology, China. The cells were cultured in DMEM (Solarbio, Beijing, China) supplemented with 10% fetal bovine serum (Bovogen, Melbourne, Australia). All cell lines were free of mycoplasma and authenticated using STR. Primary antibodies used for western blotting (WB), immunoprecipitation (IP), and immunofluorescence (IF) are as follows: antibodies against NUB1 (14343-1-AP); Tubulin (66031-1-Ig); 6×his, a His tag polyclonal antibody (10001-0-AP); and IgG (30000-0-AP) were purchased from Proteintech (Wuhan, China). Antibodies against NEDD8 [Y297] (ab81264) and PCNA[PC10] (ab29) were purchased from Abcam (Cambridge, UK). Antibodies against Flag (Cat# F3165) and HA (Cat# H6908) were purchased from Sigma-Aldrich (Shanghai, China). HRP-labeled anti-mouse (Cat# sc-2005) and anti-rabbit (Cat# sc-2004) secondary antibodies were purchased from Santa Cruz Biotechnology (Santa Cruz, CA, USA). A/G magnetic beads (Cat# B23201) were purchased from Bimake (Houston, TX, USA). MG132, CHX, and TAS4464 were purchased from MedChemExpress (NJ, USA).

### Co-immunoprecipitation (Co-IP) and immunoblotting assay

Lipofectamine 3000 reagents (Invitrogen) were used for the transient transfection of plasmids into cells, if needed. Cells were harvested, and total cell lysates were obtained using Western and IP lysates (Beyotime, Shanghai, China) supplemented with phosphatase and protease inhibitors for further IP and immunoblotting assays. The primary antibody and Protein A/G PLUS-Agarose (40 μl, Santa Cruz Biotechnology, sc-2003) were added and incubated for 12 h at 4 °C, followed by three washes with the same lysis buffer. Immunocomplexes were analyzed by performing an immunoblotting assay. Briefly, samples were separated via 10% SDS-PAGE and subsequently transferred to PVDF membranes. After blocking with 5% skim milk, blots were incubated with the primary antibody overnight at 4 °C and reacted with the HRP-conjugated mouse or rabbit secondary antibody at room temperature for 1 h. AI6000 software was used to analyze the blots. Tubulin was used as loading controls. The experiment was repeated at least three times.

### Tumorigenicity assay and bioluminescence imaging

The HCCLM3 and MHCC97H cells used for injection were stably transduced with the firefly luciferase gene, enabling regular in vivo monitoring of tumor growth via bioluminescence imaging. For the in vivo tumorigenicity assay, 6–8 week-old male athymic nude mice (BALB/c-nu/nu) were purchased from GemPharmatech (Nanjing, China), and 1 × 10^6^ cells were injected subcutaneously into the ventral side of nude mice. Then, for in vivo signal detection, animals were injected intraperitoneally with luciferin and anesthetized via isoflurane inhalation. Bioluminescence imaging was performed using an IVIS spectroscopy system (PerkinElmer, USA). At the end of the experiment, the mice were euthanized, and the tumor weights and volumes were recorded. The tumor specimens were stored in liquid nitrogen or fixed in formalin for further analysis. As the in vivo experiments entailed the detection of protein expression or the effect of the drug on specific markers, the number of nude mice in each experimental group was set at 10. Nude mice were randomly assigned to specific experimental groups.All animal experiments were approved by the animal research committee in the Laboratory Animal Science Center of Nanchang University (NCULAE-20221031108).

### Statistical analysis

Statistical analyses were performed using GraphPad Prism (version 8.0). All results are expressed as mean ± standard deviation (SD). For comparisons between two groups, Student’s *t*-tests were used, while Tukey’s post hoc test and one-way ANOVA were used for multiple comparisons. The threshold of statistical significance was *p* < 0.05. All experiments were independently repeated at least three times.

Other materials and methods are provided in the supplementary materials and methods.

## Results

### The protein expressions of NUB1 and PCNA in HCC tissues and HCC cells are negatively correlated

**Fig. 1:**
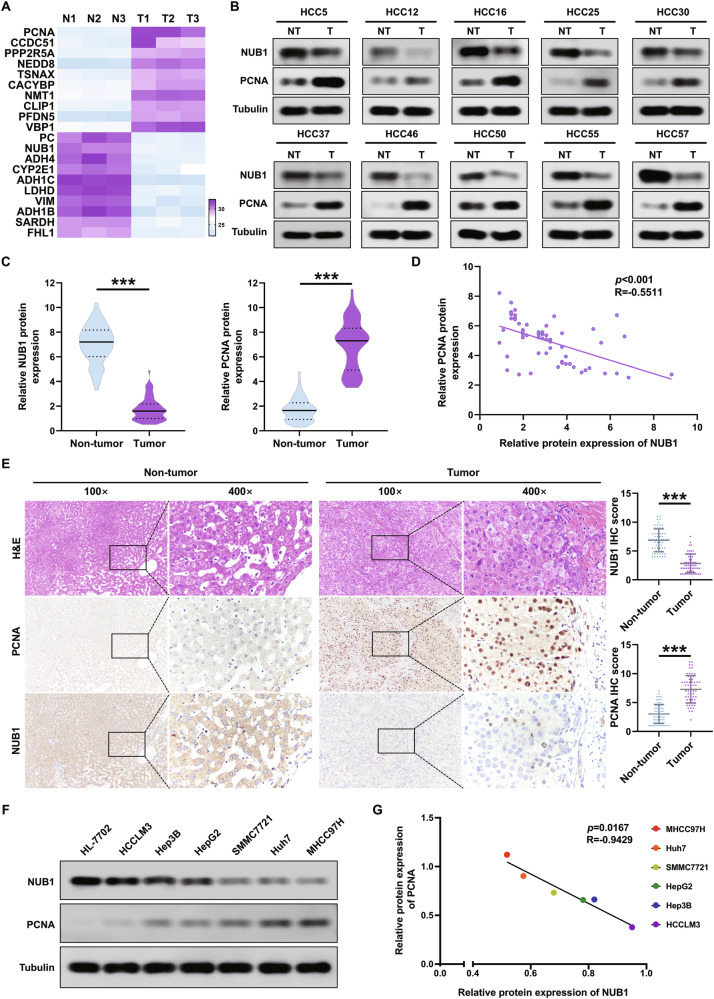
The protein expressions of NUB1 and PCNA in HCC tissues and HCC cells are negatively correlated. **A** HCC tissues and adjacent nontumor tissue samples were subjected to the quantitative proteomic analysis (*n* = 3). **B**, **C** Determination(**B**) and quantification(**C**) of NUB1 and PCNA protein levels in 60 paired HCC tissues and adjacent nontumor tissues by immunoblotting. **D** Scatter plots showed a negative correlation between protein expression of NUB1 and PCNA in HCC tissues. **E** Representative IHC staining and quantitative analysis of NUB1 and PCNA protein expression in 60 pairs of HCC tissues and adjacent nontumor tissues. Scale bars = 200 µm and 50 µm. **F** Western blot assay detects NUB1 and PCNA protein expression in normal human liver HL7702 cells and six tested HCC cells (HCCLM3, Hep3B, HepG2, SMMC7721, Huh7, and MHCC97H). **G** Scatter plots showed a negative correlation between protein expression of NUB1 and PCNA in HCC cells. Data are represented as the mean ± SD. *p < 0.05; **p < 0.01; ***p < 0.001.

To investigate the relationship between NUB1 expression and PCNA expression, we performed the quantitative proteomic analysis of three pairs of HCC and corresponding adjacent tissues. The results showed that compared to the corresponding adjacent tissues, the expression of NUB1 was decreased in HCC tissues; in contrast, the expression of PCNA protein was increased in HCC tissues (Fig. 1A). Next, the results of NUB1 and PCNA protein expression in 60 fresh HCC tissue samples using western blotting also showed that NUB1 protein expression was decreased in HCC tissues, whereas PCNA protein expression was increased in HCC tissues; correlation analysis showed a negative correlation between them (Fig. 1B–D). Furthermore, immunohistochemical (IHC) staining results confirmed the decreased expression of NUB1 protein and increased expression of PCNA protein in HCC tissues compared to adjacent tissues, which was consistent with the above results (Fig. 1E). Moreover, the results of western blot showed that the protein expression levels of NUB1 were lower in six tested HCC cells (i.e., HCCLM3, Hep3B, HepG2, SMMC7721, Huh7, and MHCC97H) than in normal hepatocytes (HL-7702), whereas the opposite was true for PCNA, and correlation analysis also showed a negative correlation between them (Fig. 1F, G). These observations suggest that NUB1 and PCNA exhibit low and high expression levels in HCC tissues and HCC cells, respectively, and that both are negatively correlated.

### Overexpression of NUB1 suppresses HCC cell growth by reducing PCNA expression in vitro and in vivo

**Fig. 2:**
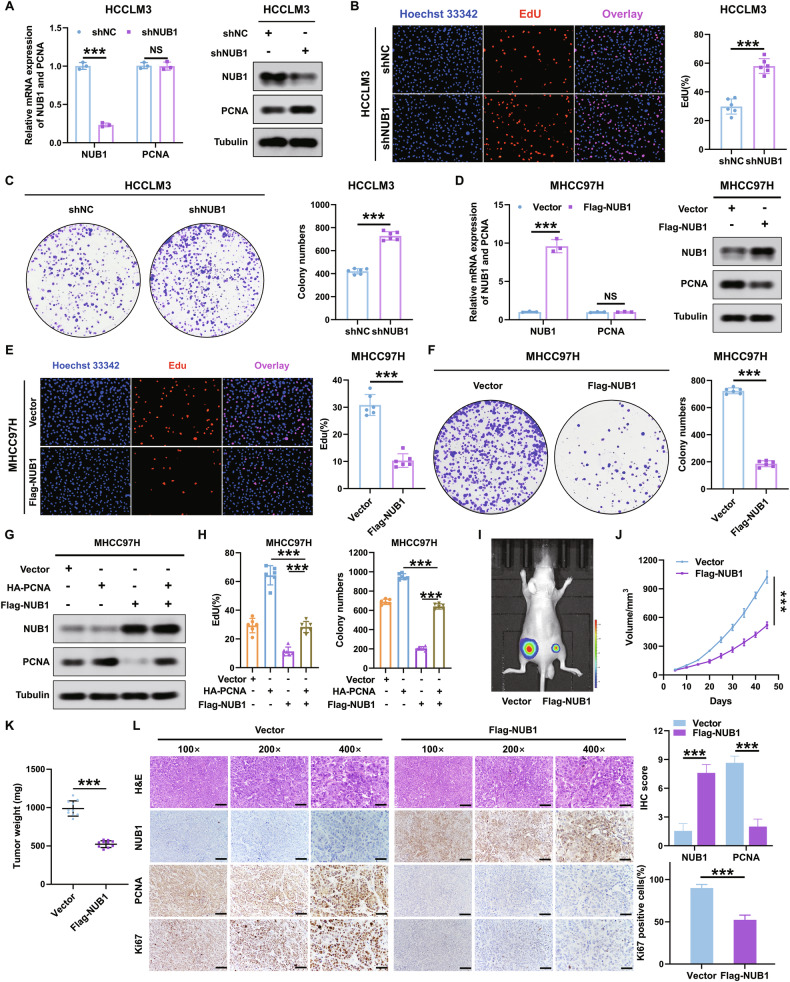
NUB1 overexpression reduces PCNA expression to suppress HCC cell growth in vitro and in vivo. **A** HCCLM3 cells were transfected with shRNA against NUB1 and the cell lysates were subjected to qRT-PCR and immunoblotting (*n* = 3). The mRNA levels of NUB1 and PCNA were normalized to GAPDH. **B**, **C** EdU (**B**) and colon formation assays (**C**) detected the proliferation capacities of HCCLM3 cells with the indicated treatments (*n* = 6). **D** MHCC97H cells were transfected with NUB1 overexpression lentivirus and the cell lysates were subjected to qRT-PCR and immunoblotting (*n* = 3). The mRNA levels of NUB1 and PCNA were normalized to GAPDH. **E**, **F** EdU (**E**) and colon formation assays (**F**) detected the proliferation capacities of MHCC97H cells with the indicated treatments (*n* = 6). **G** Determination of the protein levels of NUB1 and PCNA by immunoblotting with the indicated treatments in MHCC97H cells. **H** EdU and colon formation assays detected the proliferation capacities of MHCC97H cells with the indicated treatments (*n* = 6). **I** Representative bioluminescent images in nude mice subcutaneously injected MHCC97H cells with lentivirus encoding NUB1 or control (*n* = 10). **J**, **K** Tumor volumes (**J**) and weights (**K**) in nude mice intravenously subcutaneously injected MHCC97H cells with lentivirus encoding NUB1 or control (*n* = 10). **L** Representative immunohistochemical staining photograph of NUB1, PCNA, and Ki67 in the same of the subcutaneous tumors in the above groups. Scale bars = 200 µm, 100 µm and 50 µm. Data represent the mean ± SD. *p < 0.05; **p < 0.01; ***p < 0.001.

Next, we wanted to investigate whether NUB1 could regulate PCNA expression and thus affect the proliferation of HCC cells. To this end, based on the results of Fig. 1F, we selected NUB1 high-expressing cells (HCCLM3) and NUB1 low-expressing cells (MHCC97H) for subsequent experiments and then constructed HCCLM3 and MHCC97H cell lines that stably overexpressed or knocked down NUB1. The results of qRT-PCR and western blotting indicated that the mRNA expression level of PCNA was unchanged, but its protein expression increased in HCC cells of the NUB1 knockdown group compared to that in the control group (Fig. 2A and Supplementary Fig. 1A). Moreover, we observed that the cell proliferation ability was significantly increased in the NUB1 knockdown group by *5-Ethynyl-2’-deoxyuridine* (EdU) and colony formation assays (Fig. 2B, C and Supplementary Fig. 1B, C). In contrast, overexpression of NUB1 decreased PCNA protein expression and the proliferation ability of HCC cells (Fig. 2D–F and Supplementary Fig. 1D–F). Rescue experiments revealed that NUB1 overexpression suppressed MHCC97H cell proliferation by reducing PCNA protein expression, whereas PCNA overexpression attenuated the decrease in PCNA expression caused by NUB1 overexpression in MHCC97H cells (Fig. 2G, H). Rescue experiments revealed that NUB1 knockdown upregulated PCNA protein expression, whereas PCNA knockdown attenuated the increase in PCNA expression caused by NUB1 knockdown in HCCLM3 cells (Supplementary Fig. 1G). EdU and colony formation assays also showed that NUB1 knockdown significantly promoted the proliferation of HCCLM3 cells, whereas PCNA knockdown reversed this NUB1 knockdown-induced increase in proliferation (Supplementary Fig. 1H). These results revealed that NUB1 overexpression suppresses the proliferation of HCC cells in vitro by reducing PCNA protein expression.

Furthermore, to confirm that upregulated NUB1 reduced PCNA protein expression to suppress HCC cell growth in vivo, we established a subcutaneous tumor model in BALB/c nude mice using Flag-NUB1, Flag-vector HCCLM3, and MHCC97H cells. After 45 days, the in vivo imaging system (IVIS) results showed a significant reduction in fluorescence intensity in the Flag-NUB1 group compared to that in the Flag-vector group (Fig. 2I and Supplementary Fig. 1I). Tumor volume and weight were dramatically decreased in the Flag-NUB1 group compared to those in the control group (Fig. 2J, K and Supplementary Fig. 1J, K). IHC staining further showed that PCNA expression and the number of Ki-67-positive cells were reduced in the subcutaneous tumor tissues of the Flag-NUB1 group (Fig. 2L and Supplementary Fig. 1L). Overall, our results indicate that NUB1 overexpression reduces PCNA protein expression to suppress HCC cell growth in vitro and in vivo.

### NUB1 reduction inhibits PCNA degradation by the UPS to upregulate PCNA expression

**Fig. 3:**
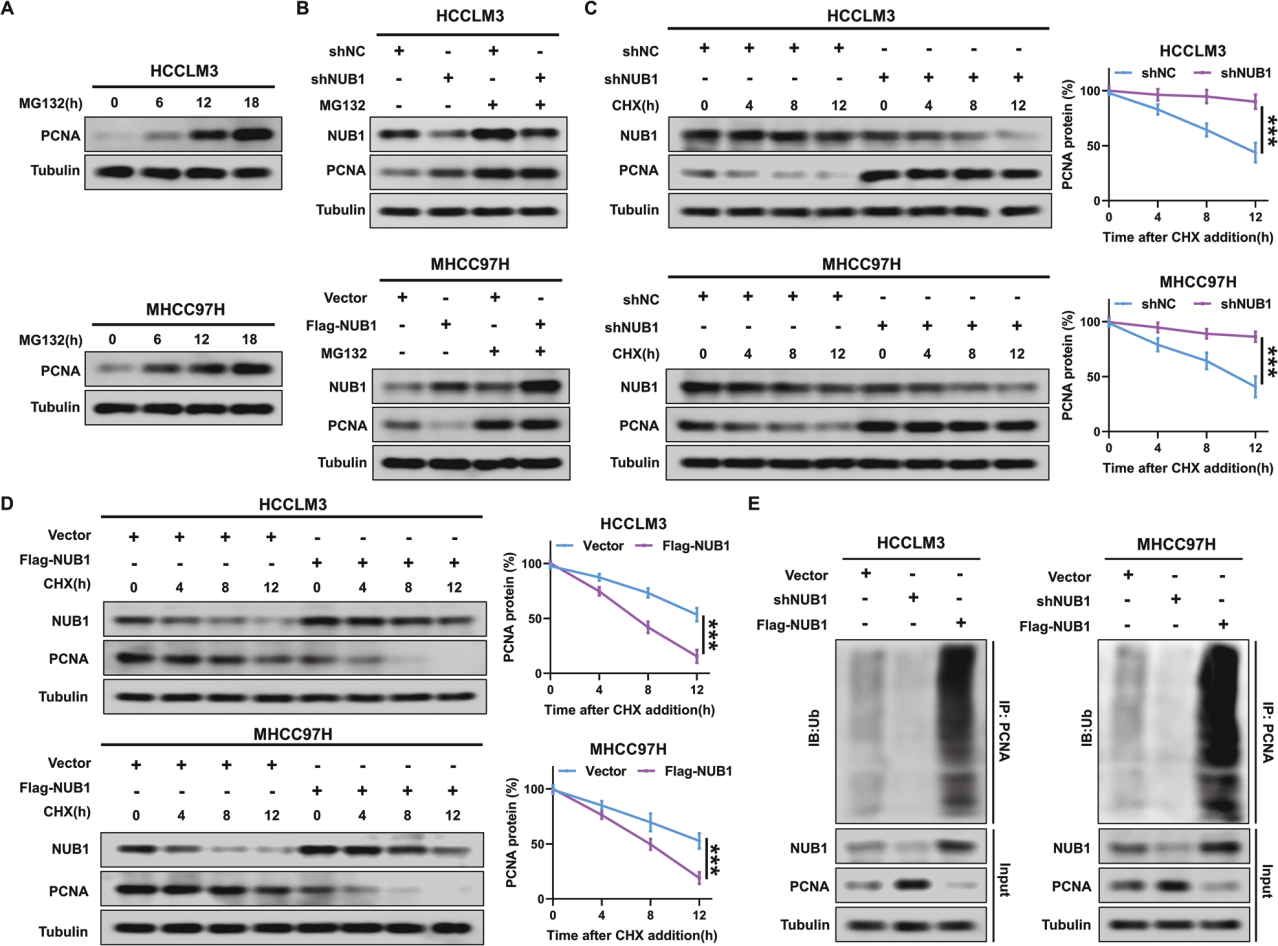
NUB1 reduction inhibits PCNA degradation by the UPS to upregulate PCNA expression. **A** HCCLM3 and MHCC97H cells were treated with MG132 (15 nM) for the indicated times, and the cell lysates were subjected to immunoblotting. **B** HCCLM3 and MHCC97H cells were transfected with shNC and shNUB1 for 48 h and then treated with MG132 (15 nM) for 12 h, and the cell lysates were subjected to immunoblotting. **C** and (**D**). HCCLM3 and MHCC97H cells were transfected with the indicated plasmid. The cells were subjected to 20 μg /ml CHX exposure at the indicated time, and the degradation of PCNA was detected by immunoblotting. **E** HCCLM3 and MHCC97H cells were transfected with blank vector, shNUB1 or Flag-NUB1, and then the cell lysates were subjected to Co-IP and immunoblotting. Data represent the mean ± SD. *p < 0.05; **p < 0.01; ***p < 0.001.

The objective of subsequent investigations was to ascertain the mechanism by which NUB1 regulates PCNA protein expression. Previous studies have demonstrated that UPS can degrade PCNA [24, 25]. Therefore, it was postulated that NUB1 may regulate PCNA protein expression by affecting its degradation by the UPS. Our findings first confirmed that proteasomes in HCCLM3 and MHCC97H cells can degrade PCNA (Fig. 3A). Next, our results found that NUB1 was involved in regulating the proteasomal degradation of PCNA in HCC cells. We altered NUB1 expression in HCC cells with or without treatment with the proteasome inhibitor MG132 (15 μM, 12 h), and western blotting showed that MG132 treatment abolished the change in PCNA expression mediated by the alteration of NUB1 expression (Fig. 3B).

Furthermore, the cycloheximide (CHX) chase experiments (20 μg /ml, 4, 8 and 12 h) demonstrated that NUB1 overexpression shortened the half-life of PCNA, and NUB1 reduction prolonged its half-life by the degradation dynamics assay in HCC cells (Fig. 3C, D). Finally, our results confirmed that NUB1 affects PCNA protein expression by regulating ubiquitin-mediated proteasomal degradation of PCNA. We transfected with shNUB1 or Flag-NUB1 in HCCLM3 and MHCC97H cells. After cell lysis, the lysates were mixed with a PCNA antibody for immunoprecipitation, which showed that PCNA polyubiquitination was markedly diminished in shNUB1 HCC cells; in contrast, it was increased in Flag-NUB1 HCC cells (Fig. 3E). Collectively, these findings indicate that NUB1 reduction inhibits the degradation of PCNA by the UPS, thereby increasing its expression in HCC cells.

### NUB1 reduction promotes PCNA protein expression by increasing NEDD8 in HCC cells

**Fig. 4:**
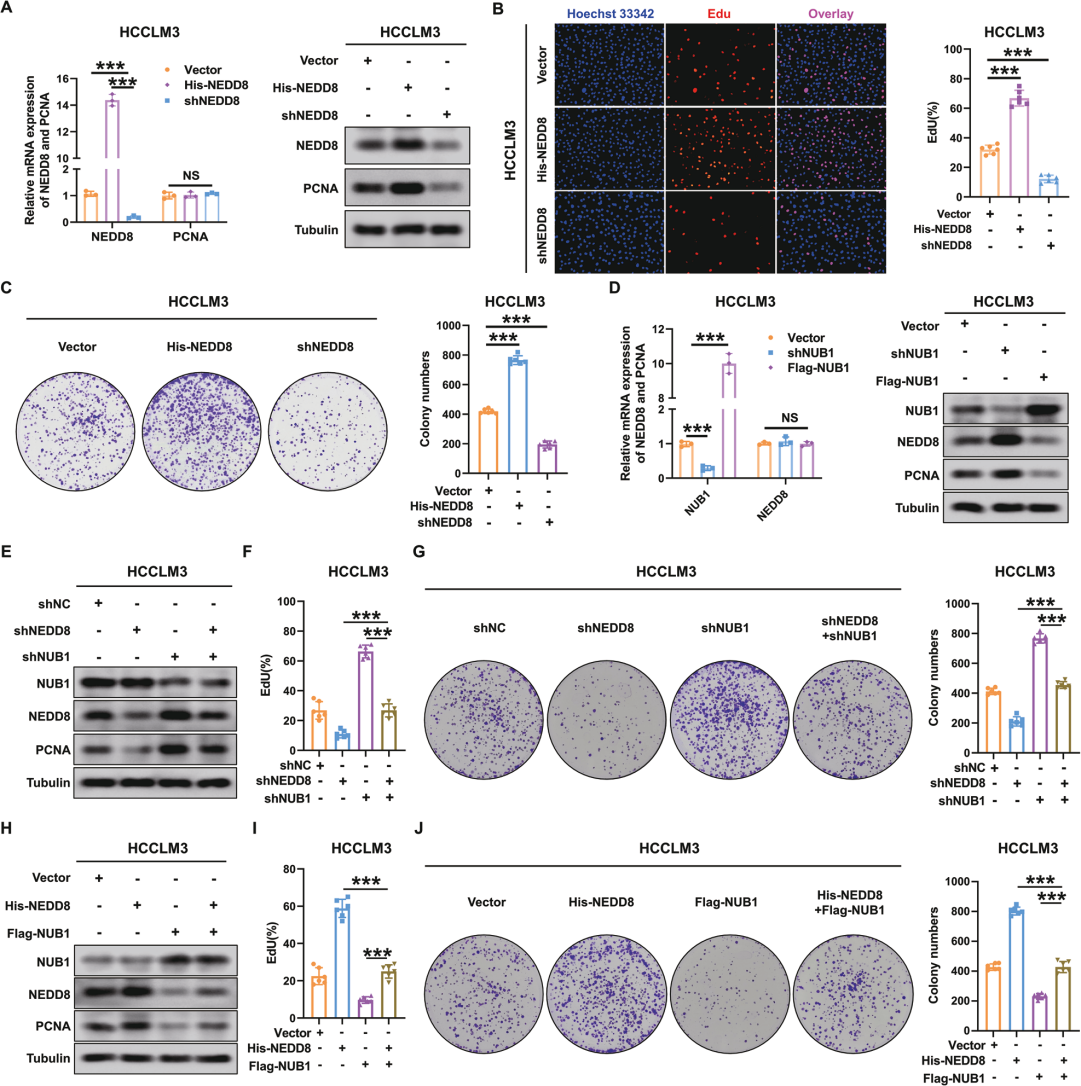
NUB1 reduction promotes PCNA protein expression by increasing NEDD8 in HCCLM3 cells. **A** qRT-PCR and western blot were used to analyze the mRNA and protein levels of NEDD8 and PCNA in HCCLM3 cells with the indicated treatments (*n* = 3). The mRNA levels of NEDD8 and PCNA were normalized to GAPDH. **B**, **C** EdU (**B**) and colon formation assays (**C**) detected the proliferation capacities of HCCLM3 cells with the indicated treatments (*n* = 6). **D** qRT-PCR and western blot were used to analyze the mRNA and protein levels of NUB1 and NEDD8 in HCCLM3 cells with the indicated treatments (*n* = 3). The mRNA levels of NUB1 and NEDD8 were normalized to GAPDH. **E** HCCLM3 cells transfected with shNEDD8 or control were transfected with or without shNUB1 and the cell lysates were subjected to immunoblotting. **F** and **G** EdU (**F**) and colon formation assays (**G**) analyses for the above groups (*n* = 6). **H** HCCLM3 cells transfected with His-NEDD8 or control were transfected with or without Flag-NUB1 and the cell lysates were subjected to immunoblotting. **I**, **J** EdU (**I**) and colon formation assays (**J**) analyses for the above groups (*n* = 6). Data represent the mean ± SD. *p < 0.05; **p < 0.01; ***p < 0.001.

NUB1 interacts with proteins to promote proteasomal degradation [7, 8]. However, co-immunoprecipitation (co-IP) and GST pull-down experiments showed no direct interactions between NUB1 and PCNA in HCCLM3 and MHCC97H cells (Supplementary Fig. 2A, B). This suggests the presence of intermediate mediators involved in NUB1-induced proteasomal degradation of PCNA in HCC cells. Previous studies have confirmed that NUB1 can regulate the protein expression of NEDD8 and that PCNA is a substrate of NEDDylation [14, 29]. Moreover, Fig. 1A revealed that the expression of NEDD8 is elevated in HCC tissues compared to corresponding adjacent tissues. Therefore, we hypothesized that NUB1 regulates the ubiquitin-proteasomal degradation of PCNA by regulating NEDD8. Our results revealed that NEDD8 regulates PCNA protein expression and subsequently affects the proliferation of HCC cells. We constructed HCCLM3 and MHCC97H cell lines with stably high NEDD8 expression (His-NEDD8) and low NEDD8 expression (shNEDD8). The results of qRT-PCR and western blotting indicated that the mRNA level of PCNA remained unchanged, but its protein level decreased in the shNEDD8 group and increased in the His-NEDD8 group (Fig. 4A and Supplementary Fig. 2C). EdU and colony formation assays showed that NEDD8 knockdown inhibited the proliferation of HCC cells, whereas NEDD8 overexpression promoted their proliferation of HCC cells (Fig. 4B, C and Supplementary Fig. 2D, E).

Next, our results confirm that NUB1 regulates the ubiquitin-proteasomal degradation of PCNA by affecting NEDD8 expression in HCC cells. The results of qRT-PCR and western blotting showed that, compared to the control group, the mRNA level of NEDD8 was unchanged, but its protein level was increased in the HCC cells of the shNUB1 group and decreased in the HCC cells of the Flag-NUB1 group (Fig. 4D and Supplementary Fig. 2F). Furthermore, rescue experiments showed that NUB1 knockdown increased the protein expression of NEDD8 and PCNA and that NEDD8 knockdown alleviated the increase in NEDD8 and PCNA expression caused by NUB1 downregulation in HCC cells (Fig. 4E and Supplementary Fig. 2G). EdU and colony formation assays consistently showed that NEDD8 knockdown alleviated the enhanced proliferation induced by NUB1 downregulation in HCC cells (Fig. 4F, G and Supplementary Fig. 2H, I). In contrast, NEDD8 overexpression attenuated the decrease in NEDD8 and PCNA expression and reduced the proliferation of HCC cells caused by increased NUB1 expression (Fig. 4H–J and Supplementary Fig. 2J-L). Overall, these results confirmed that NUB1 affects the protein expression of PCNA by regulating NEDD8 expression in HCC cells.

### NEDD8-mediated PCNA NEDDylation antagonizes PCNA K48-linked polyubiquitination to promote PCNA expression in HCC cells

**Fig. 5:**
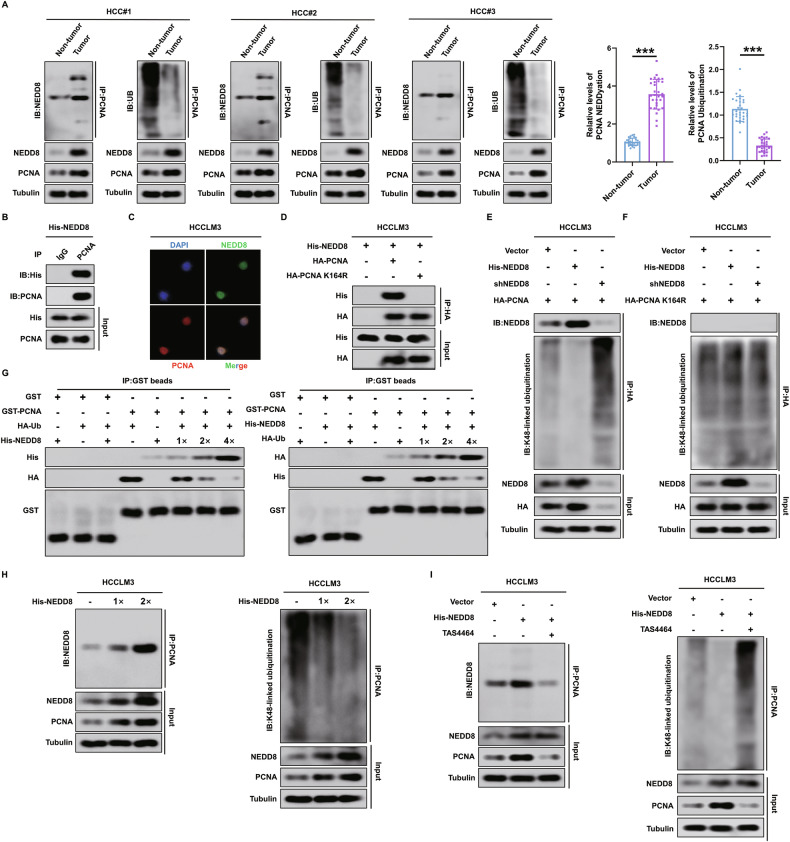
NEDD8-mediated PCNA NEDDylation antagonizes PCNA K48-linked polyubiquitination to promote PCNA expression in HCCLM3 cells. **A** Determination of the levels of PCNA NEDDylation and polyubiquitination in 30 paired HCC tissues and adjacent nontumor tissues by Co-IP and immunoblotting. **B** Co-IP between endogenous NEDD8 and PCNA in HCCLM3 cells. **C** Representative confocal immunofluorescence images of NEDD8 (red) colocalized with PCNA (green) in HCCLM3 cells. **D** HA-PCNA and HA-PCNA K164R plasmids were transfected into HCCLM3 cells stably transfected with His-NEDD8, respectively, and Co-IP detected the binding of the exogenous tag HA to His. **E**, **F** HCCLM3 cells were transfected with blank vector, His-NEDD8 or shNEDD8, followed by transfecting with HA-PCNA (**E**) or HA-PCNA K164R (**F**), and the cell lysates were immunoprecipitated with anti- HA antibody, followed by immunoblotting. **G** Binding of PCNA during the course of the competition was analyzed by GST pulldown experiment. **H** HCCLM3 cells transfected with increasing dose gradients of His-NEDD8, and the cell lysates were immunoprecipitated with anti-PCNA antibody, followed by immunoblotting. **I** HCCLM3 cells transfected with blank vector or His-NEDD8, and HCCLM3 His-NEDD8 cells treated with 100 nM TAS4464 for 12 h. Then, the cell lysates were immunoprecipitated with anti-PCNA antibody, followed by immunoblotting.

We explored the specific mechanisms by which NEDD8 affects PCNA protein expression in HCC cells. We first determined the relationship between PCNA NEDDylation and PCNA polyubiquitination in 30 pairs of human HCC tissue samples and their corresponding adjacent tissues. The results showed that significant accumulation of PCNA NEDDylation and significantly decreased polyubiquitination levels were observed in HCC tissues compared with corresponding adjacent tissues and the protein expression of NEDD8 and PCNA was also significantly upregulated in HCC tissues (Fig. 5A). In addition, it has been demonstrated that Ub can bind to the lys164 site of PCNA, and NEDD8 can also bind to the lys164 site of PCNA [29, 30]. Therefore, we hypothesized that increased NEDD8 can compete with Ub for binding PCNA at the lys164 site, which results in increased PCNA NEDDylation to antagonize PCNA K48-linked polyubiquitination, in turn reducing the proteasomal degradation of PCNA and thereby upregulating PCNA protein expression. Our results are the first to show that NEDD8 binds to PCNA at the lys164 site and that PCNA lys164 is essential for NEDD8-mediated PCNA NEDDylation to antagonize PCNA K48-linked polyubiquitination in HCC cells. The results of Co-IP and confocal immunofluorescence assays showed that NEDD8 can interact with PCNA in HCCLM3 and MHCC97H (Fig. 5B, C and Supplementary Fig. 3A, B). Then, a wild-type plasmid of PCNA(HA-PCNA) and a mutant plasmid of PCNA at the lys164 site (HA-PCNA K164R) were transfected into HCC cells stably expressing His-NEDD8, respectively. The results of Co-IP showed that NEDD8 can bind to PCNA instead of PCNA K164R (Fig. 5D and Supplementary Fig. 3C). Moreover, we altered NEDD8 expression after the transfection of the PCNA wild-type plasmid (HA-PCNA) and the PCNA lys164 site mutant plasmid (HA-PCNA K164R) into HCCLM3 and MHCC97H cells. Co-IP and western blot experiments showed that in HCC cells transfected with HA-PCNA plasmids, NEDD8 overexpression increased PCNA NEDDylation and decreased PCNA K48-linked polyubiquitination accompanied by an increase in PCNA protein expression compared with the control group and NEDD8 knockdown yielded the opposite results (Fig. 5E and Supplementary Fig. 3D). However, in HCC cells transfected with HA-PCNA K164R plasmid, HA-PCNA K164R could not bind to NEDD8 in controls, NEDD8 overexpression and NEDD8 knockdown groups, and the level of HA-PCNA K164R K48-linked polyubiquitination and PCNA protein levels were not altered between the above groups (Fig. 5F and Supplementary Fig. 3E).

Next, we confirmed that NEDD8 could compete with Ub for binding to PCNA, and NEDD8 overexpression increased PCNA binding to NEDD8 to promote PCNA NEDDylation, in turn decreasing the binding of Ub to PCNA to decrease K48-linked polyubiquitination of PCNA. The conclusion consists of the following results. First, our data confirmed that NEDD8 can compete with Ub for binding PCNA. The results of the GST-pulldown experiment showed that the addition of increasing amounts of His-NEDD8 resulted in a gradual increase in the levels of the His-NEDD8-PCNA complex, and a gradual decrease in the levels of the Ub-PCNA complex, conversely, with the gradual increase in the UB, the Ub-PCNA complex gradually increased, and the NEDD8-PCNA complex gradually decreased (Fig. 5G). Secondly, our results showed that a gradual increase in NEDD8 leads to a gradual increase in PCNA NEDDylation and a gradual decrease in PCNA K48-linked polyubiquitination (Fig. 5H and Supplementary Fig. 3F). Finally, rescue experiments revealed that TAS4464 treatment (100 nM, 12 h), one of the most potent and selective small molecule NEDD8 activating enzyme inhibitors [31], significantly inhibited augmented PCNA NEDDylation, decreased PCNA polyubiquitination, and increased PCNA protein caused by NEDD8 overexpression in HCC cells (Fig. 5I and Supplementary Fig. 3G). Overall, our data confirmed that NEDD8 overexpression can promote PCNA NEDDylation at the lys164 site to antagonize PCNA K48-linked polyubiquitination and subsequently upregulate PCNA protein expression in HCC cells.

### NUB1 reduction disturbs the PCNA NEDDylation and K48-linked polyubiquitination to increase PCNA by NEDD8 in HCC cells

**Fig. 6:**
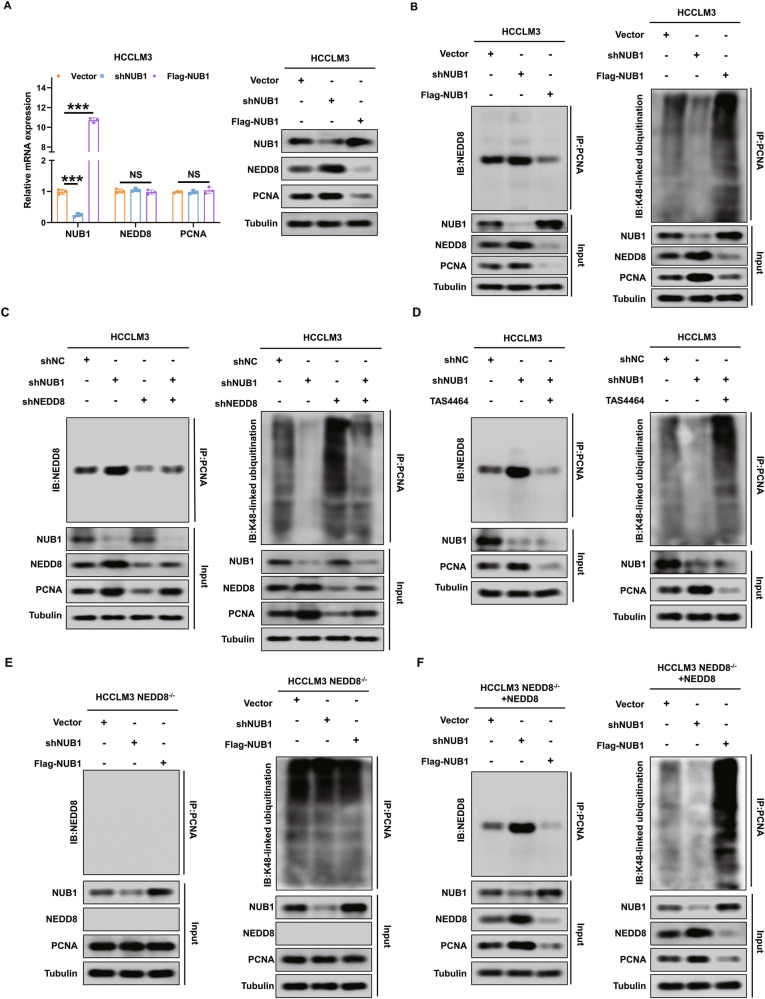
NUB1 reduction disturbs the PCNA NEDDylation/K48-linked polyubiquitination to increase PCNA by upregulating NEDD8 in HCCLM3 cells. **A** qRT-PCR and western blot were used to analyze the mRNA and protein levels of NUB1, NEDD8 and PCNA in HCCLM3 cells with the indicated treatments(*n* = 3). The mRNA levels of NUB1, NEDD8 and PCNA were normalized to GAPDH. **B** HCCLM3 cells were transfected with blank vector, shNUB1 and Flag-NUB1, respectively, and then the cell lysates were immunoprecipitated with anti-PCNA antibody, followed by immunoblotting. **C** HCCLM3 cells transfected indicated plasmids and the cell lysates were immunoprecipitated with anti-PCNA antibody, followed by immunoblotting. **D** HCCLM3 cells transfected with shNC or shNUB1, and HCCLM3 shNUB1 cells treated with TAS4464 (100 nM) for 12 h. Then the cell lysates were subjected to were immunoprecipitated with anti-PCNA antibody, followed by immunoblotting. **E** HCCLM3 NEDD8^-/-^ cells were transfected with blank vector, shNUB1 and Flag-NUB1, respectively, and then the cell lysates were immunoprecipitated with anti-PCNA antibody, followed by immunoblotting. **F** Restoration of NEDD8 expression in HCCLM3 NEDD8^-/-^ cells and transfected with blank vector, shNUB1 and Flag-NUB1, respectively, and the cell lysates were immunoprecipitated with anti-PCNA antibody, followed by immunoblotting.

Based on the above results, we speculated that NUB1 regulates PCNA expression in that NUB1 reduction upregulated NEDD8 protein to increase PCNA NEDDylation, which in turn antagonizes PCNA K48-linked polyubiquitination, resulting in decreased PCNA in HCC cells. Our data confirmed that NUB1 reduction increases NEDD8-mediated PCNA NEDDylation to inhibit PCNA K48-linked polyubiquitination, thus promoting PCNA protein expression in HCCLM3 and MHCC97H cells (Fig. 6A and Supplementary Fig. 4A). Co-IP and western blot assays showed that compared with the control group, NUB1 knockdown promoted PCNA NEDDylation and inhibited PCNA K48-linked polyubiquitination, thereby upregulating the protein levels of PCNA in HCC cells, whereas NUB1 overexpression inhibited PCNA NEDDylation and increased PCNA K48-linked polyubiquitination, thereby decreasing PCNA protein levels in HCC cells (Fig. 6B and Supplementary Fig. 4B).

Next, our results further confirmed that NEDD8 is required for NUB1-mediated disorder of PCNA NEDDylation and K48-linked polyubiquitination in HCC cells. Rescue experiments found that NUB1 knockdown increased the PCNA NEDDylation, inhibited the PCNA K48-linked polyubiquitination, and promoted the PCNA expression, whereas NEDD8 knockdown attenuated these effects of NUB1 knockdown in HCCLM3 cells (Fig. 6C). Conversely, NUB1 overexpression inhibited the PCNA NEDDylation, promoted the PCNA K48-linked polyubiquitination, and downregulated the PCNA expression, while NEDD8 overexpression reversed these effects caused by NUB1 overexpression in MHCC97H cells (Supplementary Fig. 4C). Furthermore, co-IP and western blotting experiments showed that TAS4464 (100 nM, 12 h) attenuated the decreased PCNA NEDDylation and increased PCNA K48-linked polyubiquitination and PCNA expression caused by NUB1 overexpression in HCCLM3 cells (Fig. 6D). Similar results were observed in MHCC97H cells treated with TAS4464 (Supplementary Fig. 4D).

Finally, our results confirmed that NEDD8 is essential for NUB1-mediated disorder of PCNA NEDDylation and K48-linked polyubiquitination in HCC cells. We constructed NEDD8^-/-^ HCCLM3 and NEDD8^-/-^ MHCC97H cell lines using CRISPR-Cas9 technology. After altering NUB1 expression in NEDD8^-/-^ HCC cells, PCNA NEDDylation was not detected, and there was no effect on PCNA K48-linked polyubiquitination or its expression (Fig. 6E and Supplementary Fig. 4E). However, after transfecting the NEDD8 plasmid into NEDD8^-/-^ HCC cell lines, compared to the control group, the shNUB1 group showed increased PCNA NEDDylation, decreased PCNA K48-linked polyubiquitination, and increased PCNA expression, whereas the Flag-NUB1 group showed the opposite results (Fig. 6F and Supplementary Fig. 4F). Overall, our results confirmed that NUB1 reduction can upregulate NEDD8 protein, thereby increasing PCNA NEDDylation to antagonize PCNA K48-linked polyubiquitination, which in turn promotes PCNA expression in HCC cells.

### TAS4464 inhibits the PCNA NEDDylation to suppress HCC cell growth in vitro and in vivo

**Fig. 7:**
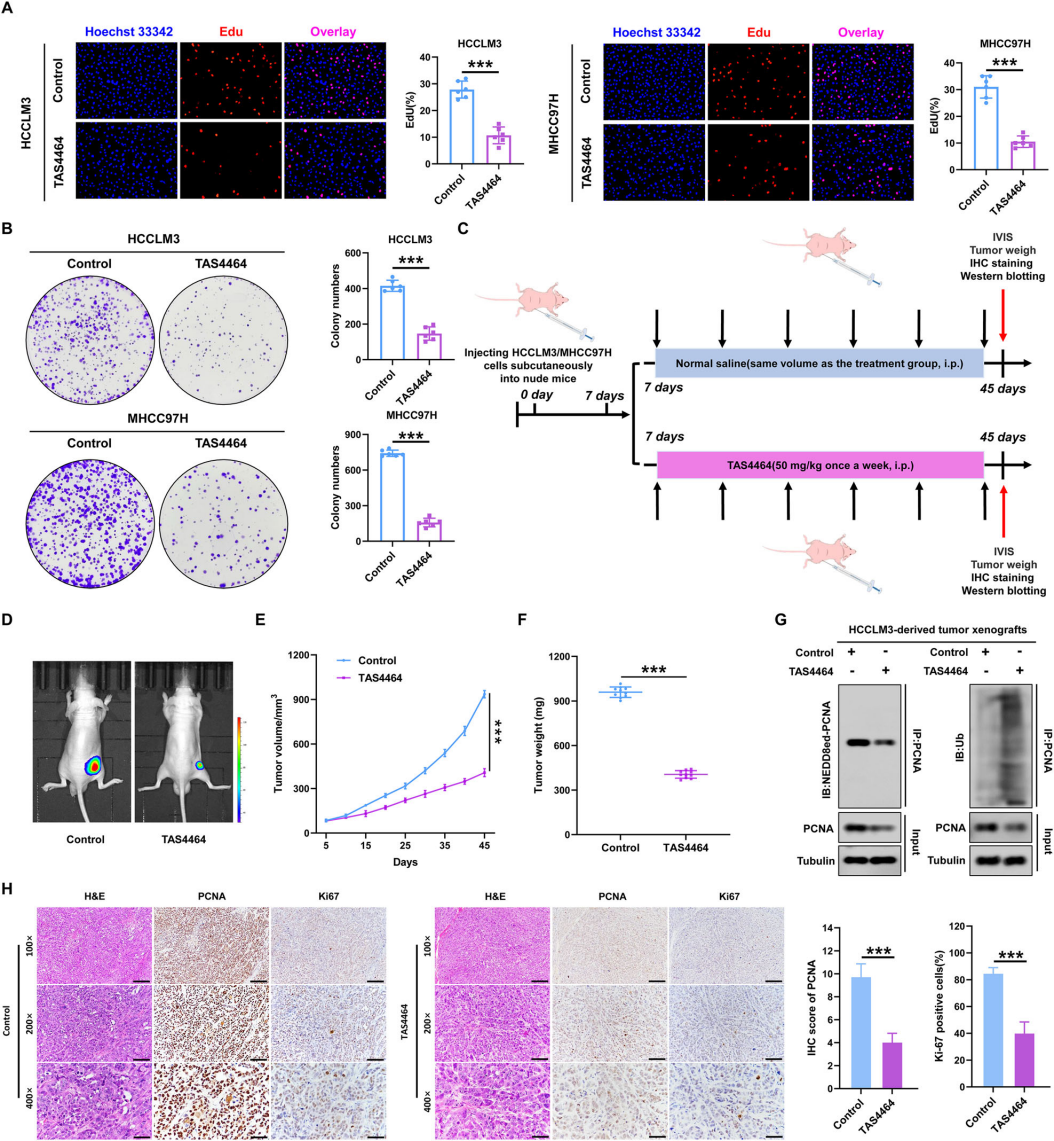
TAS4464 inhibits the PCNA NEDDylation to suppress HCC cell growth in vitro and in vivo. **A**, **B** EdU(**A**) and colon formation assays(**B**) detected the proliferation capacities of HCCLM3 cells with or without the 100 nM TAS4464. **C** Schematic diagram of evaluating the effect of TAS4464 in nude mice injecting HCCLM3/MHCC97H cells. **D** Representative bioluminescent images in nude mice subcutaneously injected with HCCLM3 cells treated with or without 50 mg/kg TAS4464 once a week (*n* = 10). **E**, **F** Tumor volumes (**E**) and weights (**F**) in nude mice intravenously injected with HCCLM3 cells treated with or without 50 mg/kg TAS4464 (*n* = 10). **G** The cell lysates of HCCLM3-derived tumor xenografts treated with or without TAS4464 were immunoprecipitated with anti-PCNA antibody, followed by immunoblotting. **H** Representative immunohistochemical staining photograph of NUB1, PCNA, and Ki67 in the HCCLM3-derived tumor xenografts treated with or without TAS4464. Scale bars = 200 µm, 100 µm and 50 µm. Data represent the mean ± SD. *p < 0.05; **p < 0.01; ***p < 0.001.

Based on the important role of PCNA NEDDylation in regulating PCNA expression, we investigated whether TAS4464 could inhibit PCNA NEDDylation to decrease PCNA expression, thereby suppressing HCC cell growth in vitro and in vivo. Our in vitro experiments revealed that TAS4464 significantly inhibited the proliferation of HCC cells. We observed the effect of TAS4464 treatment (100 nM) on HCCLM3 and MHCC97H cell growth in vitro using the EdU assay. The results showed that HCC cell proliferation was significantly lower in the TAS4464-treated group compared to the control group (Fig. 7A). Similarly, colony formation assays also confirmed that, compared with the control group, the proliferative ability of HCC cells was significantly inhibited in the TAS4464 treated group (Fig. 7B).

Furthermore, in vivo experiments confirmed that TAS4464 treatment significantly suppressed HCC cell growth by inhibiting PCNA NEDDylation and expression. We established a subcutaneous tumor model in BALB/c nude mice by injecting HCCLM3 and MHCC97H cells. After 1 week, we administered TAS4464 treatment to the above models, with one subset receiving TAS4464 treatment (50 mg/kg, once a week) and the other treated with saline (Fig. 7C). Fourty-five days after HCC cell injection, IVIS imaging revealed that treatment with TAS4464 significantly inhibited tumor growth compared with that in the control group in HCCLM3 cells (Fig. 7D). Similarly, the volume and weight of tumors formed by HCCLM3 cells were significantly decreased in the TAS4464 treated group compared to those in the control group at the end of the experiment (Fig. 7E, F).

Finally, we detected the changes in PCNA NEDDylation, PCNA polyubiquitination, and PCNA protein expression in HCCLM3-derived tumor xenografts after TAS4464 treatment. Our data showed that PCNA NEDDylation was significantly reduced, whereas its polyubiquitination was significantly increased in the TAS4464 treated group compared to the control group (Fig. 7G). IHC staining of the subcutaneous tumor tissue formed by HCCLM3 cells revealed that TAS4464 treatment significantly reduced PCNA protein expression and the number of Ki-67-positive cells (Fig. 7H). Similar results were observed in the MHCC97H-derived tumor xenografts (Supplementary Fig. 5A–E). Overall, our results indicated that TAS4464 treatment inhibits PCNA NEDDylation to downregulate PCNA expression, thereby inhibiting HCC growth in vitro and in vivo.

## Discussion

**Fig. 8:**
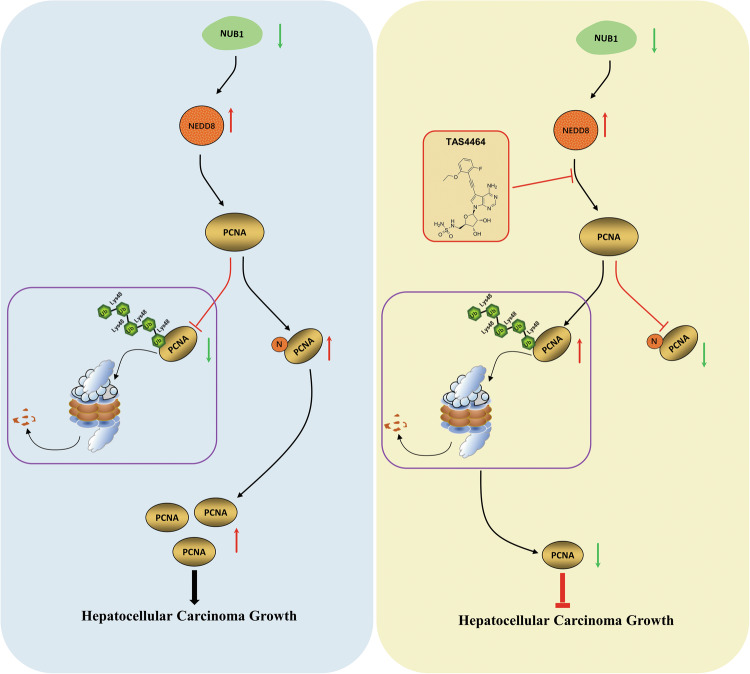
A cartoon summarizing our findings. NUB1 reduction upregulated NEDD8 to promote PCNA NEDDylation at lysine164, antagonized PCNA K48-linked polyubiquitination, leading to the decrease of PCNA degradation by UPS and promoted HCC cell growth. Targeting PCNA NEDDylation with TAS4464 in the treatment of HCC has great clinical potential.

HCC growth is a complex process involving oncogene activation and tumor suppressor gene inactivation, which have important effects on the survival of patients with HCC [32]. Although the critical role of oncogenes in promoting HCC growth has been well-studied in numerous studies, research on tumor suppressor genes in HCC growth remains relatively insufficient [33]. Therefore, the identification of new tumor suppressor genes in HCC are important for an in-depth understanding of the mechanisms of HCC proliferation. NUB1 was initially identified as a negative regulator of NEDD8 and was confirmed to be expressed in various tumor cell lines [14]. However, there is confusion about the role of NUB1 in tumors. For example, studies have revealed that NUB1 is a positive regulator of tumor proliferation in breast cancer. NUB1 is highly expressed in breast cancer, and its knockdown of NUB1 has been demonstrated to inhibit breast cancer cell proliferation [12]. Conversely, NUB1 functions as a negative regulator of tumor proliferation in some other tumors. Studies have demonstrated that NUB1 overexpression can inhibit the growth of a wide range of tumors, including renal cell carcinoma, osteosarcoma, and gastric cancer, by inhibiting NEDD8 and its conjugation system [13–15]. However, the expression and role of NUB1 in HCC remain to be elucidated. In this study, our data revealed that NUB1 protein expression was reduced in HCC tissues and cells. In vitro and in vivo experiments further confirmed that NUB1 knockdown promotes HCC cell growth by upregulating PCNA protein expression. Conversely, NUB1 overexpression inhibits HCC cell growth by decreasing PCNA protein expression. Mechanistically, NUB1 reduction upregulated NEDD8 protein to promote PCNA NEDDylation at lys164, which antagonizes PCNA K48-linked polyubiquitination and inhibits its degradation by the UPS. Overall, our results confirmed that NUB1 functioned as a negative regulator of HCC proliferation and revealed its mechanism of action (Fig. 8).

Proteins are the major performers of cellular biological activities, and various PTMs, including ubiquitination, ubiquitin-like modifications, and modifications of other proteins and chemical groups, commonly regulate their expression and function [34]. Among them, K48-linked polyubiquitination primarily mediates substrate protein degradation by the UPS [35]. PCNA is a core component of eukaryotic DNA replication and repair, and diverse PTMs regulate its stability and function [27]. Dysregulated PCNA polyubiquitination caused by the deubiquitinase USP10 or E3 ubiquitin ligases, such as HECW2 and SYVN1, regulates its stability. In contrast, monoubiquitination, phosphorylation, and S-nitrosylation of PCNA can affect its interaction with other proteins to regulate DNA damage repair and apoptosis, respectively [16, 24, 36, 37]. Recent studies have confirmed that ubiquitin-like modifications of PCNA can also regulate its function. For example, PCNA SUMOylation recruits PARI to inhibit homologous recombination (HR), and PCNA ISGylation recruits USP10 to inhibit PCNA monoubiquitination and DNA damage repair [16]. In addition, a study has confirmed that PCNA is also a substrate of NEDDylation, and PCNA NEDDylation can antagonize PCNA monoubiquitination to affect its function [29]. However, as the important type of PTMs, ubiquitin-like modifications of PCNA whether affect the protein expression of PCNA remains unclear. Interestingly, our results confirmed that PCNA NEDDylation regulates PCNA protein stability. First, Co-IP and confocal immunofluorescence assays demonstrated that the NEDD8 protein could bind to the PCNA protein in HCC cells. Second, NEDD8 overexpression upregulated PCNA expression by increasing PCNA NEDDylation to inhibit PCNA K48-linked polyubiquitination in HCC cells. Third, NEDD8 can compete with Ub for binding to PCNA, and PCNA NEDDylation can antagonize PCNA K48-linked polyubiquitination in HCC cells. To the best of our knowledge, this study is the first to demonstrate that PCNA NEDDylation regulates PCNA protein stability in addition to its function, thereby broadening our understanding of the regulatory role of NEDDylation in substrate protein expression and function.

Based on the important role of NEEDylation in tumor development through the regulation of protein expression and function, NEEDylation inhibitors have become a promising strategy for tumor therapy [28]. Therefore, the identification of new targets for NEDDylation inhibitors is of great significance to understanding the role of NEDD inhibitors in tumor treatment. For example, NEDDylation inhibitors MLN4924 and TAS4464 can disrupt CRL-mediated protein turnover, causing S-phase arrest and apoptosis in colorectal cancer cells and c-Myc-mediated apoptosis in acute myeloid leukemia cells, respectively [38, 39]. In addition, NEDDylation inhibitors have shown good antitumor effects in HCC. Previous research points out that MLN4924 has been shown to inhibit the NEDDylation of cullin family proteins to activate ERK, in turn impairing the HNF1α-C/EBPα-HNF4α axis and suppressing the survival of HCC cells [40]. MLN4924 was also shown to enhance the sensitivity of HCC cells to sorafenib treatment by inhibiting the activity of CRL/Skp1‑Cullin1‑F box (SCF) [41]. In this study, we identified novel targets of NEDDylation inhibitors that inhibit HCC growth. Our data showed that TAS4464 directly inhibited PCNA NEDDylation to promote PCNA degradation by the UPS, thereby decreasing PCNA protein expression and ultimately leading to a decline in HCC growth. This study offers a novel perspective on the mechanism of NEDDylation inhibitors against HCC and provides theoretical support for their future clinical application in other tumors.

In conclusion, our study confirmed that NUB1 functions as a negative regulator of HCC proliferation and elucidated its mechanism of action in HCC progression. Importantly, our study also confirmed that, in addition to regulating the function of PCNA, PCNA NEDDylation can also regulate the protein stability of PCNA. Future in-depth studies are necessary to investigate whether NEDDylation regulates the expression of other substrate proteins in various tumor cells to influence tumor progression. These findings will not only provide a theoretical basis for understanding the role of NEDDylation in tumor progression but also provide new insights into the development of therapeutic approaches for patients with tumors, including HCC.

## Supplementary information

Supplementary Figure 1

Supplementary Figure 2

Supplementary Figure 3

Supplementary Figure 4

Supplementary Figure 5

Supplementary Table 1

Supplementary Table 2

Supplementary Figure Legends

Supplementary Materials and Methods

Original western blots
