# Supplementary figures and images for: NUB1 reduction promotes PCNA-mediated tumor growth by disturbing the PCNA polyubiquitination/NEDDylation in hepatocellular carcinoma cells
Source: Cell Death Dis. 2025 Mar 31;16(1):228. doi: 10.1038/s41419-025-07567-3 (PMC11958677; doi:10.1038/s41419-025-07567-3)

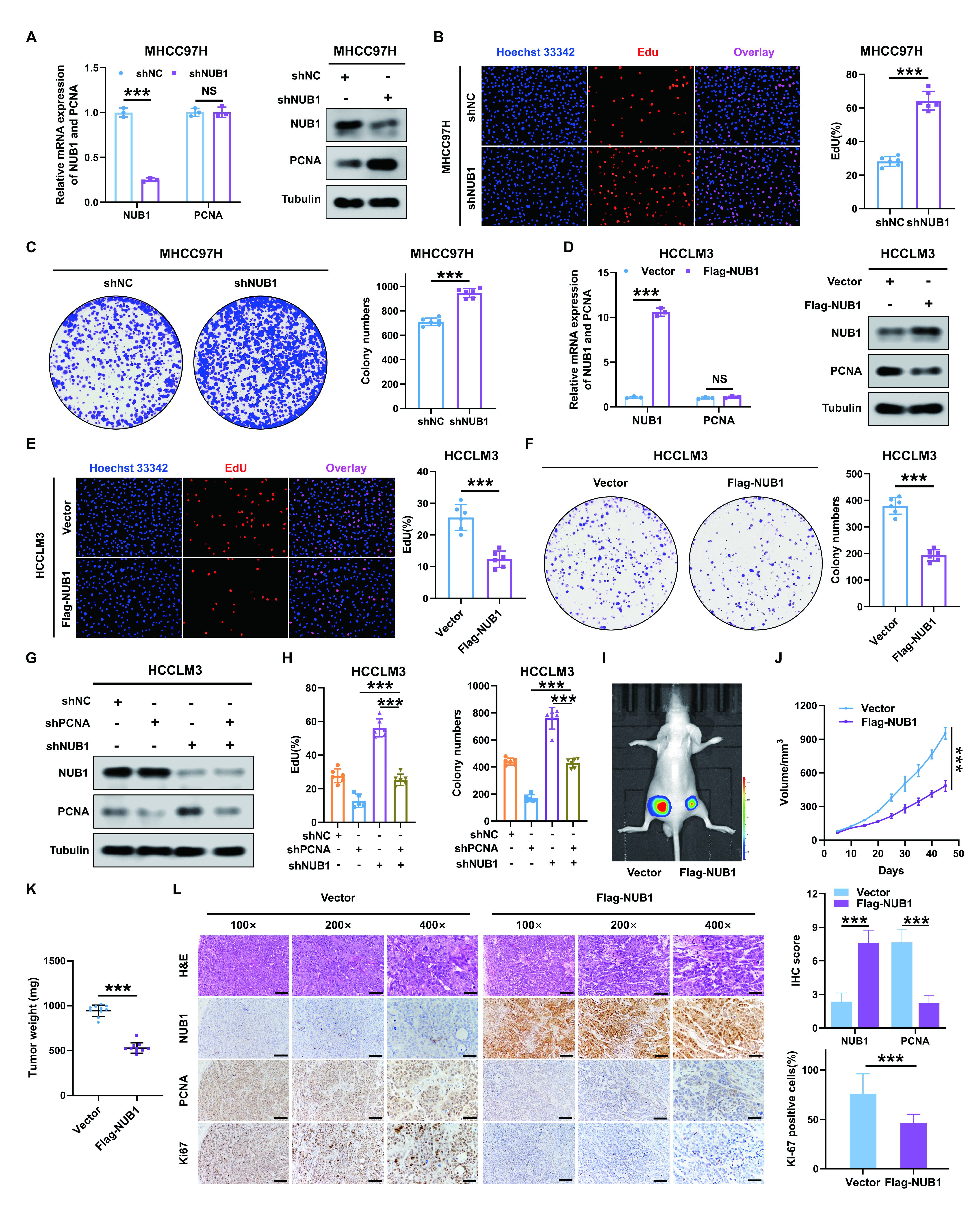

Supplement: Supplementary file 1 — Supplementary Figure 1 [file 41419_2025_7567_MOESM1_ESM.tif]

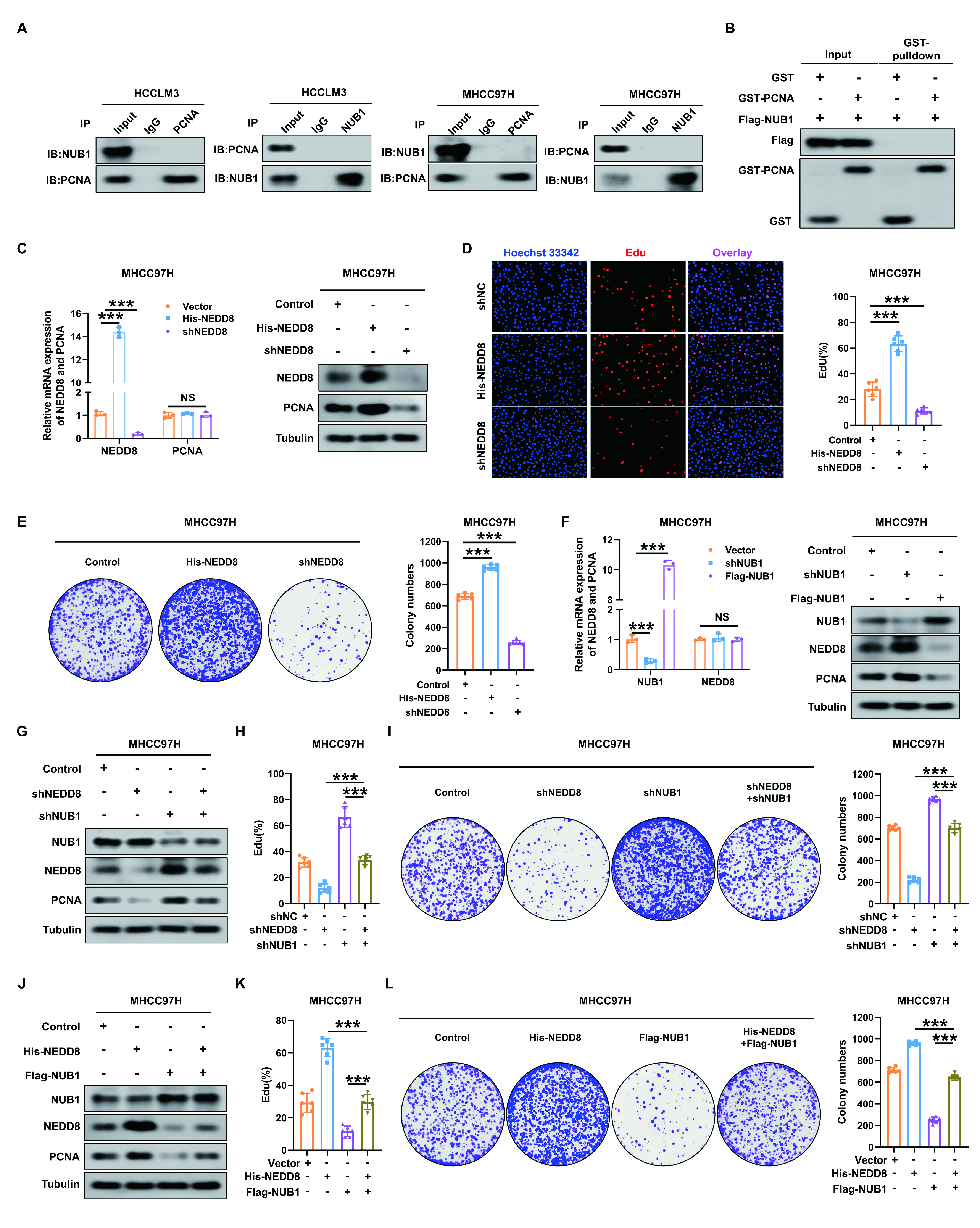

Supplement: Supplementary file 2 — Supplementary Figure 2 [file 41419_2025_7567_MOESM2_ESM.tif]

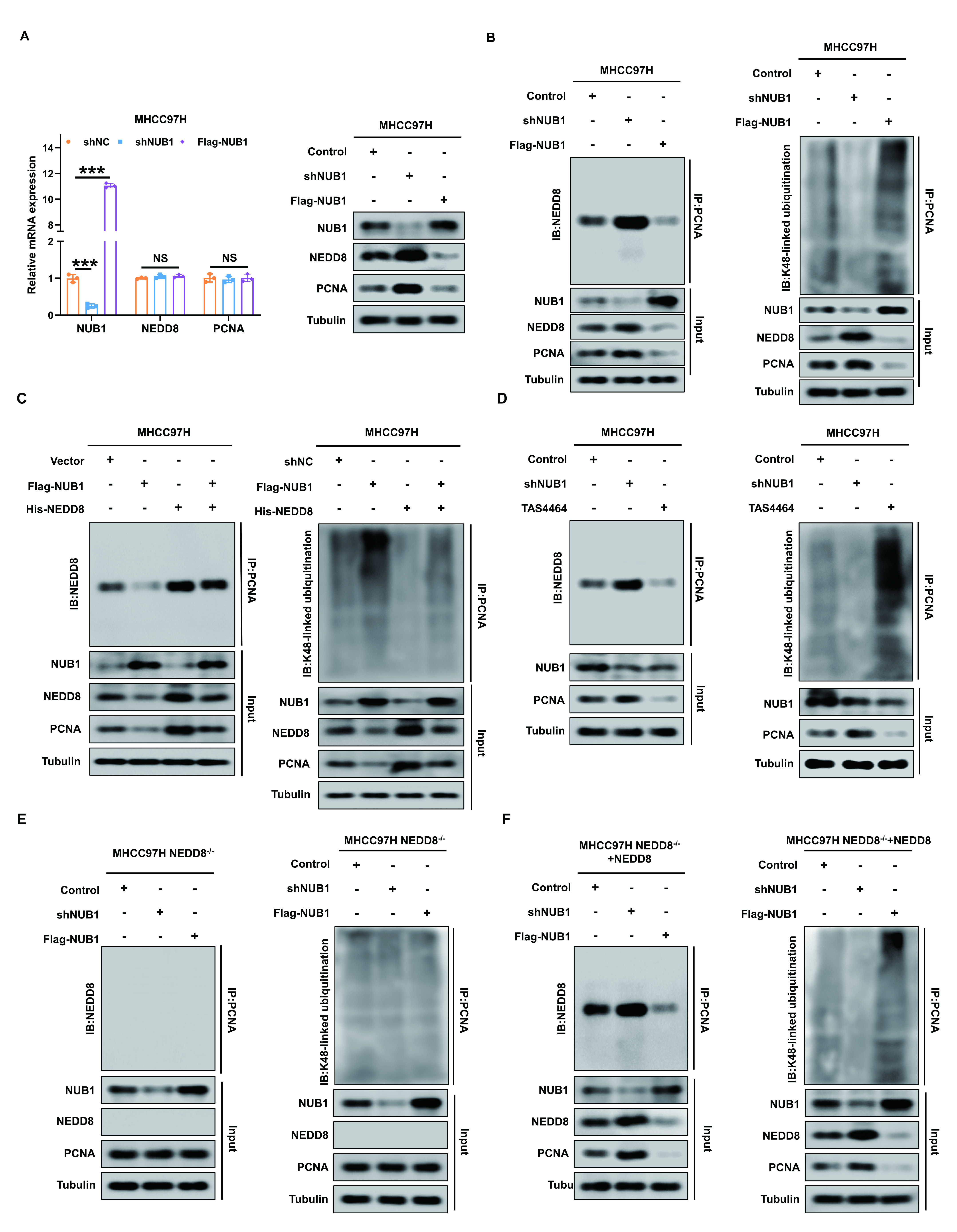

Supplement: Supplementary file 4 — Supplementary Figure 4 [file 41419_2025_7567_MOESM4_ESM.tif]

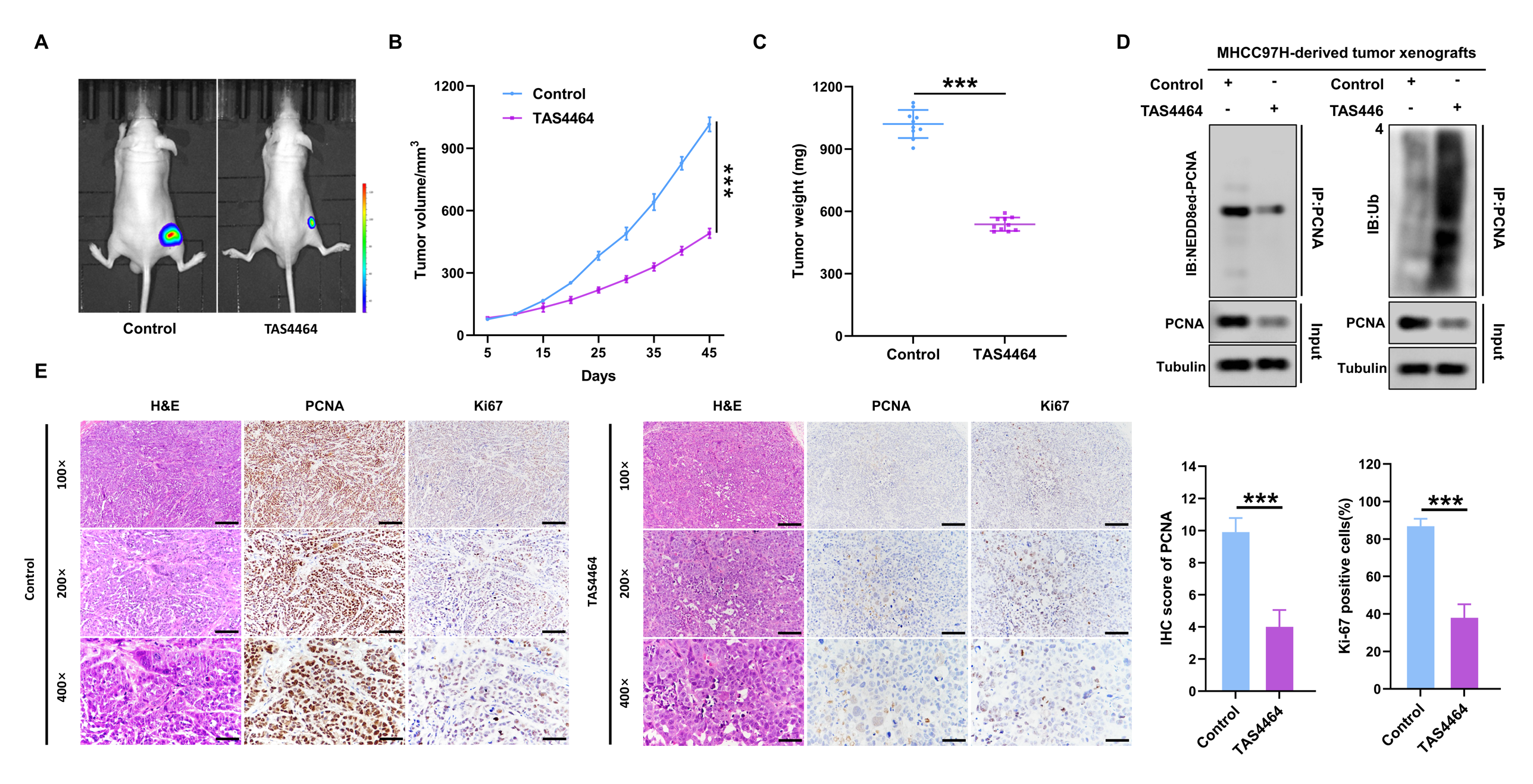

Supplement: Supplementary file 5 — Supplementary Figure 5 [file 41419_2025_7567_MOESM5_ESM.tif]
