# Supplementary material for: NUB1 reduction promotes PCNA-mediated tumor growth by disturbing the PCNA polyubiquitination/NEDDylation in hepatocellular carcinoma cells
Source: Cell Death Dis. 2025 Mar 31;16(1):228. doi: 10.1038/s41419-025-07567-3 (PMC11958677; doi:10.1038/s41419-025-07567-3)
Supplement: Supplementary file 6 — Supplementary Table 1 [file 41419_2025_7567_MOESM6_ESM.docx]

**Supplementary Table 1 List of shRNA sequences used for gene knockdown**

| Gene name | Target sequence |
| --- | --- |
| NUB1 | GGACTAGAAATACTGGCAA |
| NEDD8 | GAGAAGACAGCAGCTGATT |
| PCNA | TACACTAAGGGCCGAAGATAA |
