# Supplementary material for: NUB1 reduction promotes PCNA-mediated tumor growth by disturbing the PCNA polyubiquitination/NEDDylation in hepatocellular carcinoma cells
Source: Cell Death Dis. 2025 Mar 31;16(1):228. doi: 10.1038/s41419-025-07567-3 (PMC11958677; doi:10.1038/s41419-025-07567-3)
Supplement: Supplementary file 7 — Supplementary Table 2 [file 41419_2025_7567_MOESM7_ESM.docx]

**Supplementary Table 2 Primers used in this study**

| Gene name | Primer sequence |
| --- | --- |
| NUB1 |  |
| F (5'to3') | AGGATTCAACTTTGGAAACCTCC |
| R (5'to3') | ACAGCATTCTAGTCTGTCAGAGT |
| NEDD8 |  |
| F (5'to3') | ATGCTAATTAAAGTGAAGAC |
| R (5'to3') | TCCTCCTCTCAGAGCCAACAC |
| PCNA |  |
| F (5'to3') | AGCCACTCCACTCTCTTCAACG |
| R (5'to3') | TTCATCCTCGATCTTGGGAGCC |
| GAPDH |  |
| F (5'to3') | CTTCATTGACCTCAACTACA |
| R (5'to3') | ACTCCACGACATACTCAGC |
