# Supplementary material for: NUB1 reduction promotes PCNA-mediated tumor growth by disturbing the PCNA polyubiquitination/NEDDylation in hepatocellular carcinoma cells
Source: Cell Death Dis. 2025 Mar 31;16(1):228. doi: 10.1038/s41419-025-07567-3 (PMC11958677; doi:10.1038/s41419-025-07567-3)
Supplement: Supplementary file 8 — Supplementary Figure Legends [file 41419_2025_7567_MOESM8_ESM.pdf]

Supplementary Fig.1 NUB1 overexpression could downregulate PCNA expression to suppress HCC cell growth in vitro and in vivo. A. MHCC97H cells were transfected with shRNA against NUB1 and the cell lysates were subjected to qRT-PCR and immunoblotting (n=3). The mRNA levels of NUB1 and PCNA were normalized to GAPDH. B and C. EdU (B) and colon formation assays (C) detected the proliferation capacities of MHCC97H cells with the indicated treatments (n=6). D. HCCLM3 cells were transfected with NUB1 overexpression lentivirus and the cell lysates were subjected to qRT-PCR and immunoblotting (n=3). The mRNA levels of NUB1 and PCNA were normalized to GAPDH. E and F. EdU (E) and colon formation assays (F) detected the proliferation capacities of HCCLM3 cells with the indicated treatments (n=6). G. Determination of the protein levels of NUB1 and PCNA by immunoblotting with the indicated treatments in HCCLM3 cells. H. EdU and colon formation assays detected the proliferation capacities of HCCLM3 cells with the indicated treatments (n=6). I. Representative bioluminescent images in nude mice subcutaneously injected with Flag-vector cells or Flag-NUB1 HCCLM3 cells (n=10). J and K. Tumor volumes (J) and weights (K) in nude mice intravenously Flag-vector cells or Flag-NUB1 HCCLM3 cells (n=10). L. IHC analysis of NUB1 and PCNA expression levels and Ki-67 positive cell numbers in subcutaneous tumors in the above groups. Scale bars=200 μm, 100 μm and 50μm. Data represent the mean±SD. *$p<0.05$; **$p<0.01$; ***$p<0.001$.

Supplementary Fig.2 NUB1 reduction promotes PCNA protein expression by increasing NEDD8 in HCC cells. A. co-IP between endogenous NUB1 and PCNA in HCCLM3 and MHCC97H cells. B. GST-pull down experiments were performed with the indicated fusion proteins. C. qRT-PCR and western blot were used to analyze the mRNA and protein levels of NEDD8 and PCNA in MHCC97H cells with the indicated treatments (n=3). The mRNA levels of NEDD8 and PCNA were normalized to GAPDH. D and E. EdU (D) and colon formation assays (E) detected the proliferation capacities of MHCC97H cells with the indicated treatments (n=6). F. qRT-PCR and western blot were used to analyze the mRNA and protein levels of NUB1 and NEDD8 in MHCC97H cells with the indicated treatments(n=3). The mRNA levels of NUB1 and NEDD8 were

normalized to GAPDH. G. MHCC97H cells transfected with shNEDD8 or control were transfected with or without shNUB1 and the cell lysates were subjected to immunoblotting. H and I. EdU(H) and colon formation assays(I) analyses for the above groups (n=6). J. MHCC97H cells transfected with His-NEDD8 or control were transfected with or without Flag-NUB1 and the cell lysates were subjected to immunoblotting. K and L. EdU (K) and colon formation assays (L) analyses for the above groups (n=6). Data represent the mean±SD. *p<0.05; **p<0.01; ***p<0.001.

Supplementary Fig.3 NEDD8-mediated PCNA NEDDylation antagonizes PCNA K48-linked polyubiquitination to promote PCNA expression in MHCC97H cells. A. co-IP between NEDD8 and PCNA in MHCC97H cells. B. Representative confocal immunofluorescence images of NEDD8 (red) colocalized with PCNA (green) in MHCC97H cells. C. HA-PCNA and HA-PCNA K164R plasmids were transfected into MHCC97H cells stably transfected with His-NEDD8, respectively, and co-IP detected the binding of the exogenous tag HA to His. D and E. MHCC97H cells were transfected with blank vector, His-NEDD8 or shNEDD8, followed by transfecting with HA-PCNA (D) or HA-PCNA K164R (E), and the cell lysates were immunoprecipitated with anti-HA antibody, followed by immunoblotting. F. MHCC97H cells transfected with increasing dose gradients of His-NEDD8, and the cell lysates were immunoprecipitated with anti-PCNA antibody, followed by immunoblotting. G. MHCC97H cells transfected with blank vector or His-NEDD8, and MHCC97H His-NEDD8 cells treated with TAS4464 (100nM) for 12 hours, and the cell lysates were subjected to Co-IP and immunoblotting.

Supplementary Fig.4 NUB1 reduction disturbs the PCNA NEDDylation and K48-linked polyubiquitination to increase PCNA by upregulating NEDD8 in MHCC97H cells. A. qRT-PCR and western blot were used to analyze the mRNA and protein levels of NUB1, NEDD8 and PCNA in MHCC97H cells with the indicated treatments(n=3). The mRNA levels of NUB1, NEDD8 and PCNA were normalized to GAPDH. B. MHCC97H cells were transfected with blank vector, shNUB1 and Flag-

NUB1, respectively, and then the cell lysates were immunoprecipitated with anti-PCNA antibody, followed by immunoblotting. C. MHCC97H cells transfected indicated plasmids and the cell lysates were immunoprecipitated with anti-PCNA antibody, followed by immunoblotting. D. MHCC97H cells transfected with shNC or shNUB1, and MHCC97H shNUB1 cells treated with TAS4464 (100nM) for 12 hours, and the cell lysates were subjected to Co-IP and immunoblotting. E. MHCC97H $NEDD8^{-/-}$ cells were transfected with blank vector, shNUB1 and Flag-NUB1, respectively, and then the cell lysates were immunoprecipitated with anti-PCNA antibody, followed by immunoblotting. F. Restoration of NEDD8 expression in MHCC97H $NEDD8^{-/-}$ cells and transfected with blank vector, shNUB1 and Flag-NUB1, respectively, and the cell lysates were immunoprecipitated with anti-PCNA antibody, followed by immunoblotting.

Supplementary Fig.5 TAS4464 treatment inhibits the growth of MHCC97H-derived tumors xenograft by inhibiting NEDDylation. A. Representative bioluminescent images in nude mice subcutaneously injected with MHCC97H cells treated with or without 50 mg/kg TAS4464 once a week (n=10). B and C. Tumor volumes (B) and weights (C) in nude mice intravenously injected with MHCC97H cells treated with or without 50 mg/kg TAS4464 (n=10). D. The cell lysates of MHCC97H-derived tumor xenografts treated with or without TAS4464 were immunoprecipitated with anti-PCNA antibody, followed by immunoblotting. E. Representative immunohistochemical staining photograph of NUB1, PCNA, and Ki67 in the MHCC97H-derived tumor xenografts treated with or without TAS4464. Scale bars=200 μm, 100 μm and 50μm. Data represent the mean±SD. *$p<0.05$; **$p<0.01$; ***$p<0.001$.
