# Supplementary material for: NUB1 reduction promotes PCNA-mediated tumor growth by disturbing the PCNA polyubiquitination/NEDDylation in hepatocellular carcinoma cells
Source: Cell Death Dis. 2025 Mar 31;16(1):228. doi: 10.1038/s41419-025-07567-3 (PMC11958677; doi:10.1038/s41419-025-07567-3)
Supplement: Supplementary file 9 — Supplementary Materials and Methods [file 41419_2025_7567_MOESM9_ESM.pdf]

Supplementary Materials and Methods

Immunohistochemical (IHC) staining

Tissues were fixed in 10% formalin, embedded in paraffin, and sectioned. The tissue sections were then dewaxed. Some of the dewaxed sections were stained with hematoxylin-eosin (H&E) to detect morphological changes, and others were blocked with 5% goat serum at room temperature for 30 min after antigen repair. Sections were subsequently incubated overnight at 4°C with the corresponding primary antibody, labeled with an EnVision HRP kit (Solarbio) for 30 min at room temperature, incubated with DAB substrate solution (Solarbio), and counterstained with Mayer's hematoxylin (Solarbio). All sections were observed and photographed using a light microscope and DP70 CCD system (Olympus Corp.). The staining intensity and percentage of positive cells were scored semi-quantitatively by two pathologists who were blinded to the clinical parameters.

Plasmids and construction of CRISPR–Cas9 knockout cell lines

The shNUB1 lentivirus, shPCNA lentivirus and shNEDD8 lentivirus were synthesized by GeneChem (Shanghai, China). The target sites of shRNA are detailed in Supplementary Table 1. NUB1 (NM_001363529.2), PCNA (NM_002592.2), and NEDD8 (NM_006156.3) overexpression lentivirus and blank vector lentivirus were purchased by GeneChem, Shanghai, China. The CRISPR–Cas9 plasmid was purchased from Santa Cruz Biotechnology and transfected using lipofection, with *GFP* as the reporter gene. To enrich the transfected cells, they were screened using puromycin. Stable surviving cells were sorted into monoclonal clones, cultured for DNA isolation,

and genotyped separately. Based on the genotyping results, the monoclonal cells were amplified and propagated. The knock-out efficiency was finally verified via WB and DNA sequencing.

Quantitative real-time PCR (qRT-PCR)

Total RNA was extracted from cells collected after treatment using Trizol (Invitrogen, Carlsbad, CA, USA) and purified using TB Green Premix Ex Taq II (Takara Bio Inc. Kusatsu, Shiga, Japan). cDNA was reverse transcribed using the PrimeScript RT reagent Kit (Takara Bio Inc). Experiments were performed according to the instructions of the kit. The primer sequences used in this experiment are summarized in Supplementary Table 2.

Glutathione S-Transferase (GST) pull-down assay

The PierceTM GST Protein Interaction Pull-Down Kit (Thermo Scientific) was used to perform GST pull-down assay. Purified proteins were incubated with glutathione agarose beads overnight at 4°C in the buffer. After washing at least three times with the same buffer, the proteins were eluted using 4× SDS-PAGE loading buffer (Takara) and boiled. After separation on an SDS-PAGE gel, WB was performed as described above.

Immunofluorescence assay

HCCLM3 and MHCC97H cells were washed with cold phosphate-buffered saline (PBS; pH 7.4) and fixed with 4% paraformaldehyde for 30 min at room temperature. Then, the cells were permeated with 0.15% Triton X-100 for 20 min and blocked with blocking buffer (PBS supplemented with 5% goat serum) for 30 min at 37°C. Cells were fixed and incubated with the primary antibody overnight at 4°C, then incubated

the following day with the fluorescent dye-coupled secondary antibody for 2 h and DAPI for 10 min. Images were obtained using an SF5 confocal microscope (Leica, USA) equipped with a 63-magnification oil objective. Immunofluorescence intensity was calculated using ImageJ software.

5-Ethynyl-2'-deoxyuridine (EdU) proliferation assay

EdU proliferation assays were performed using the EdU kit (Beyotime). First, treated cells were inoculated into 96-well plates at a density of 1×10^4 cells per well and incubated for 12 h. The remaining steps were performed according to the manufacturer's instructions. Images were obtained using an inverted fluorescence microscope (Olympus Corp.).

Colony formation assay

After collection, cells were counted and seeded at a density of 500/well in six-well plates. Cells were then grown in an incubator with fresh medium, which was changed every 2–3 days. After 14 days, colonies were fixed with methanol and stained with 0.5% crystal violet for 30 min. The experiment was repeated at least three times with three replicate wells set up, and the colonies were scored under a microscope.
