# Supplementary material for: NUB1 reduction promotes PCNA-mediated tumor growth by disturbing the PCNA polyubiquitination/NEDDylation in hepatocellular carcinoma cells
Source: Cell Death Dis. 2025 Mar 31;16(1):228. doi: 10.1038/s41419-025-07567-3 (PMC11958677; doi:10.1038/s41419-025-07567-3)

Fig1B

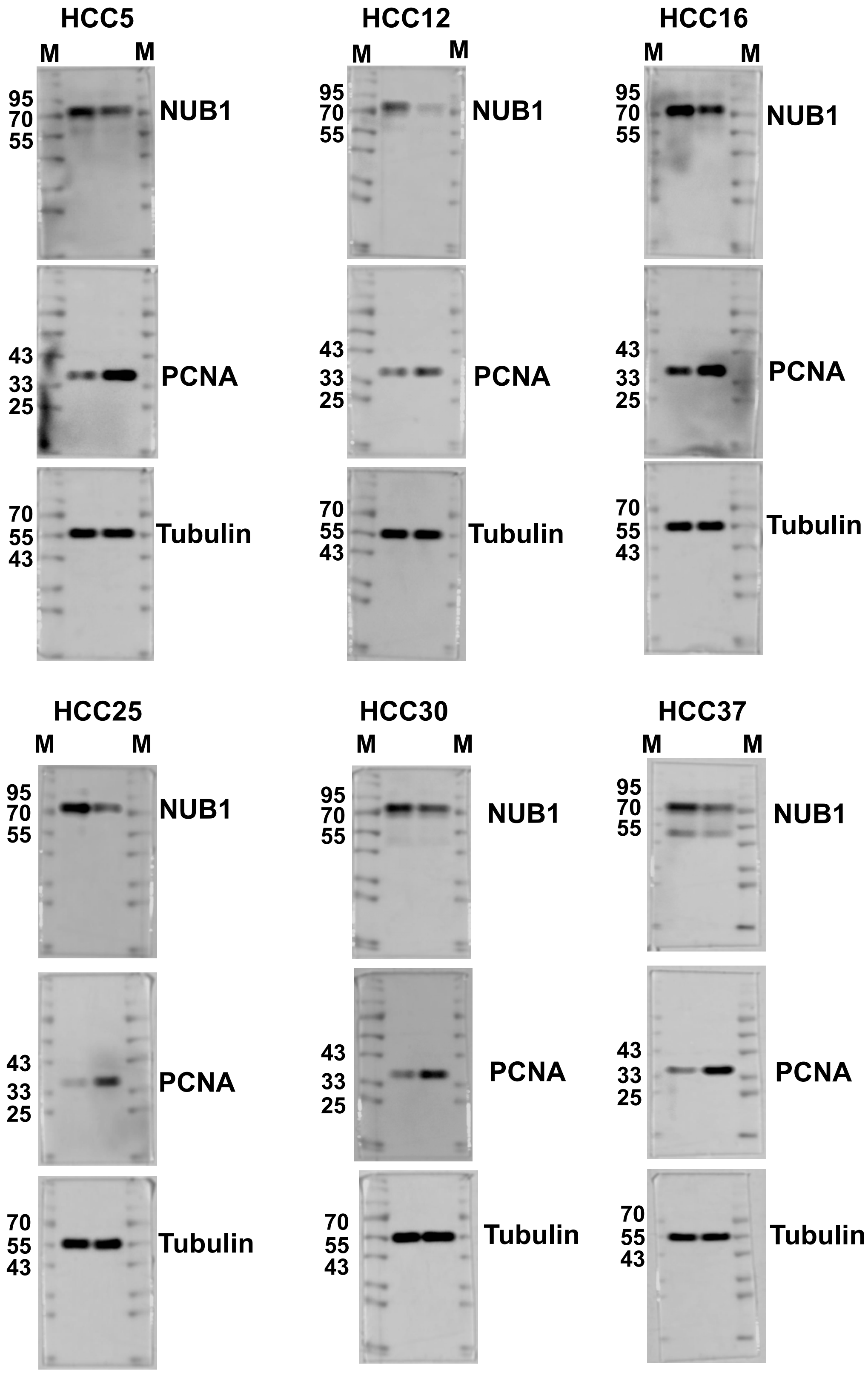
HCC5
M
M
95
70
55
NUB1
43
33
25
PCNA
70
55
43
Tubulin
HCC12
M
M
95
70
55
NUB1
43
33
25
PCNA
70
55
43
Tubulin
HCC16
M
M
95
70
55
NUB1
43
33
25
PCNA
70
55
43
Tubulin
HCC25
M
M
95
70
55
NUB1
43
33
25
PCNA
70
55
43
Tubulin
HCC30
M
M
95
70
55
NUB1
43
33
25
PCNA
70
55
43
Tubulin
HCC37
M
M
95
70
55
NUB1
43
33
25
PCNA
70
55
43
Tubulin

Fig1B

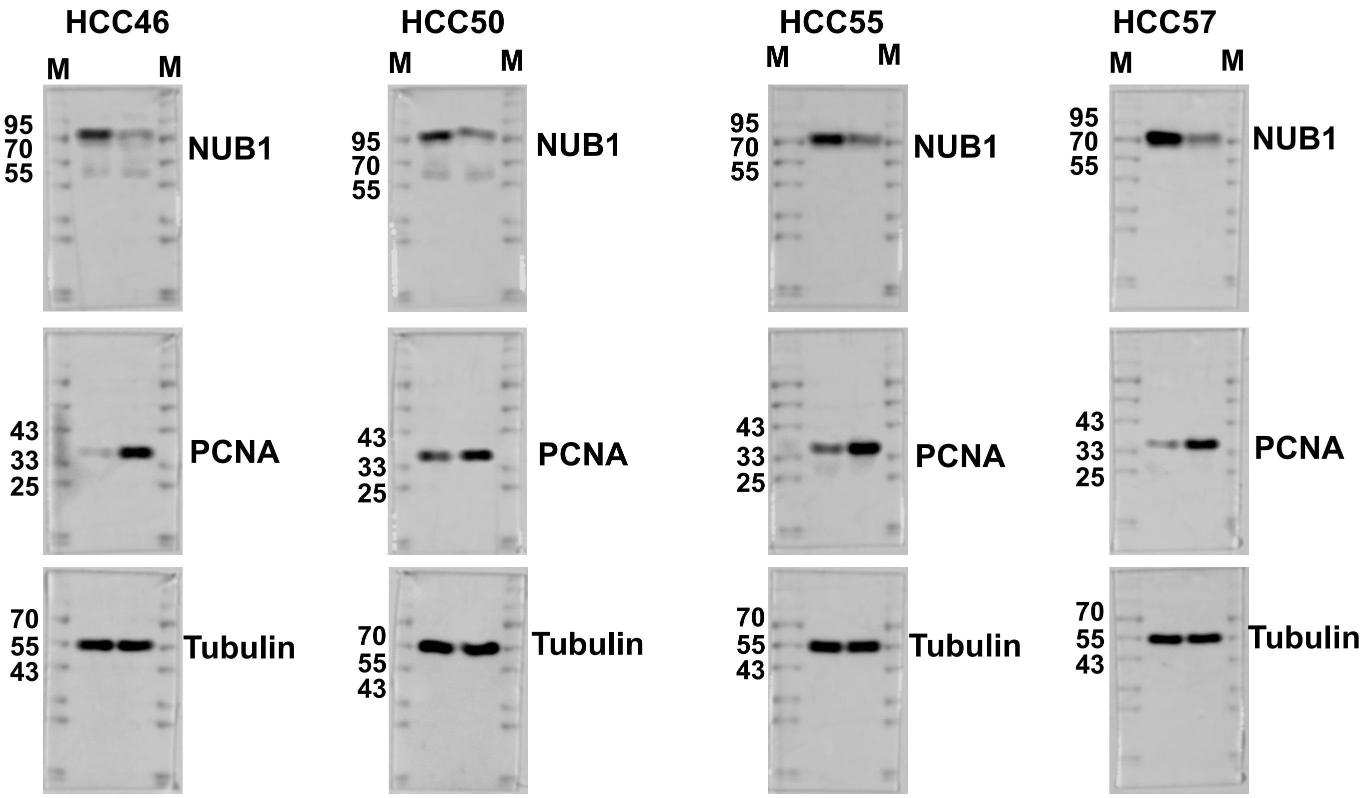

Fig1F

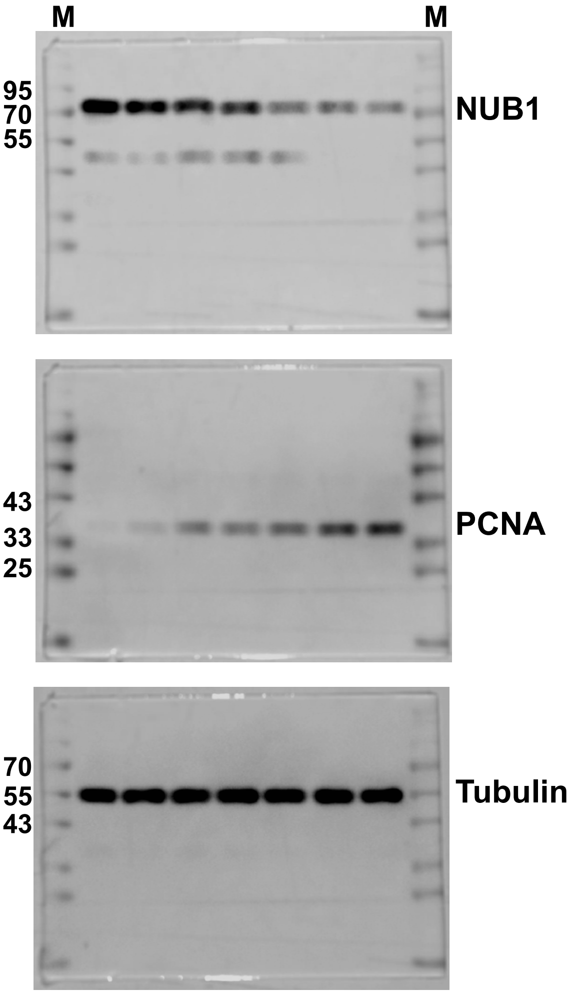

Fig2A

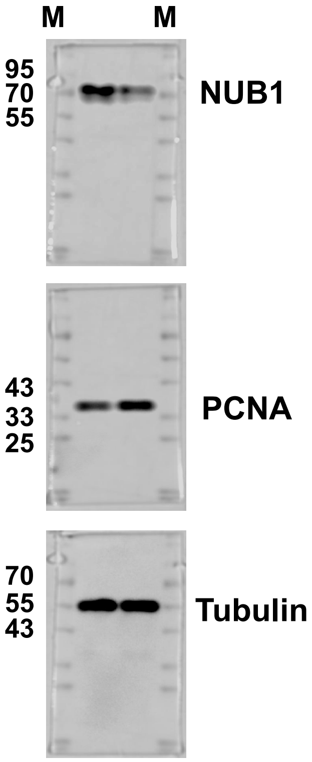

Fig2D

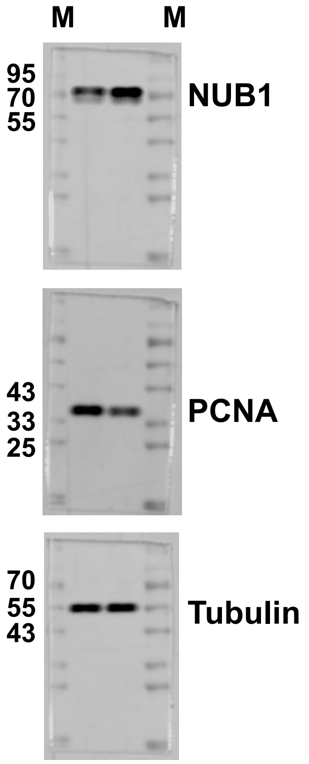

Fig2G

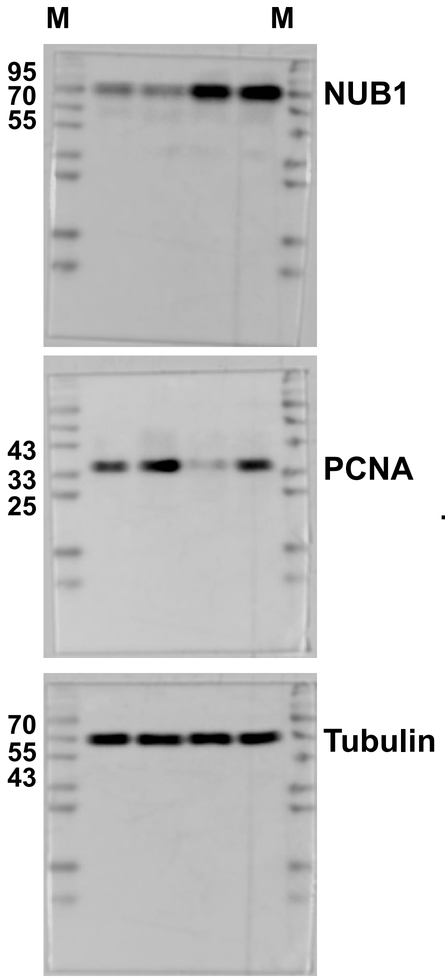

Fig3A-
HCCLM3

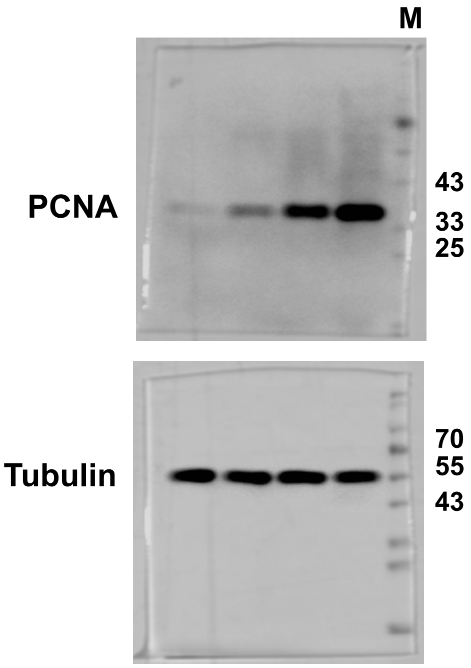

Fig3A-
MHCC97H

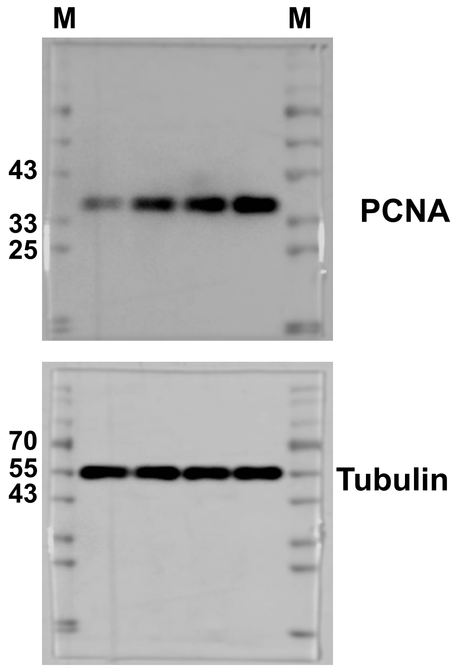

Fig3B-
HCCLM3

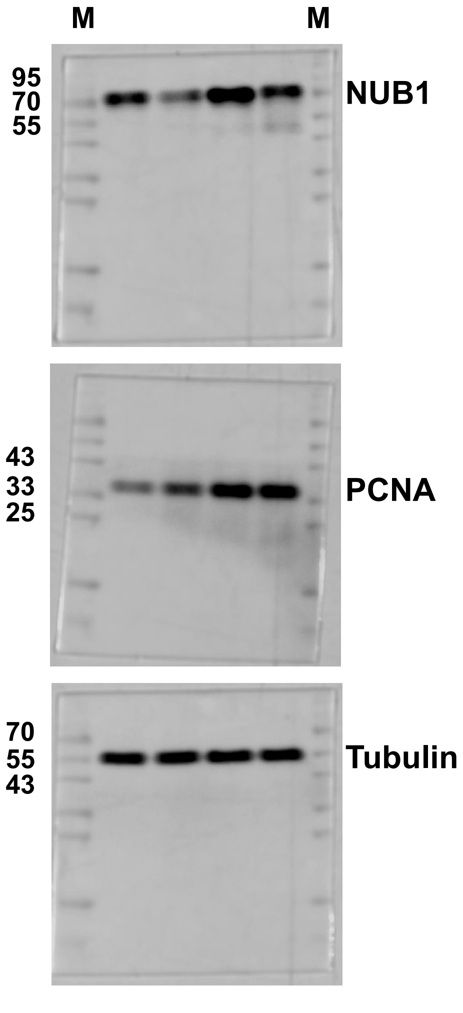

Fig3B-
MHCC97H

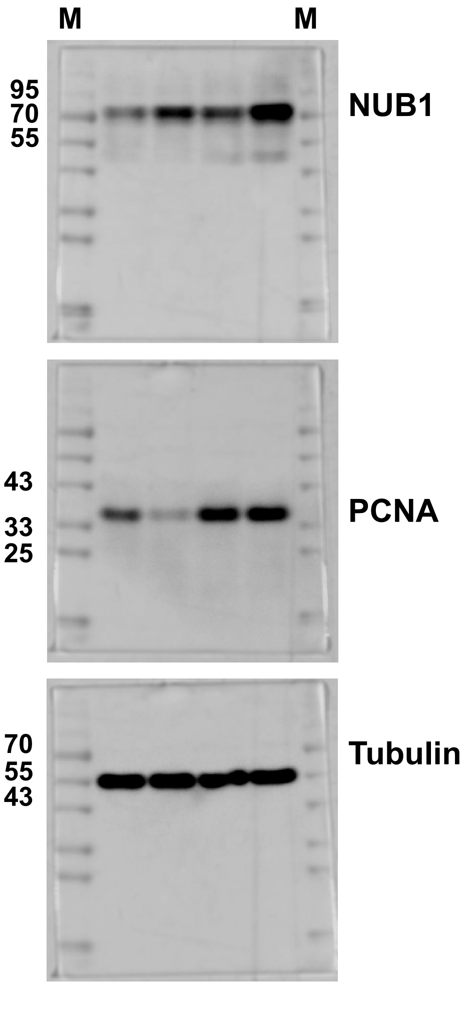

Fig3C-HCCLM3

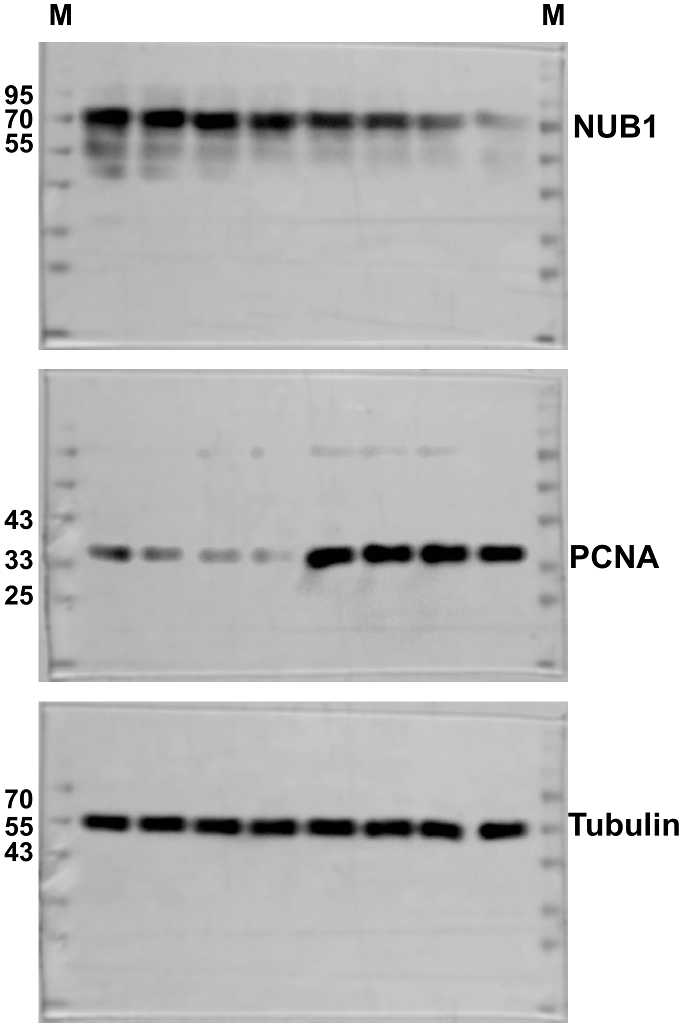

Fig3C-MHCC97H

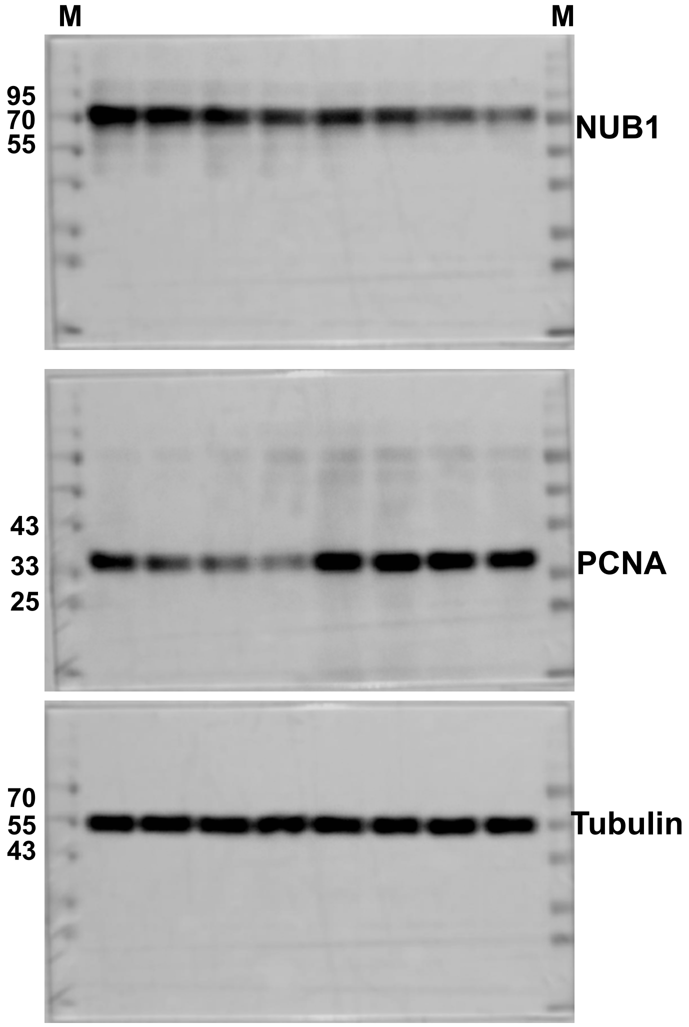

Fig3D-HCCLM3

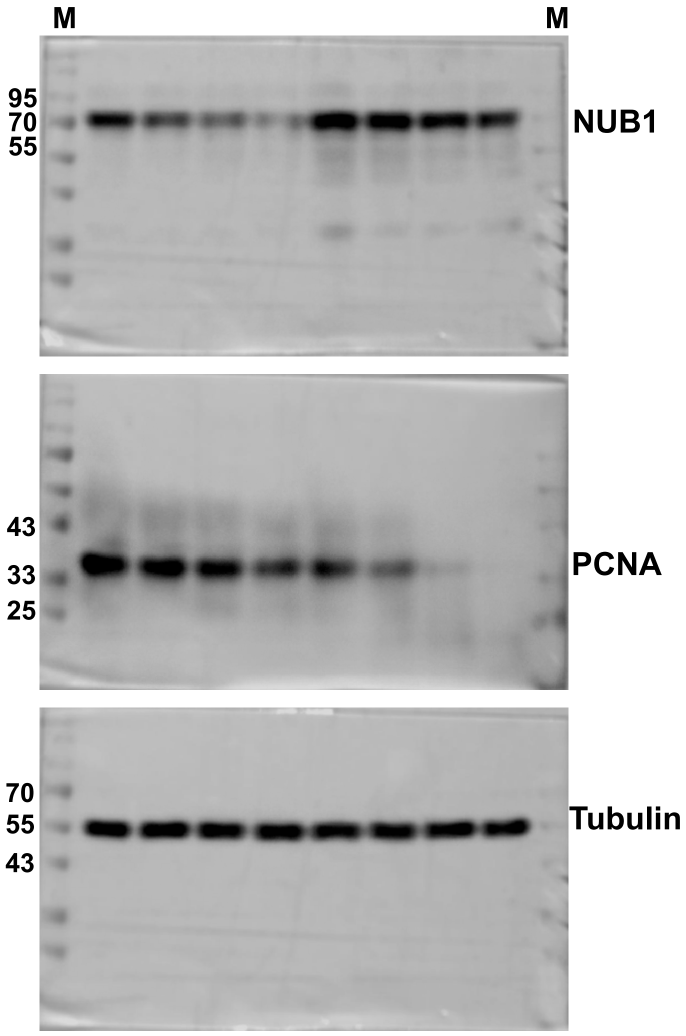

Fig3D-MHCC97H

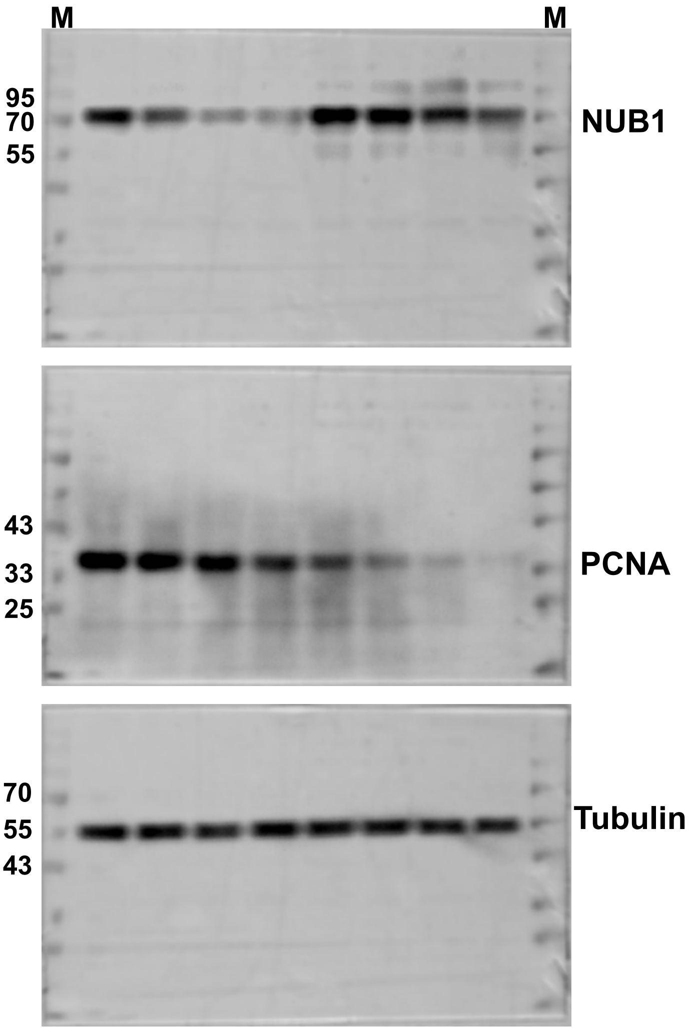

Fig3E-HCCLM3

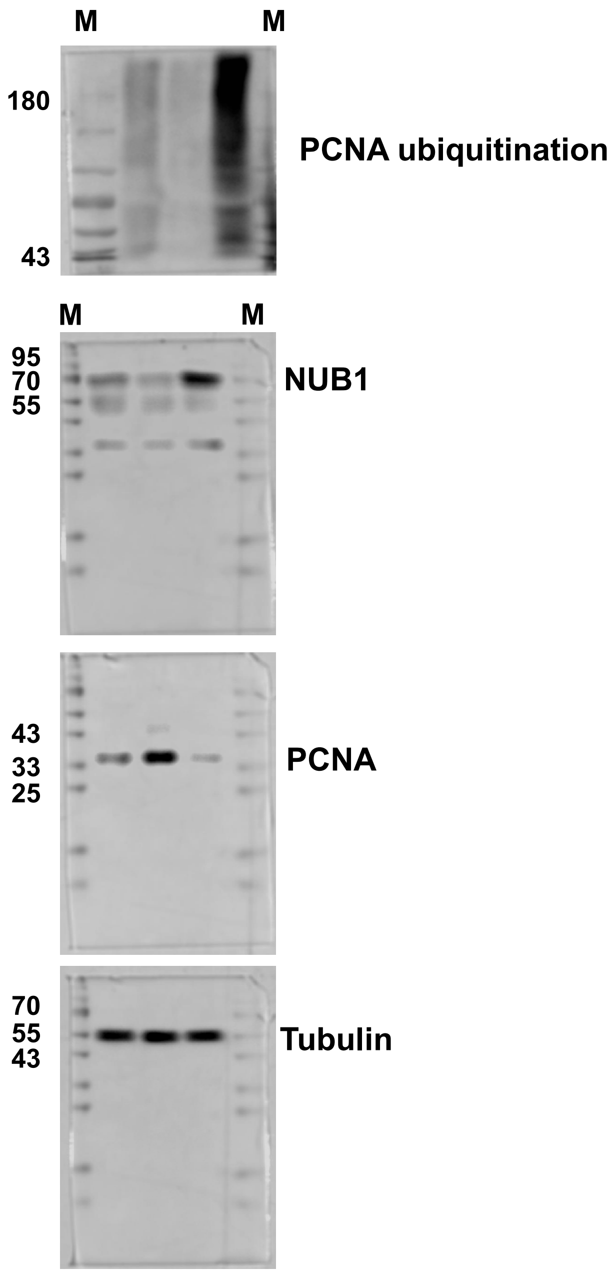

Fig3E-MHCC97H

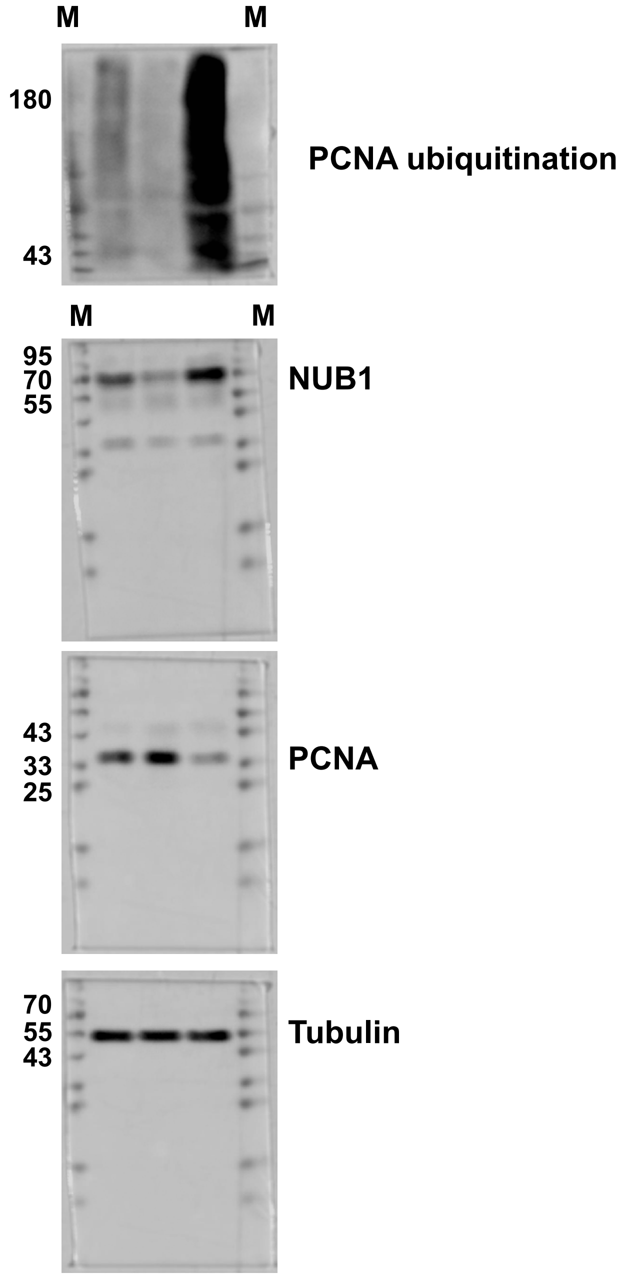

Fig4A-HCCLM3

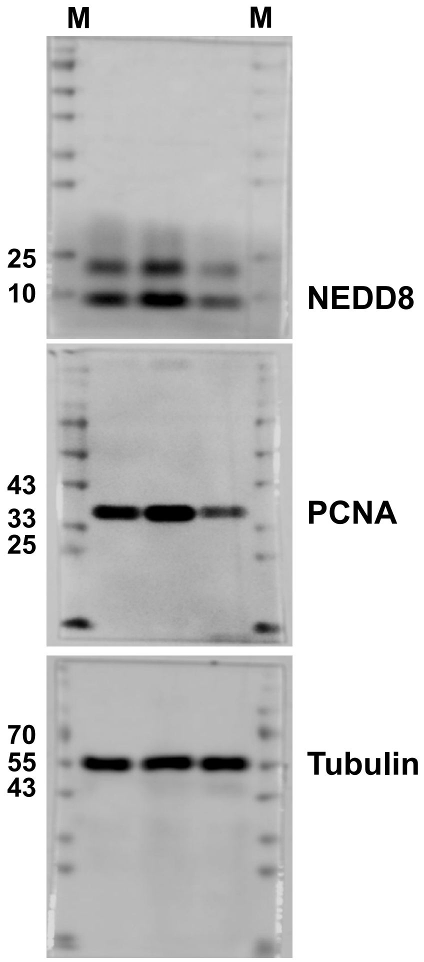

Fig4D-HCCLM3

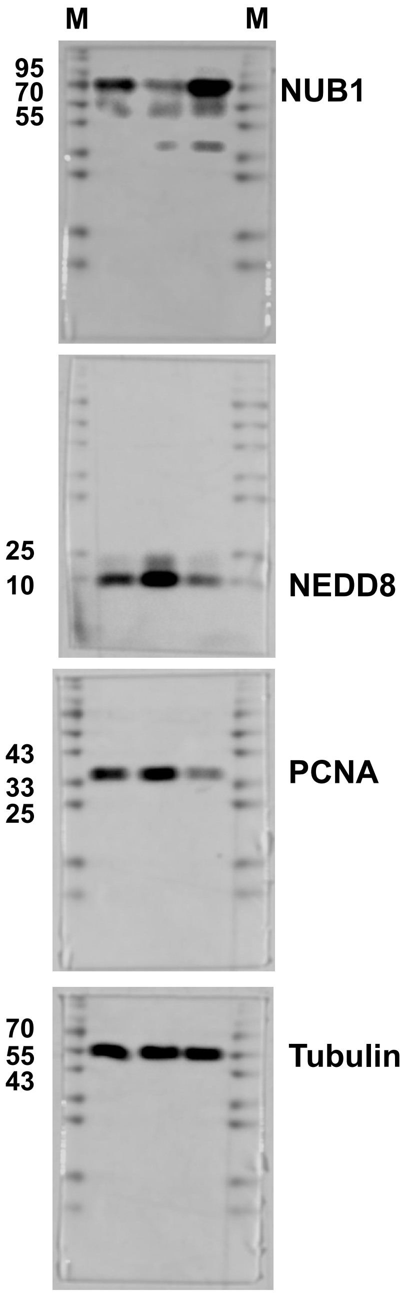

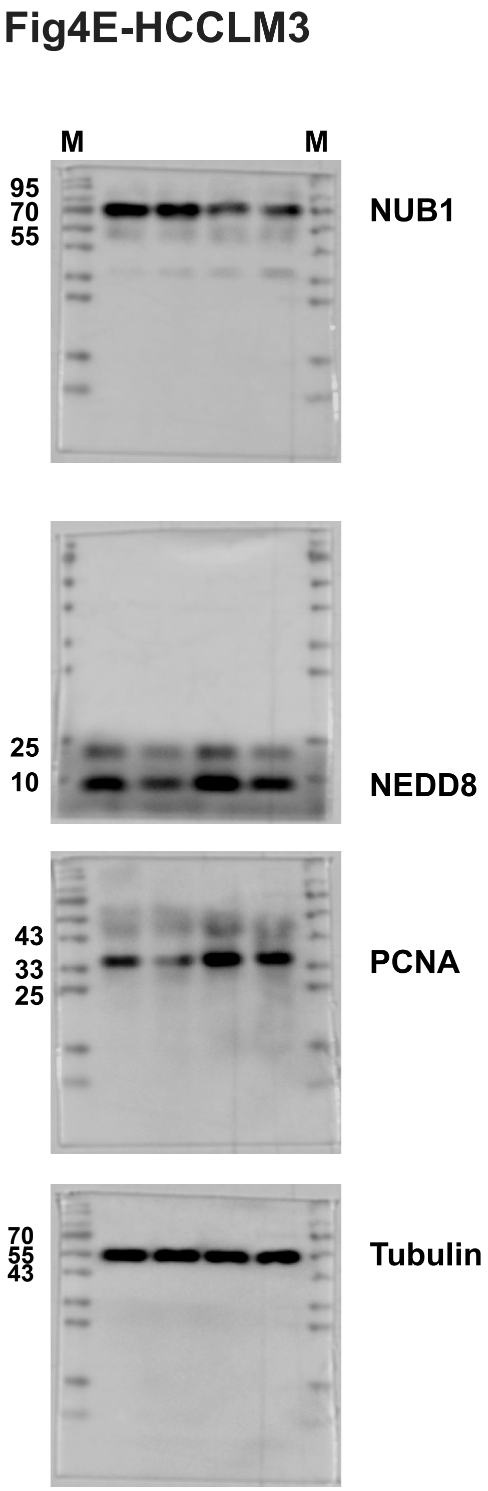
Fig4E-HCCLM3
M
M
95
70
55
NUB1
25
10
NEDD8
43
33
25
PCNA
70
55
43
Tubulin

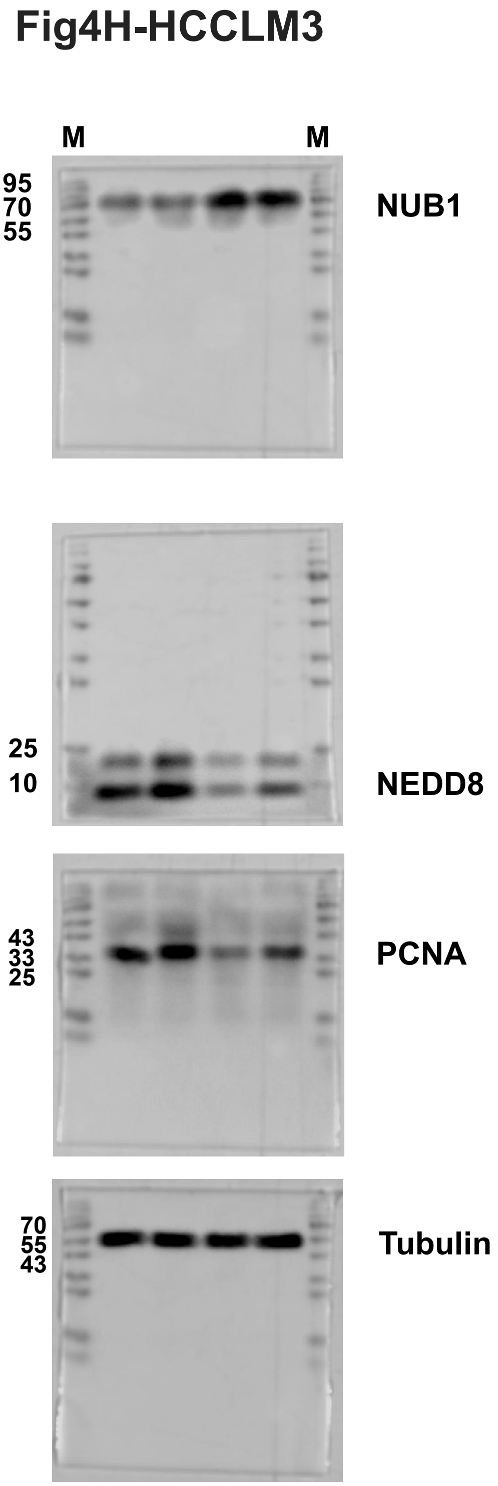
Fig4H-HCCLM3
M
M
95
70
55
NUB1
25
10
NEDD8
43
33
25
PCNA
70
55
43
Tubulin

Fig5A

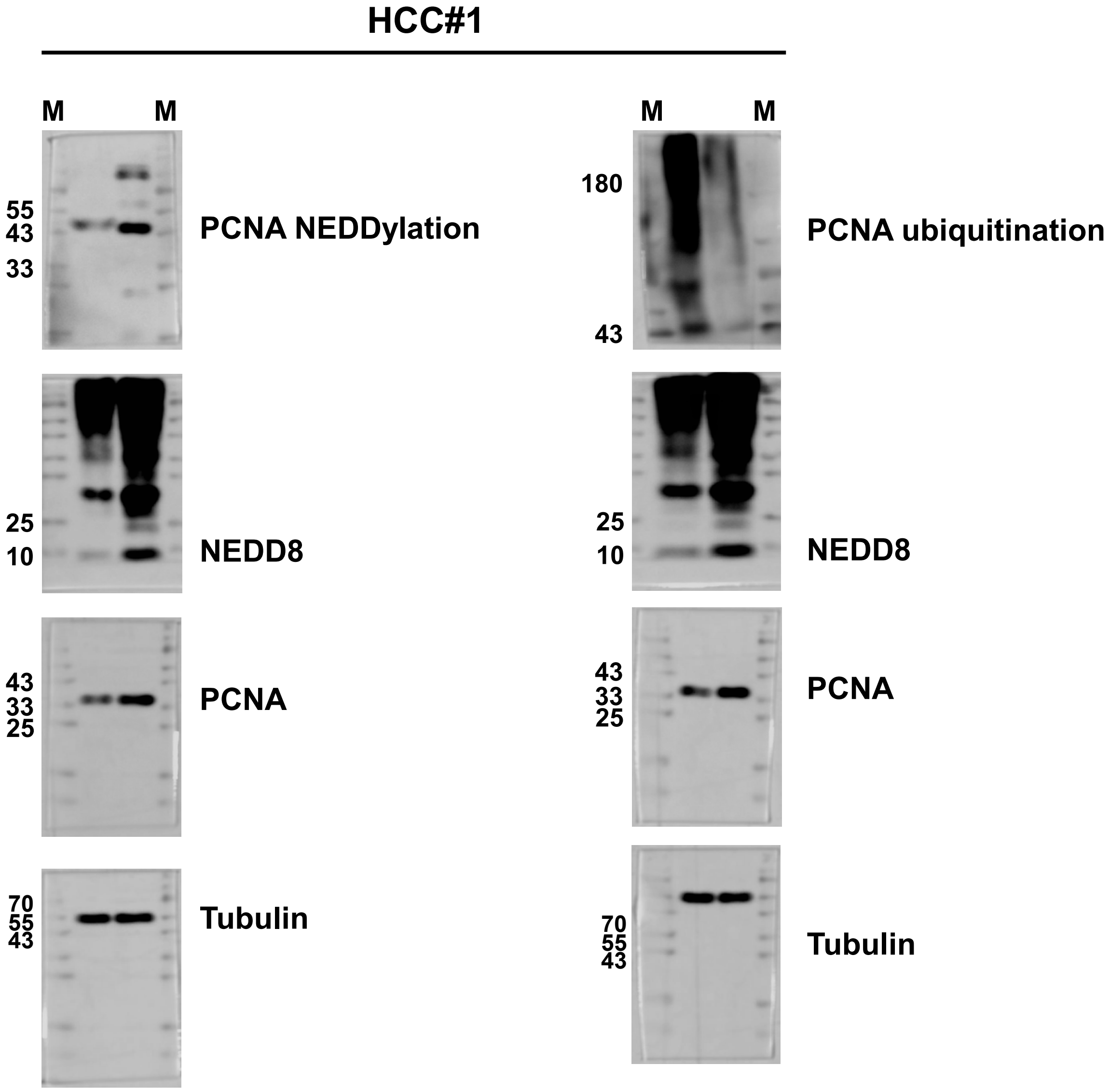
HCC#1
M
M
55
43
33
PCNA NEDDylation
M
M
180
43
PCNA ubiquitination
25
10
NEDD8
25
10
NEDD8
43
33
25
PCNA
43
33
25
PCNA
70
55
43
Tubulin
70
55
43
Tubulin

Fig5A

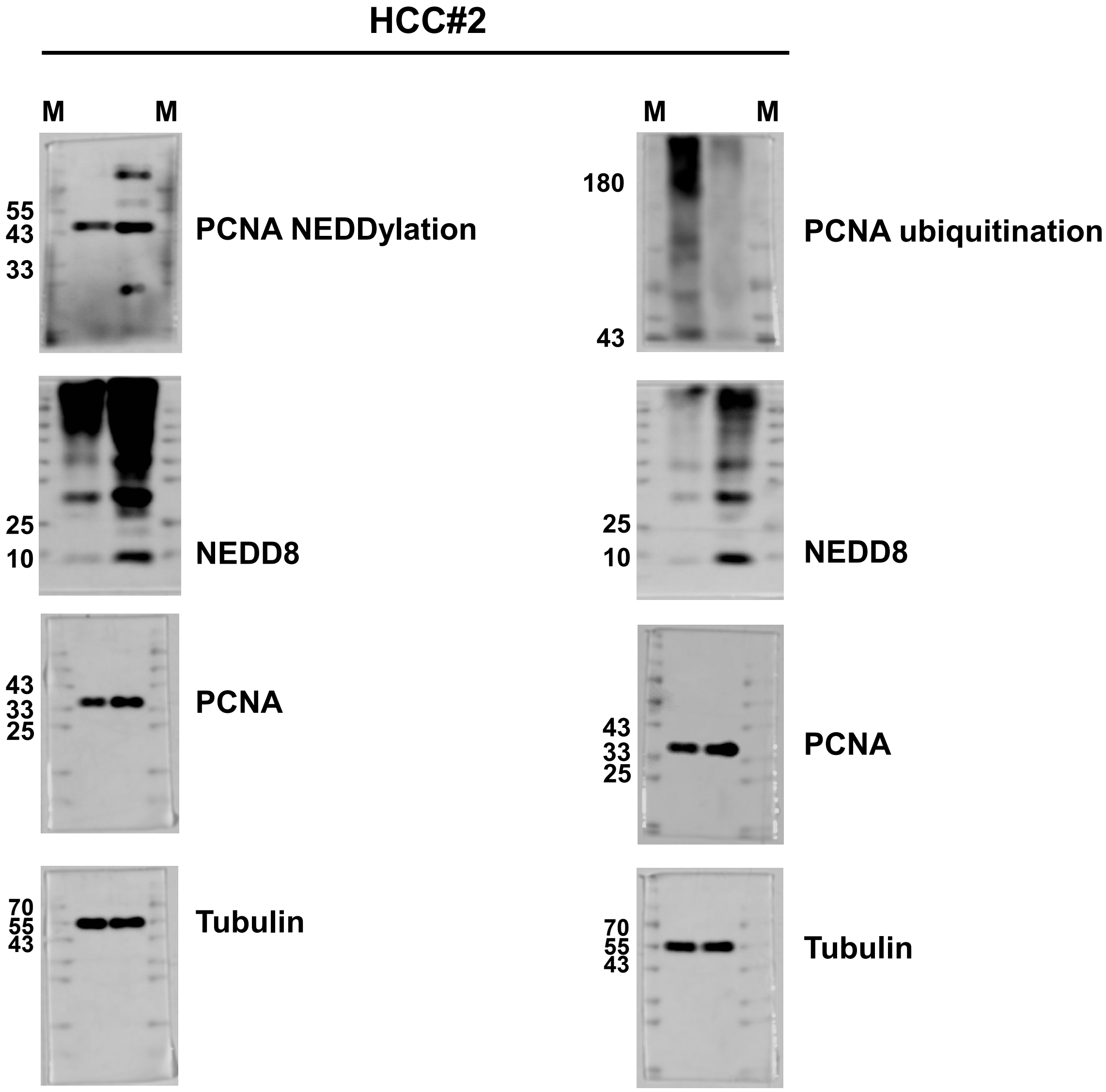
HCC#2
M
M
M
M
55
43
33
PCNA NEDDylation
180
43
PCNA ubiquitination
25
10
NEDD8
25
10
NEDD8
43
33
25
PCNA
43
33
25
PCNA
70
55
43
Tubulin
70
55
43
Tubulin

Fig5A

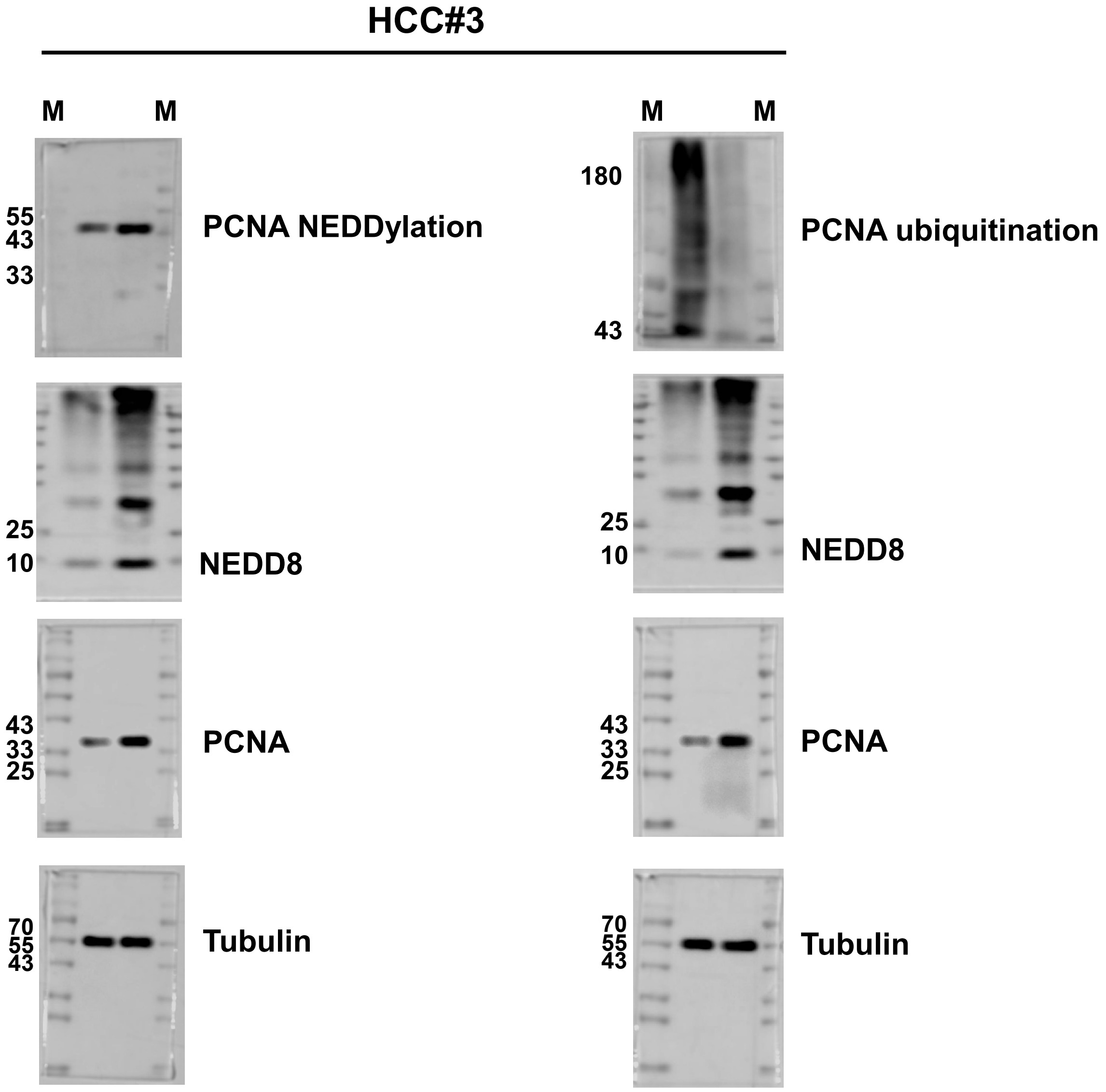
HCC#3
M
M
55
43
33
PCNA NEDDylation
25
10
NEDD8
43
33
25
PCNA
70
55
43
Tubulin
M
M
180
43
PCNA ubiquitination
25
10
NEDD8
43
33
25
PCNA
70
55
43
Tubulin

Fig5B

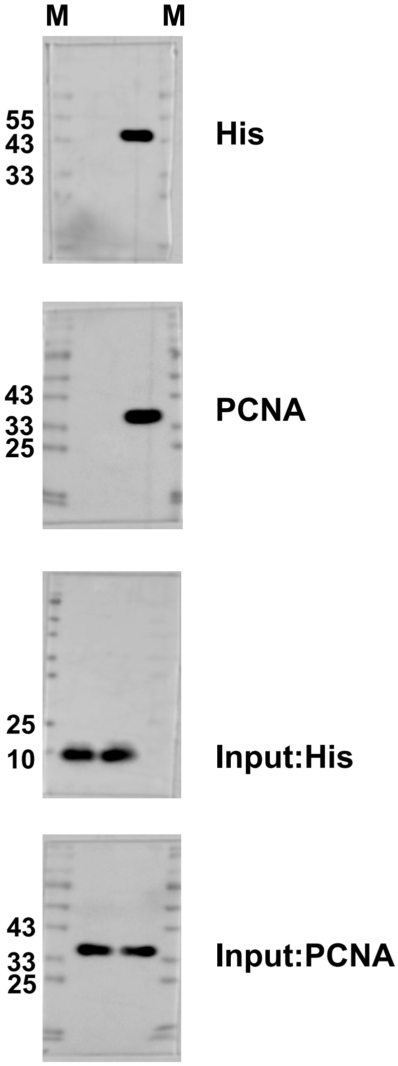
M
M
55
43
33
His
43
33
25
PCNA
25
10
Input:His
43
33
25
Input:PCNA

Fig5D

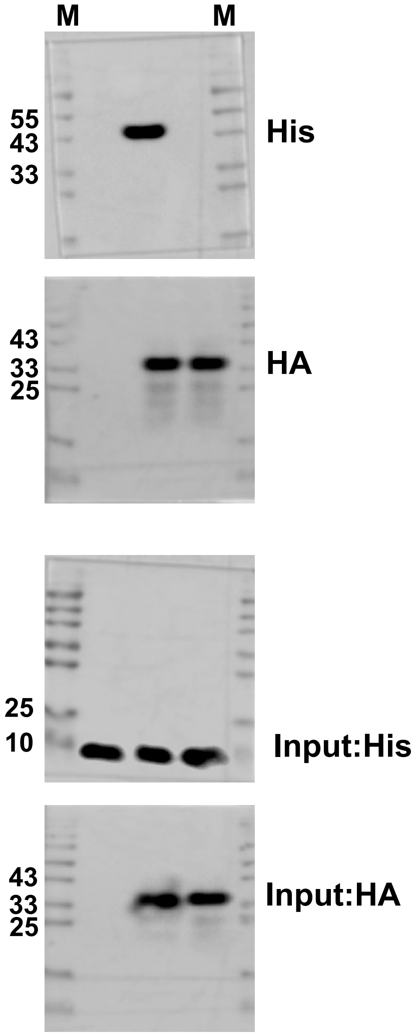
M
M
55
43
33
His
43
33
25
HA
25
10
Input:His
43
33
25
Input:HA

Fig5E

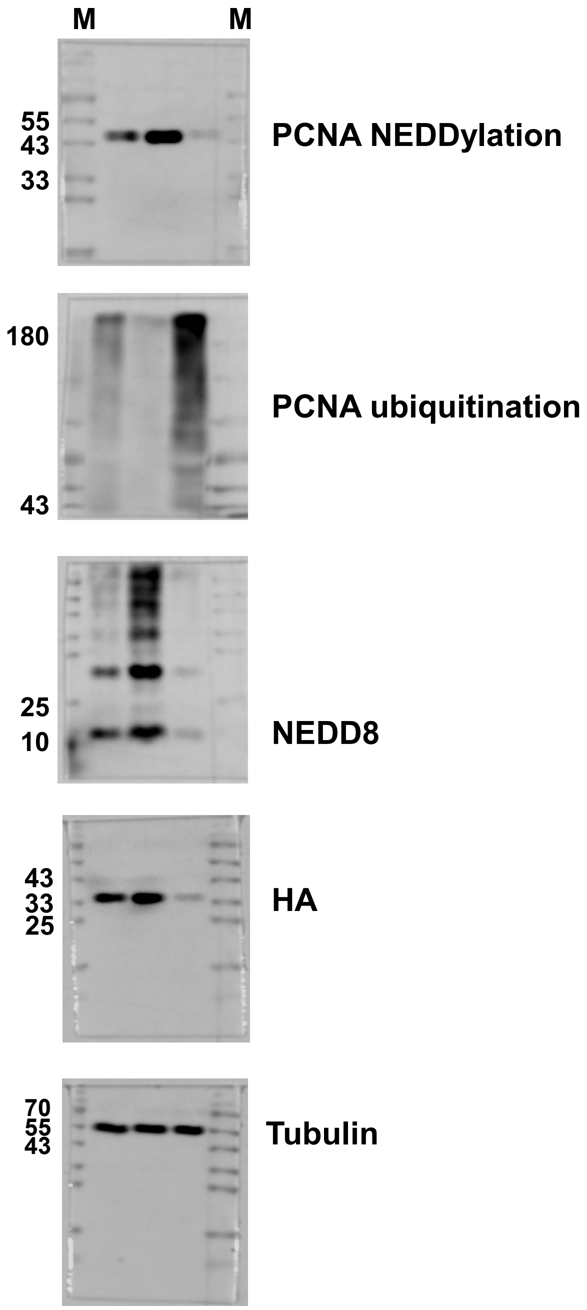
M
M
55
43
33
PCNA NEDDylation
180
43
PCNA ubiquitination
25
10
NEDD8
43
33
25
HA
70
55
43
Tubulin

Fig5F

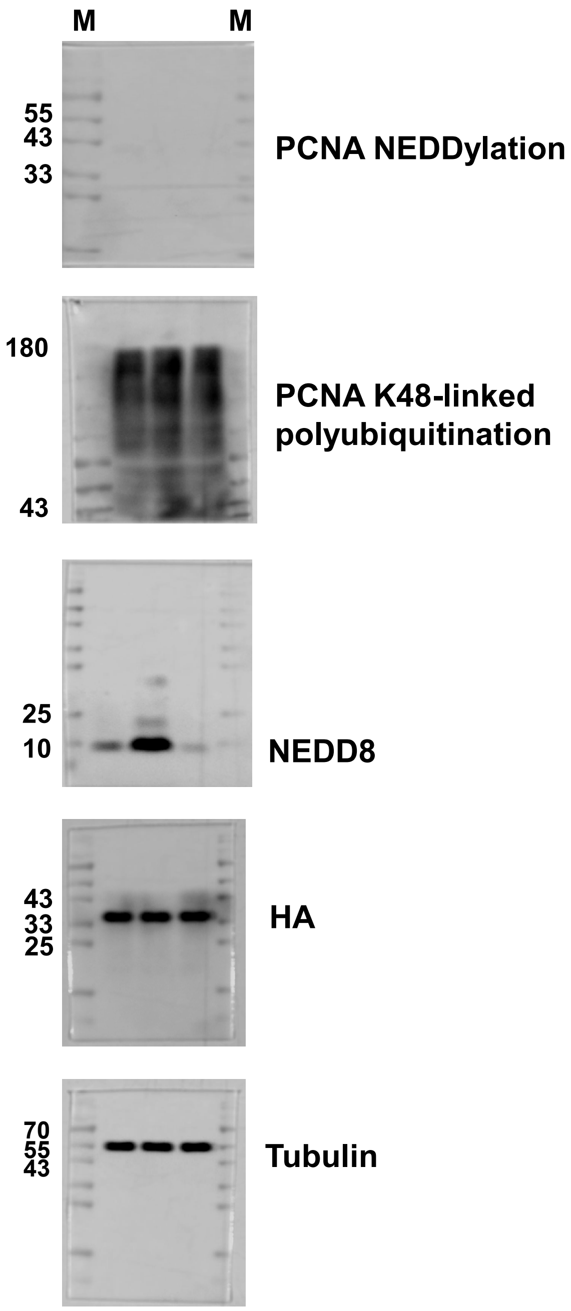
M
M
55
43
33
PCNA NEDDylation
180
43
PCNA K48-linked polyubiquitination
25
10
NEDD8
43
33
25
HA
70
55
43
Tubulin

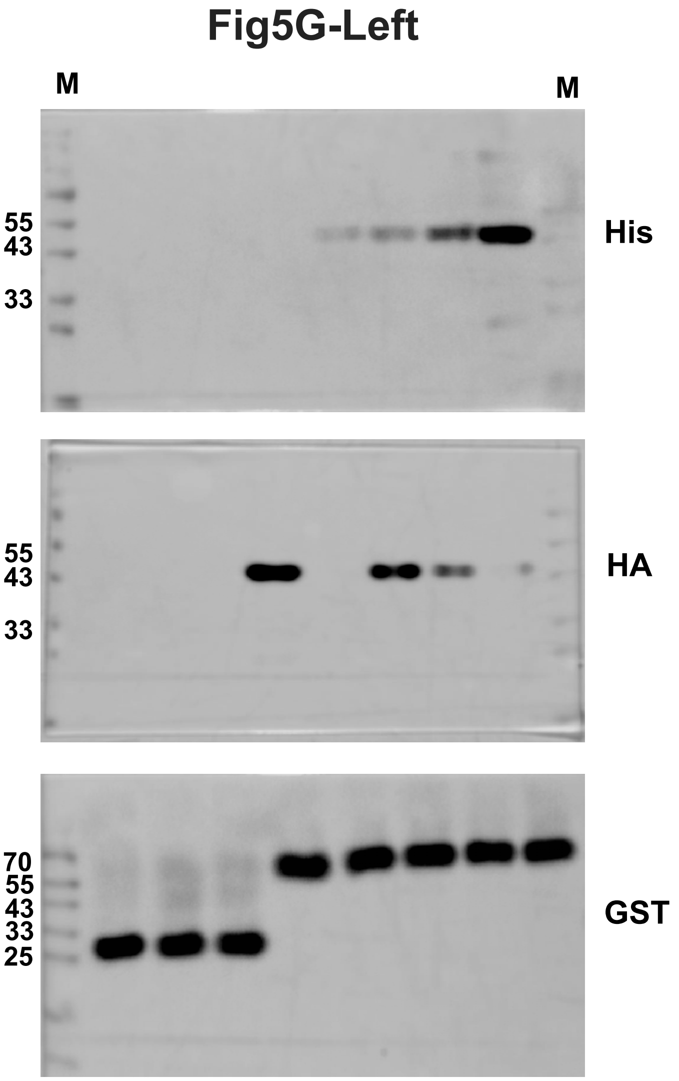
Fig5G-Left
M
M
55
43
33
His
55
43
33
HA
70
55
43
33
25
GST

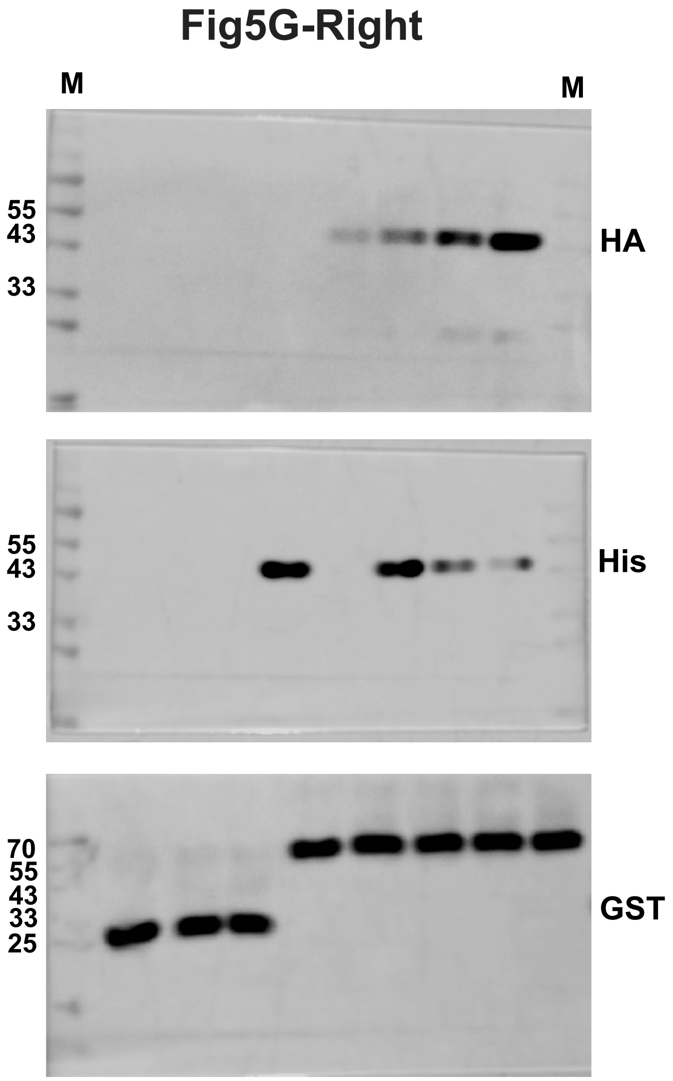
Fig5G-Right
M
M
55
43
33
HA
55
43
33
His
70
55
43
33
25
GST

Fig5H-Left

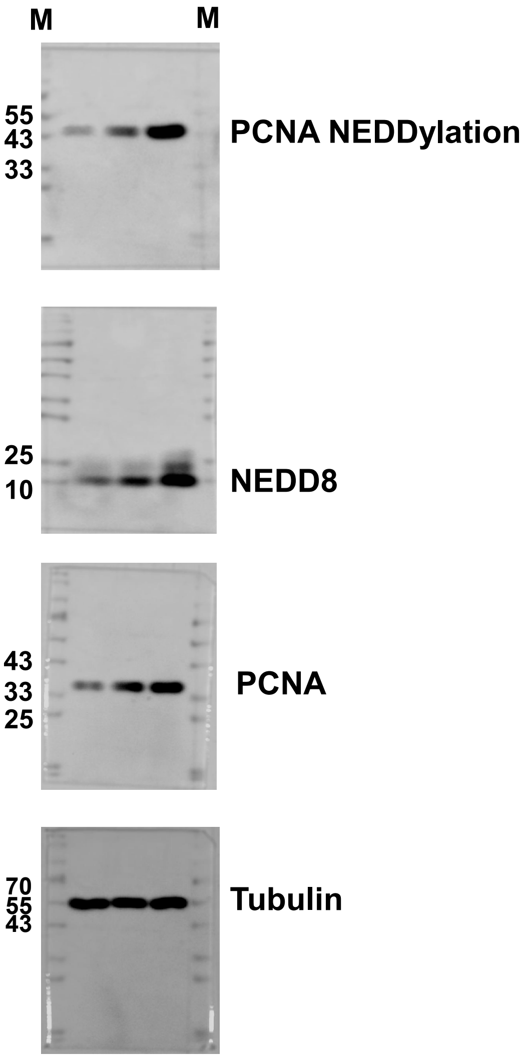

Fig5H-Right

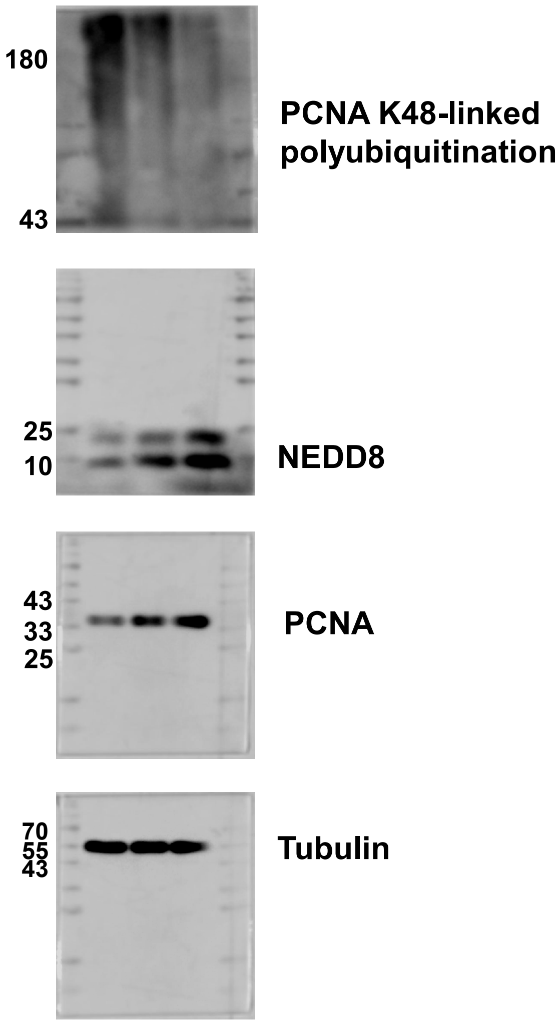

Fig5I-Left

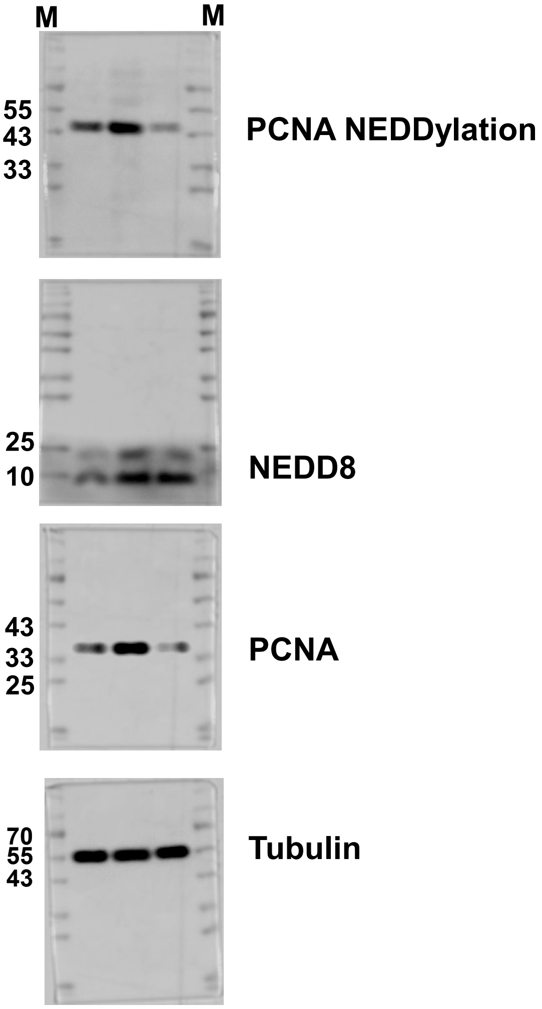

Fig5I-Right

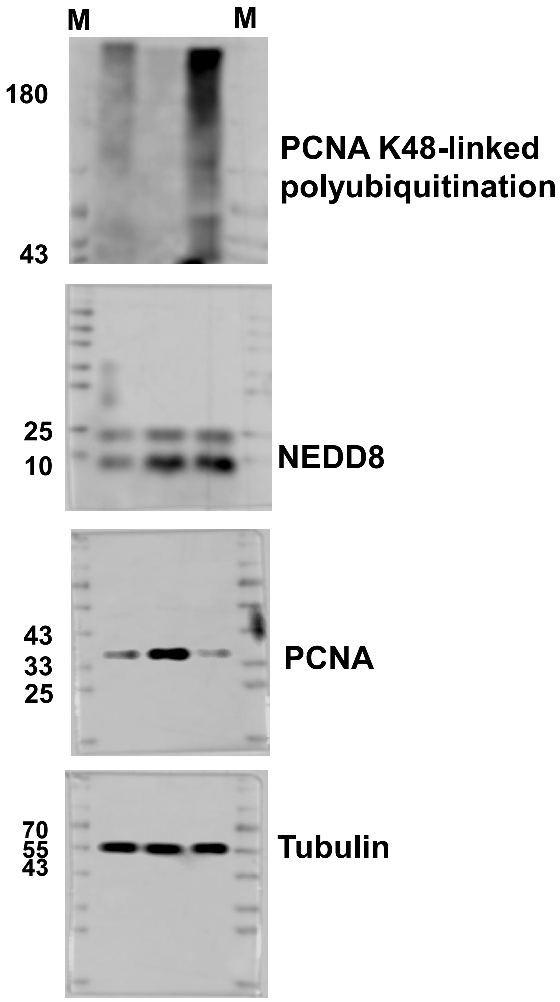

Fig6A

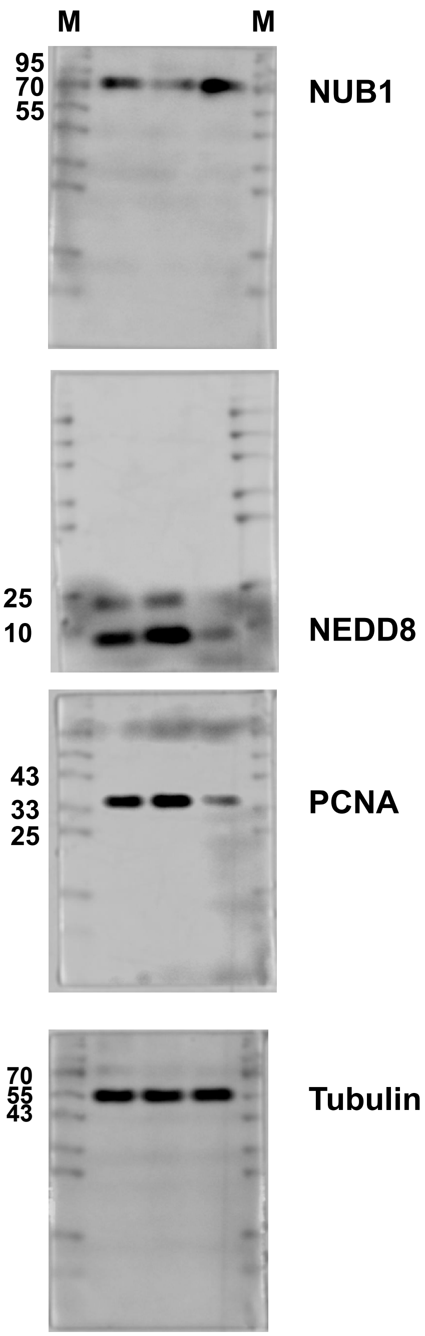

Fig6B-Left

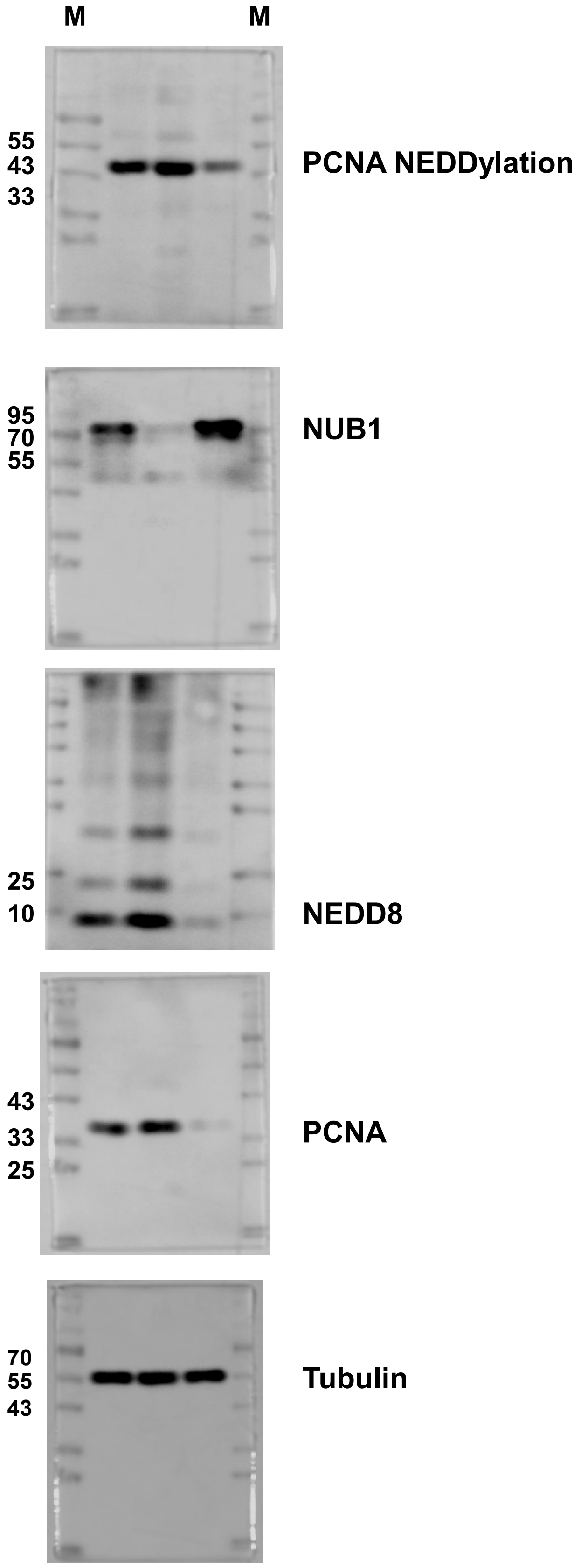

Fig6B-Right

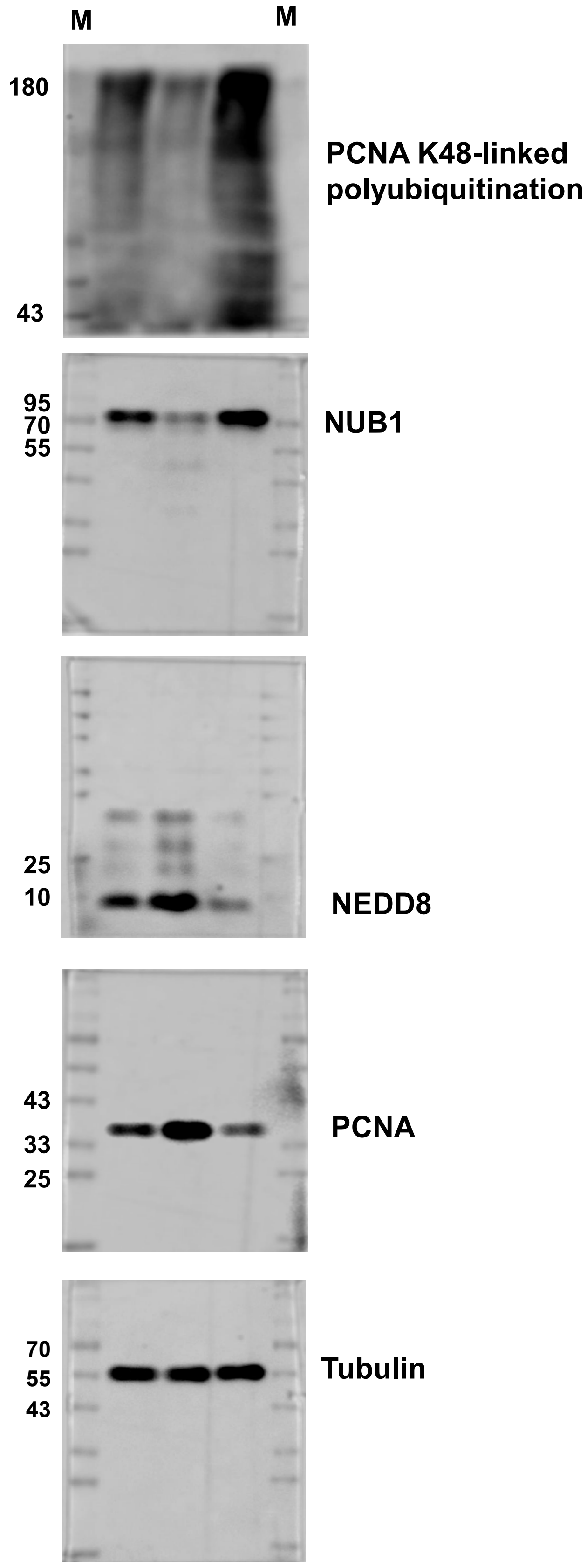

Fig6C-Left

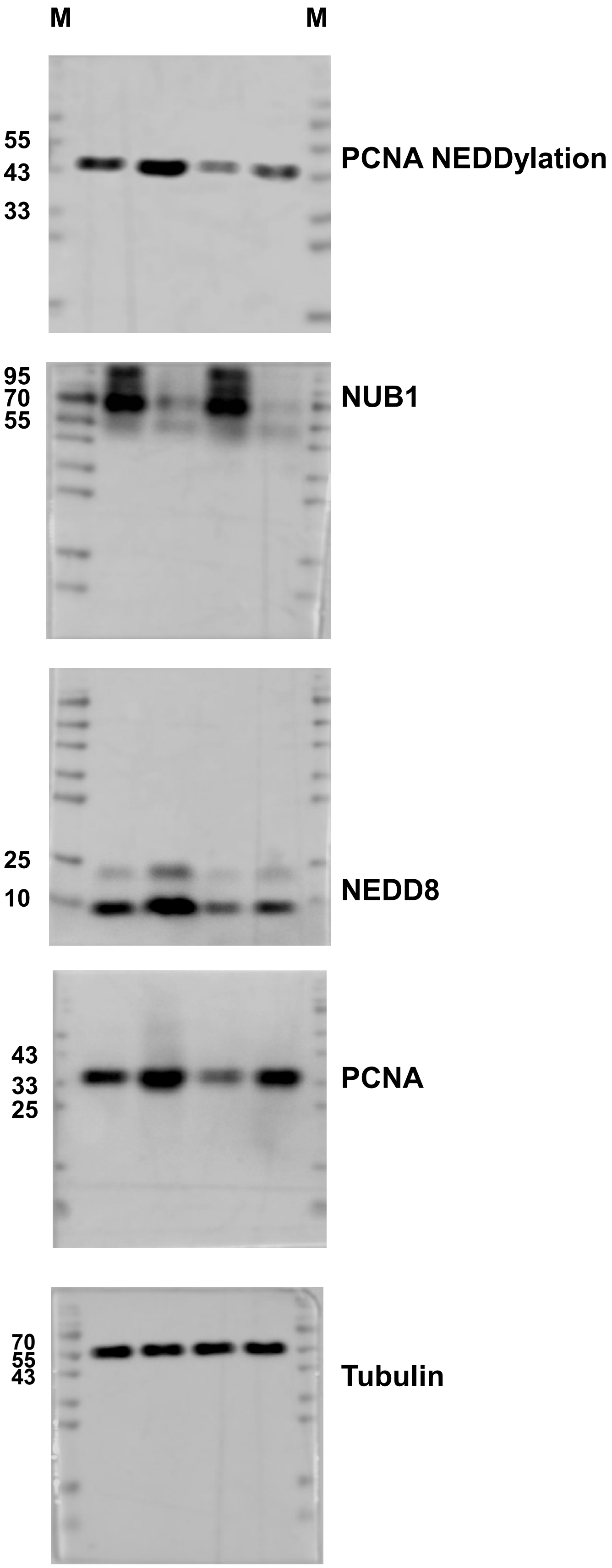

Fig6C-Right

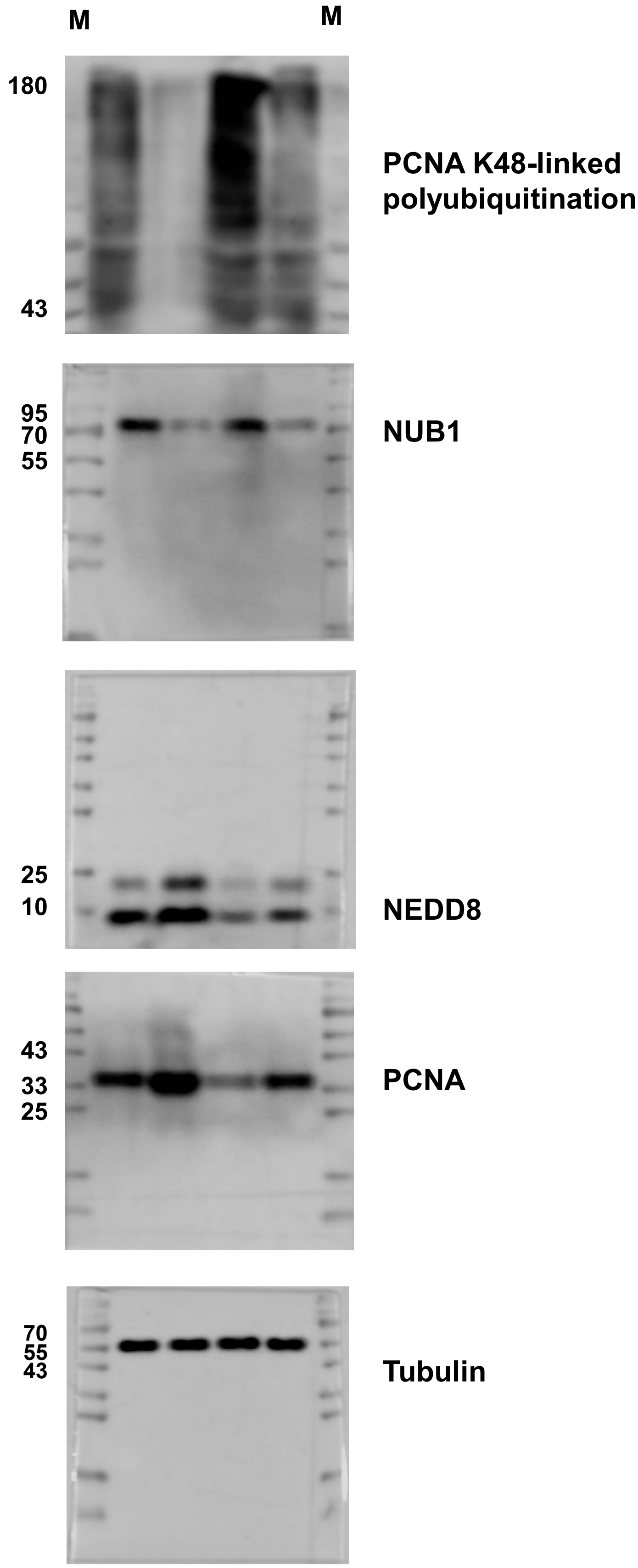

Fig6D-Left

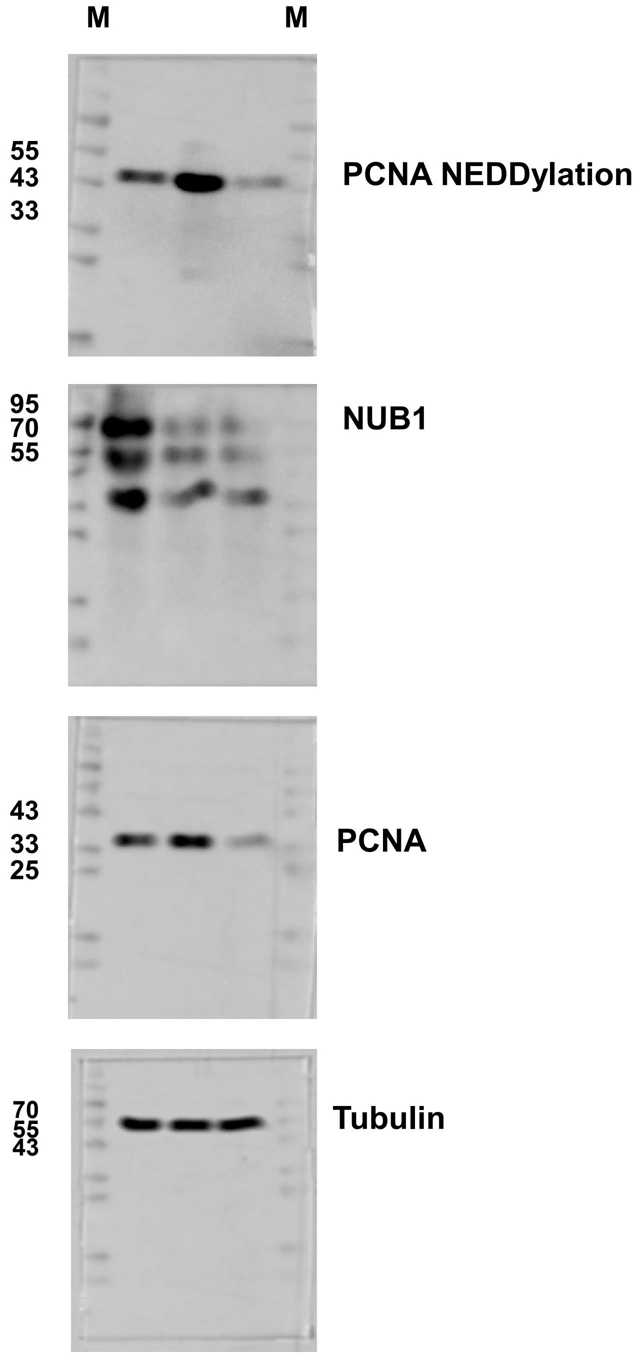

Fig6D-Right

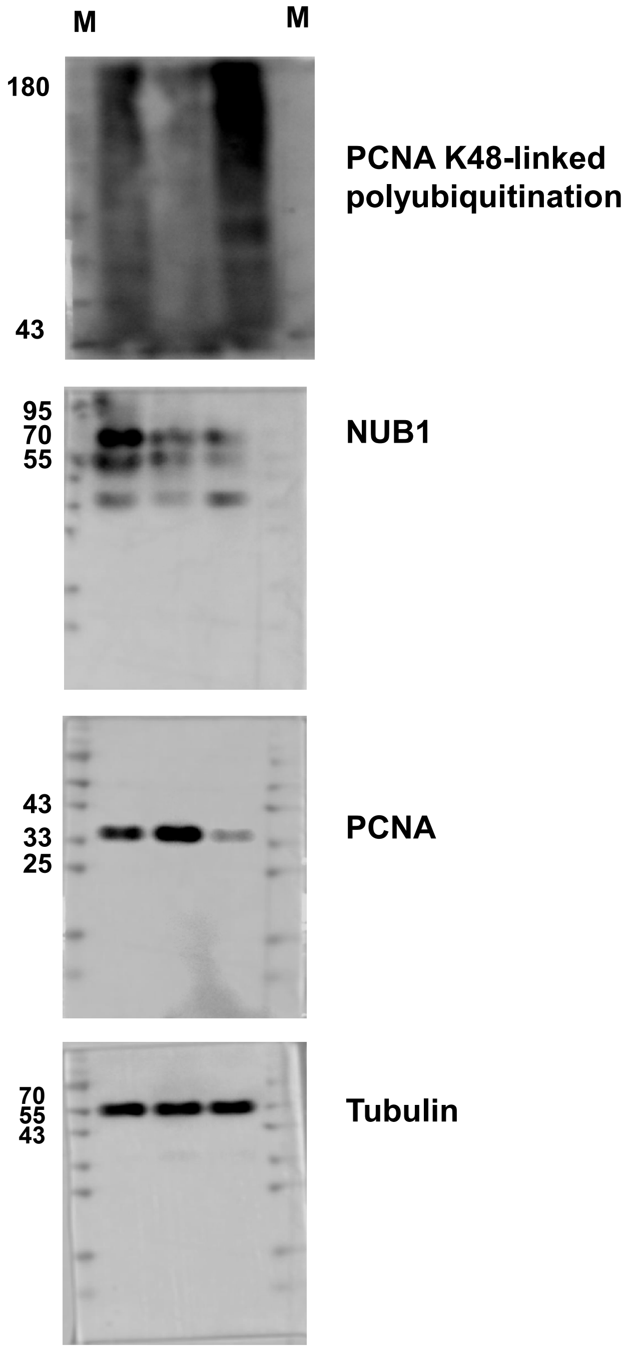

Fig6E-Left

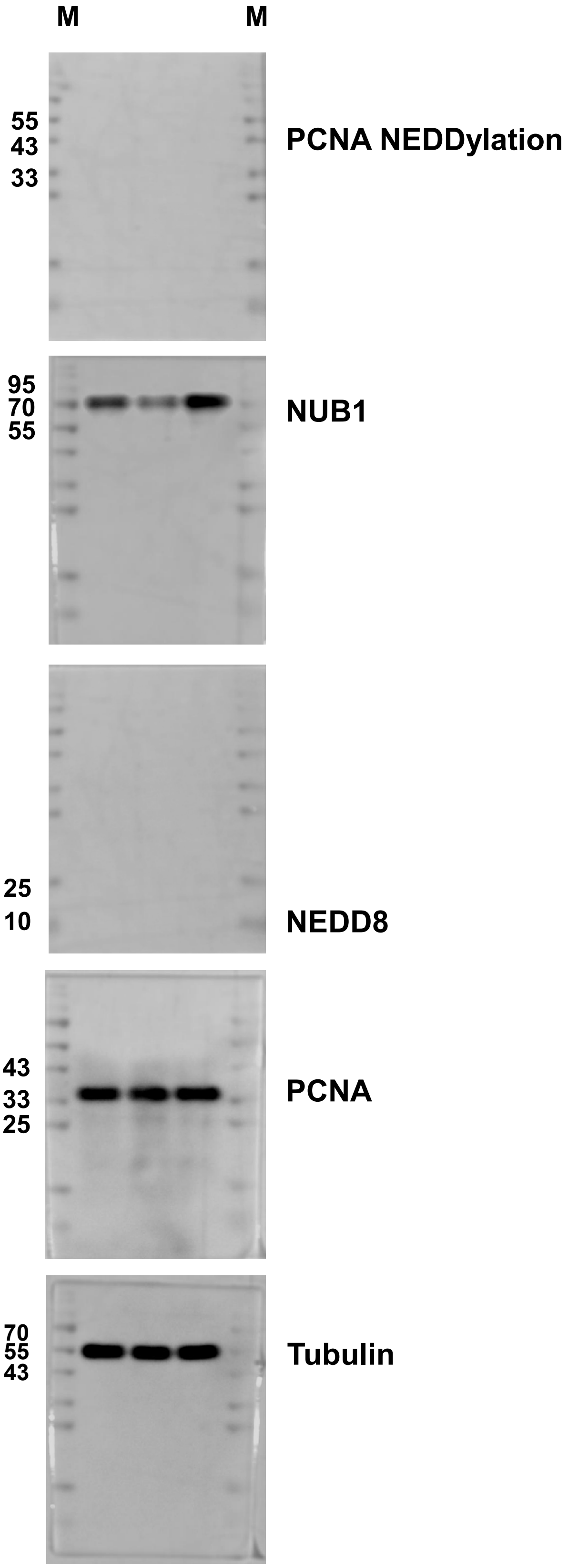

Fig6E-Right

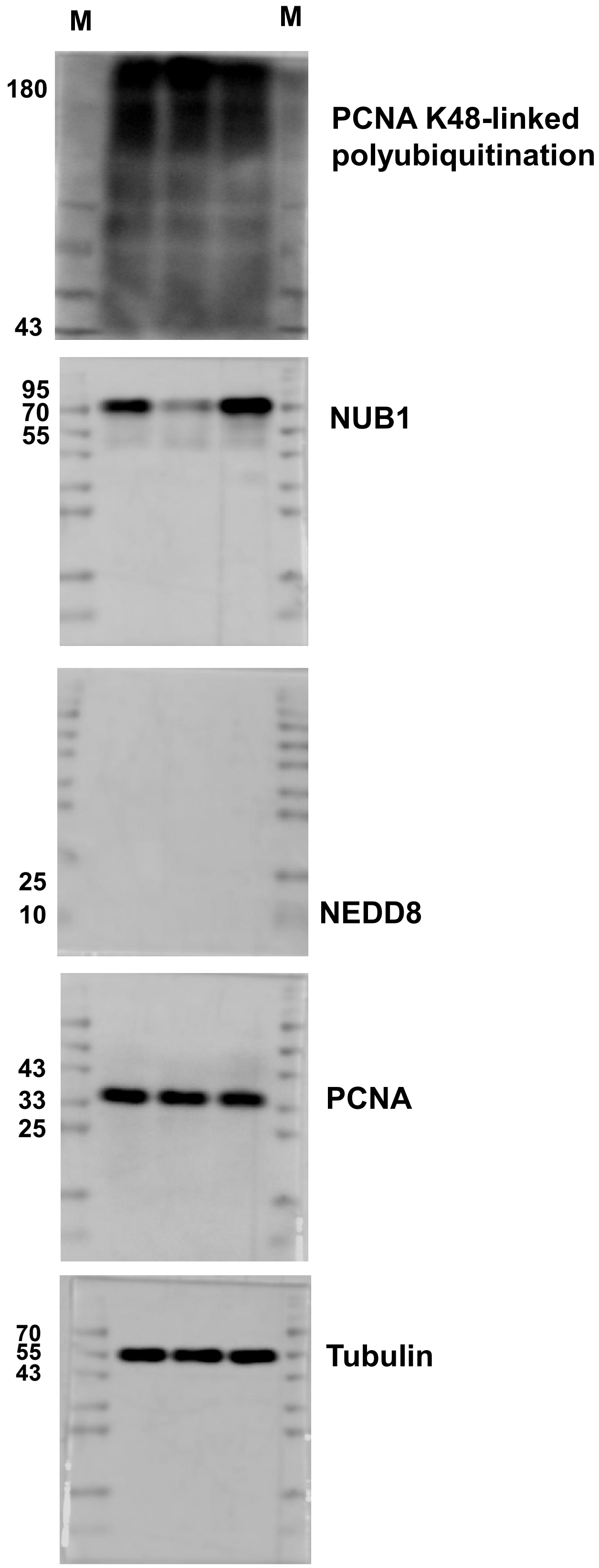

Fig6F-Left

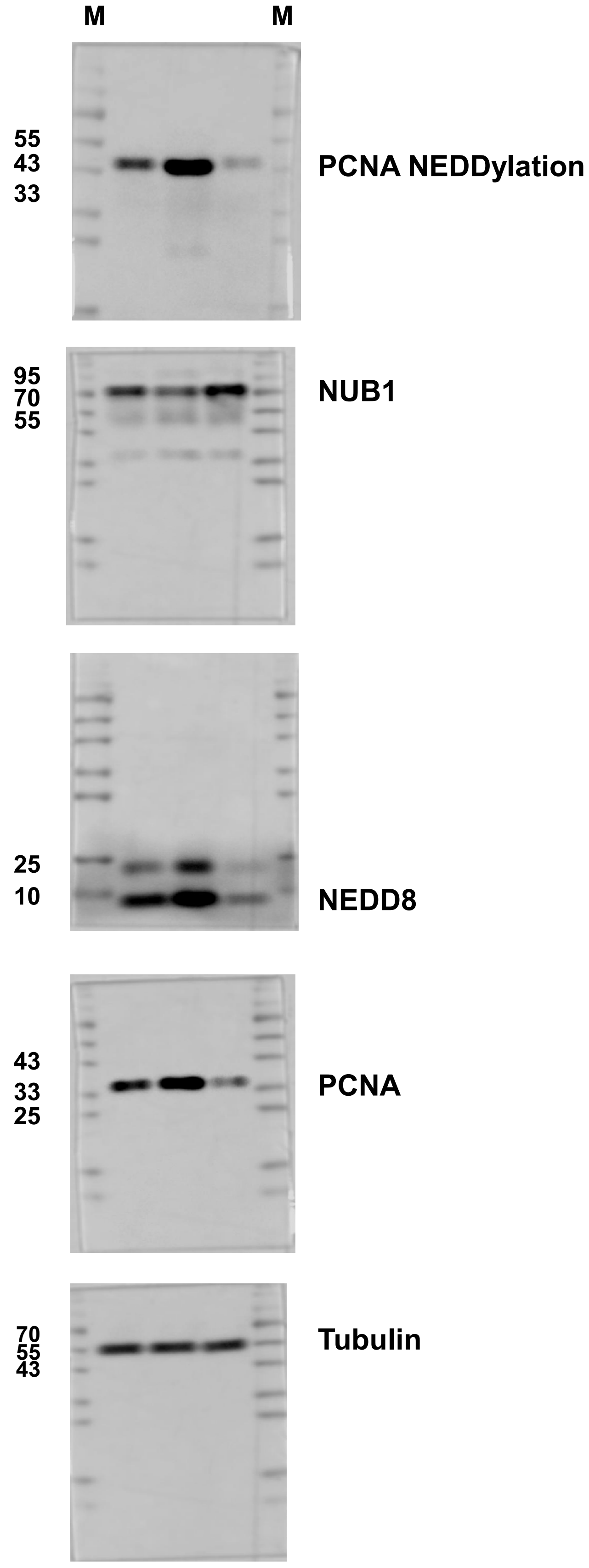

Fig6F-Right

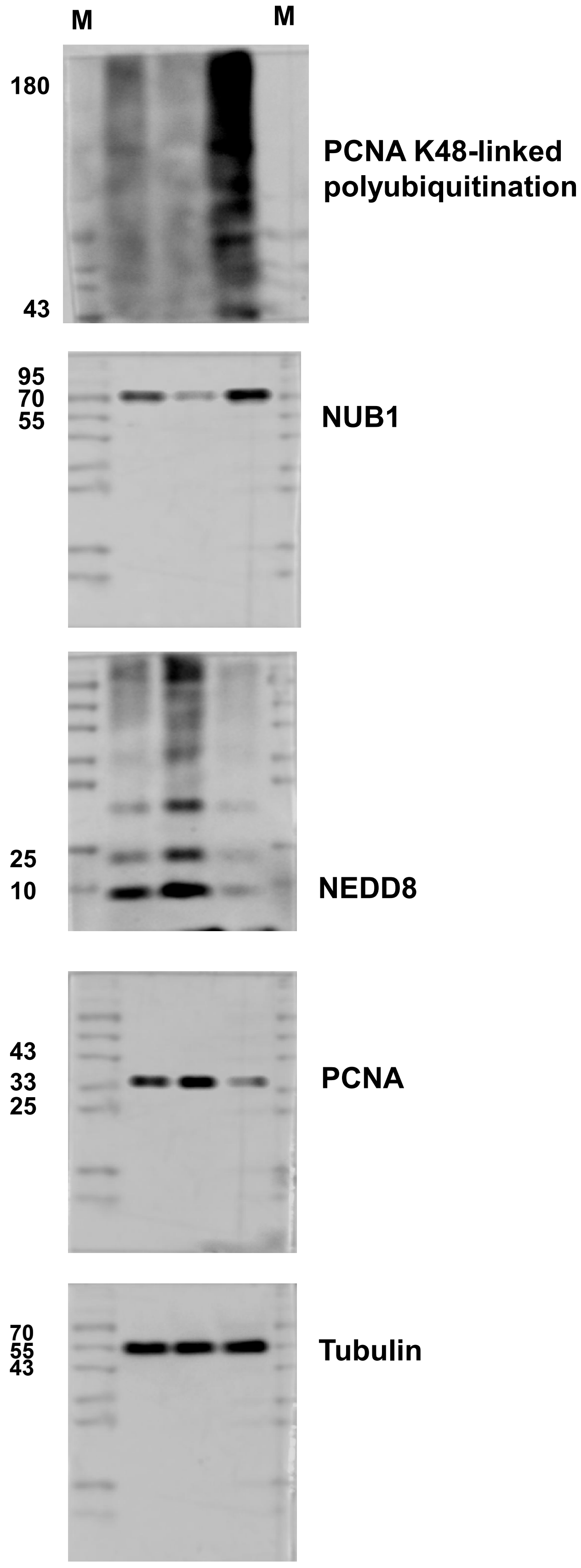

Fig7F-Left

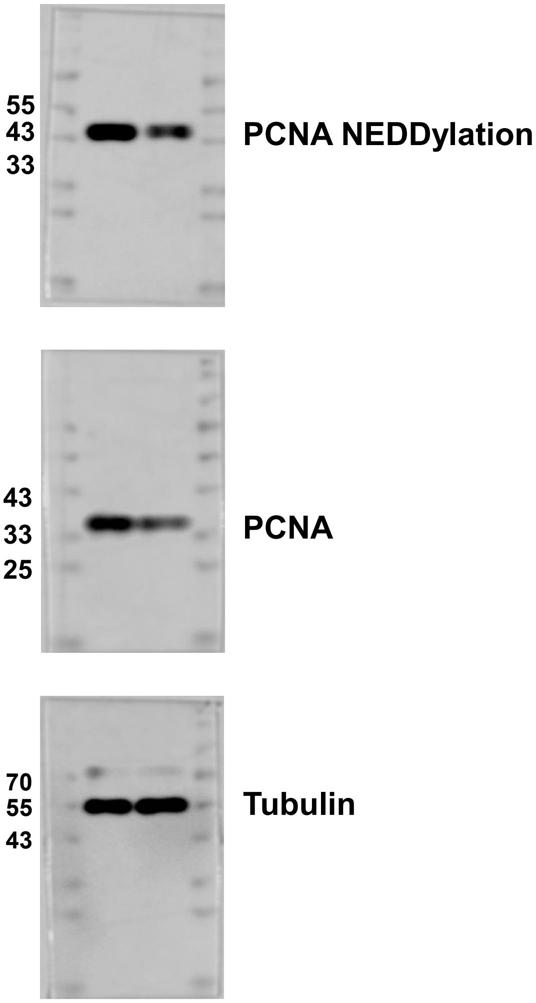

Fig7F-Right

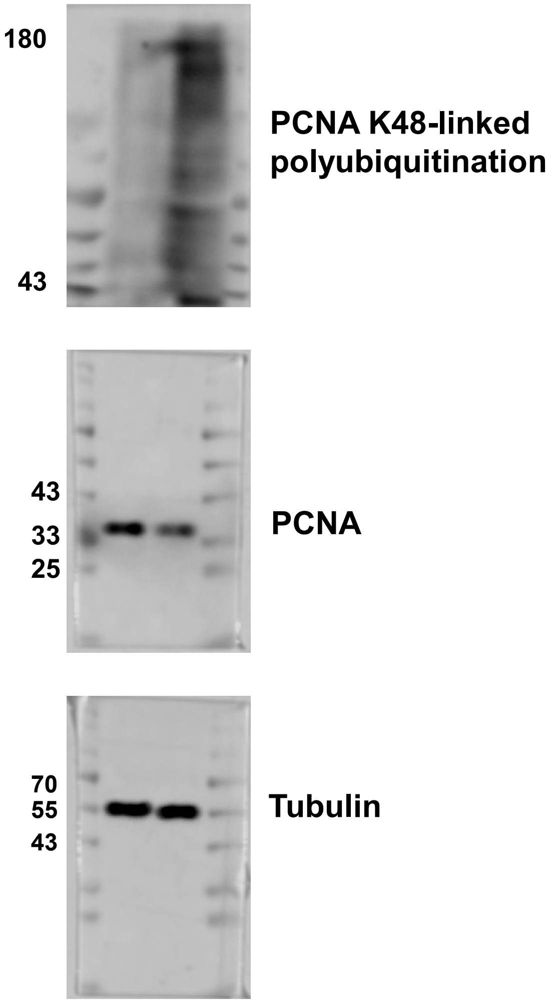

Supplementary Figure 1

FigS1A

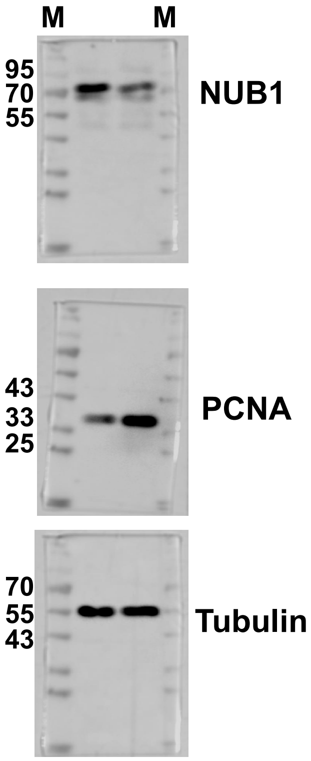

FigS1D

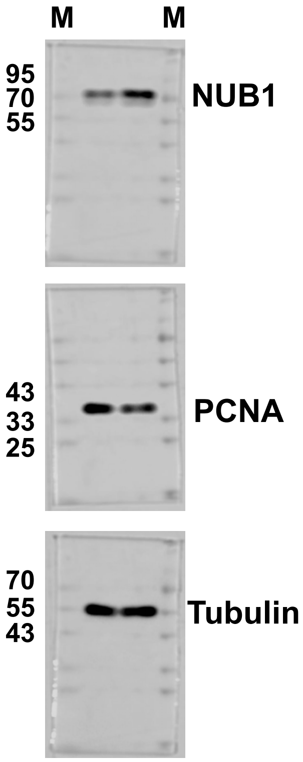

FigS1G

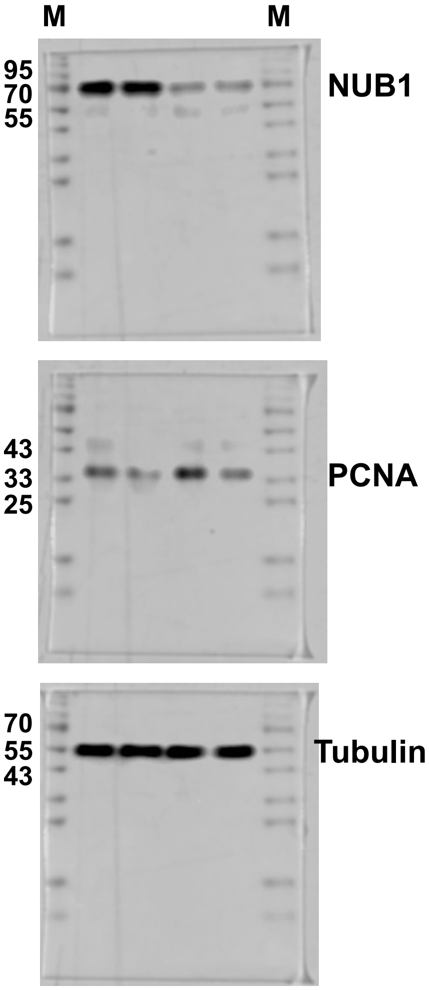

Supplementary Figure 2

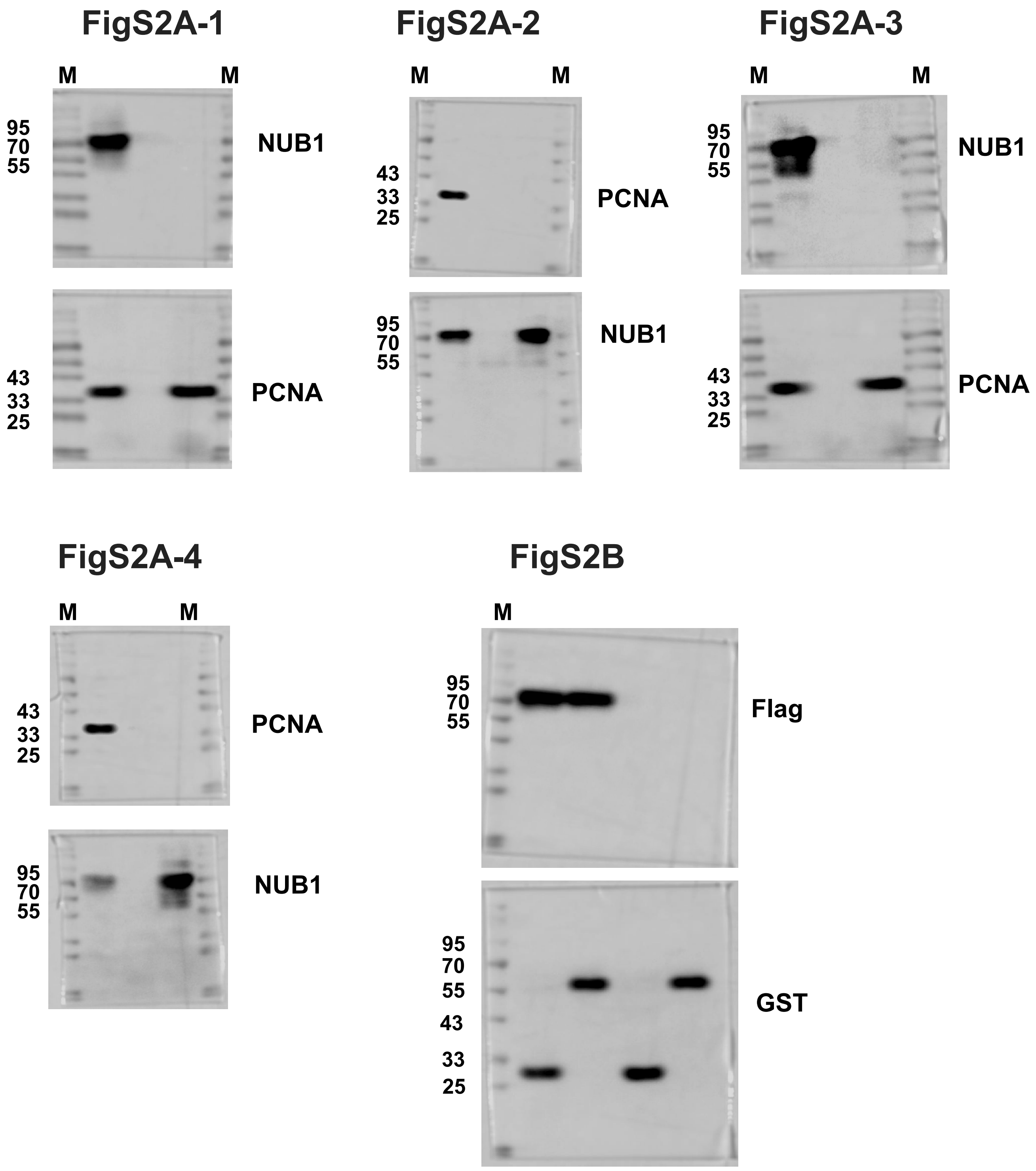

Supplementary Figure 2

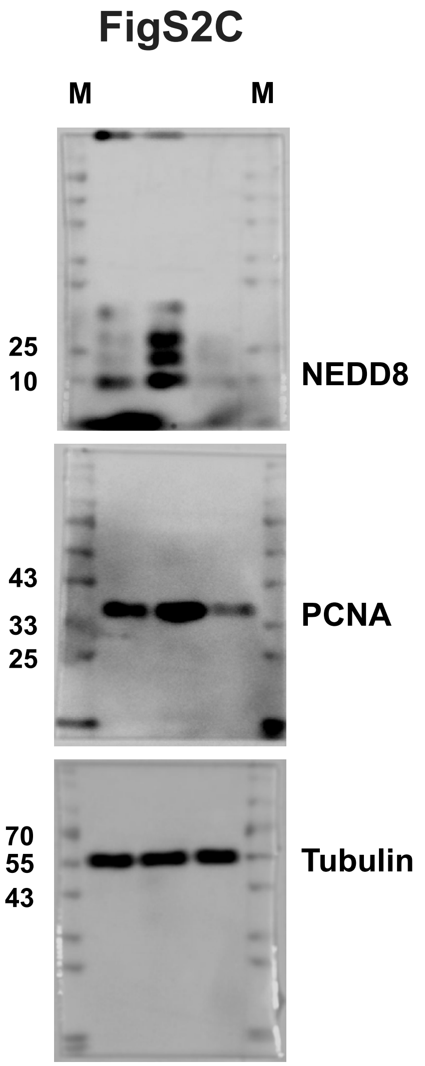

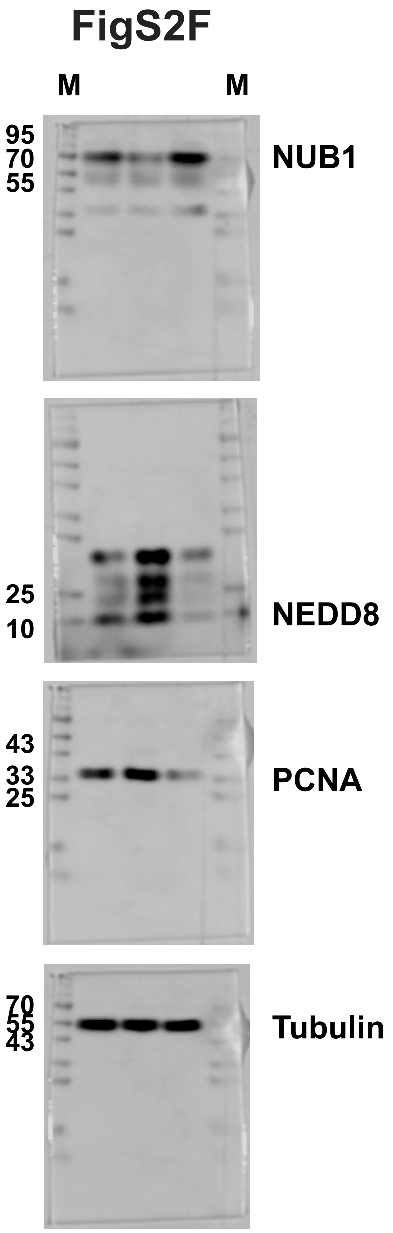

Supplementary Figure 2

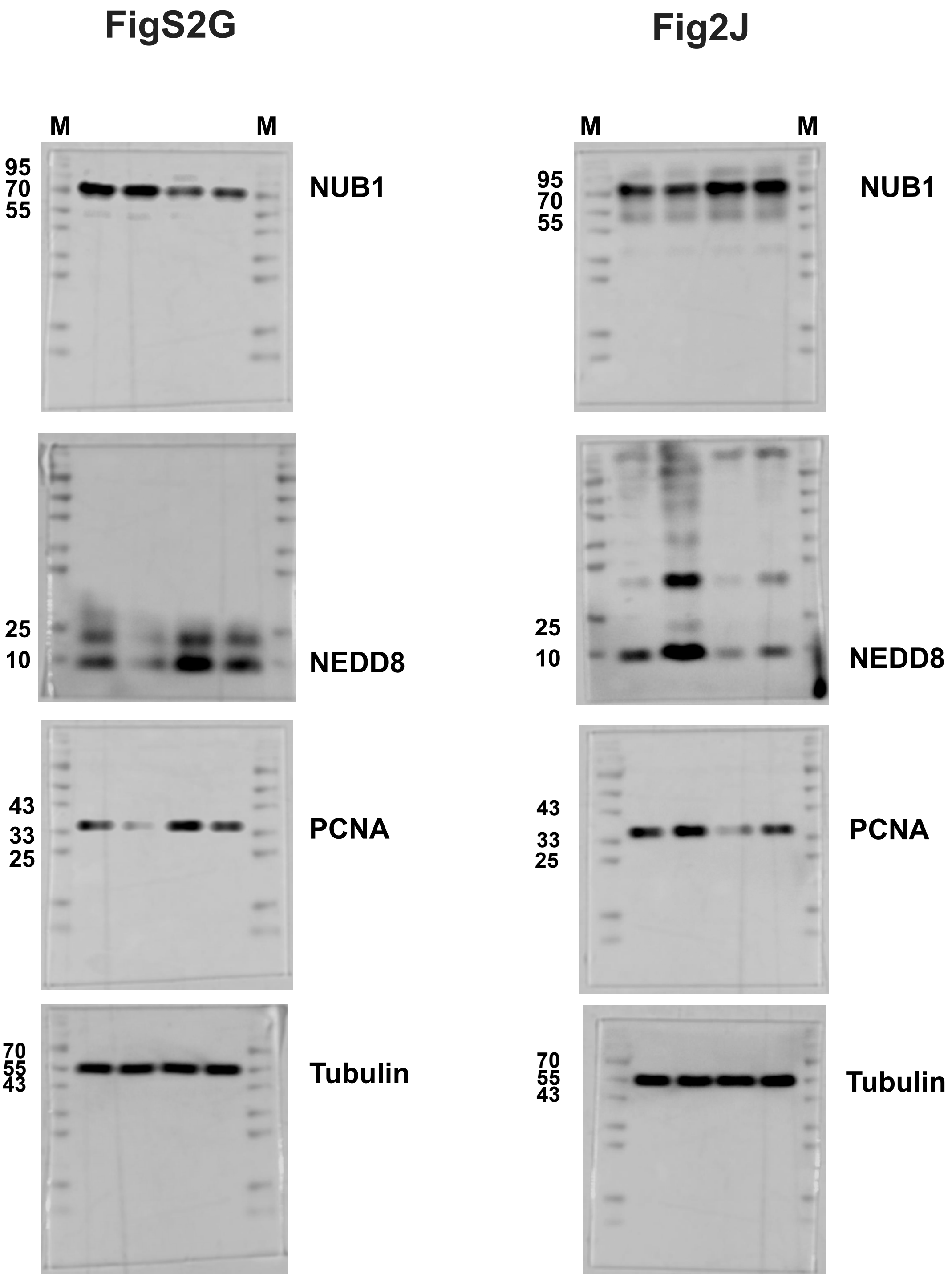

Supplementary Figure 3

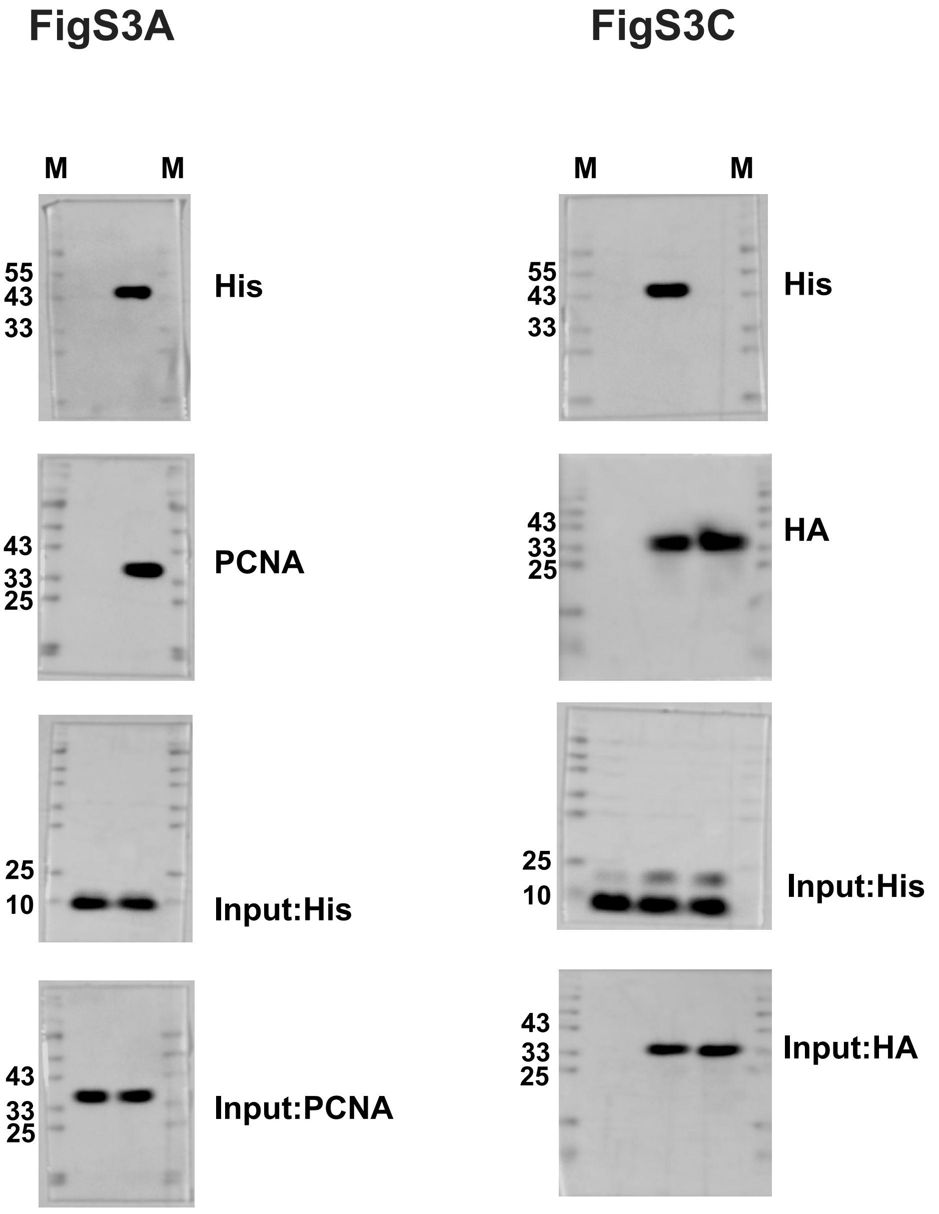

Supplementary Figure 3

FigS3D

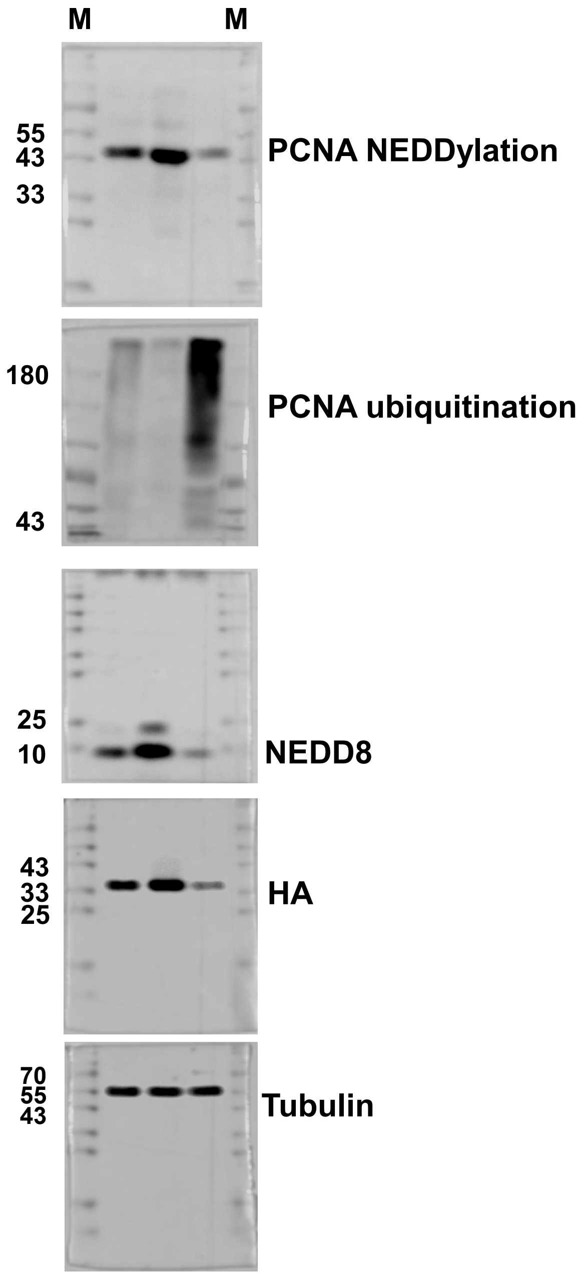

FigS3E

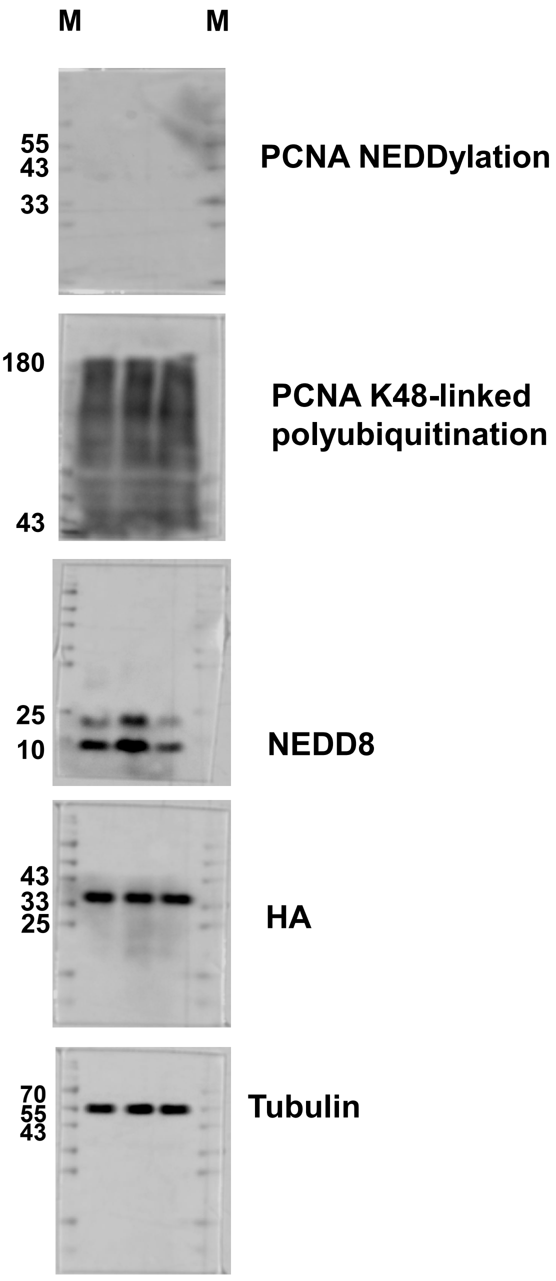

Supplementary Figure 3

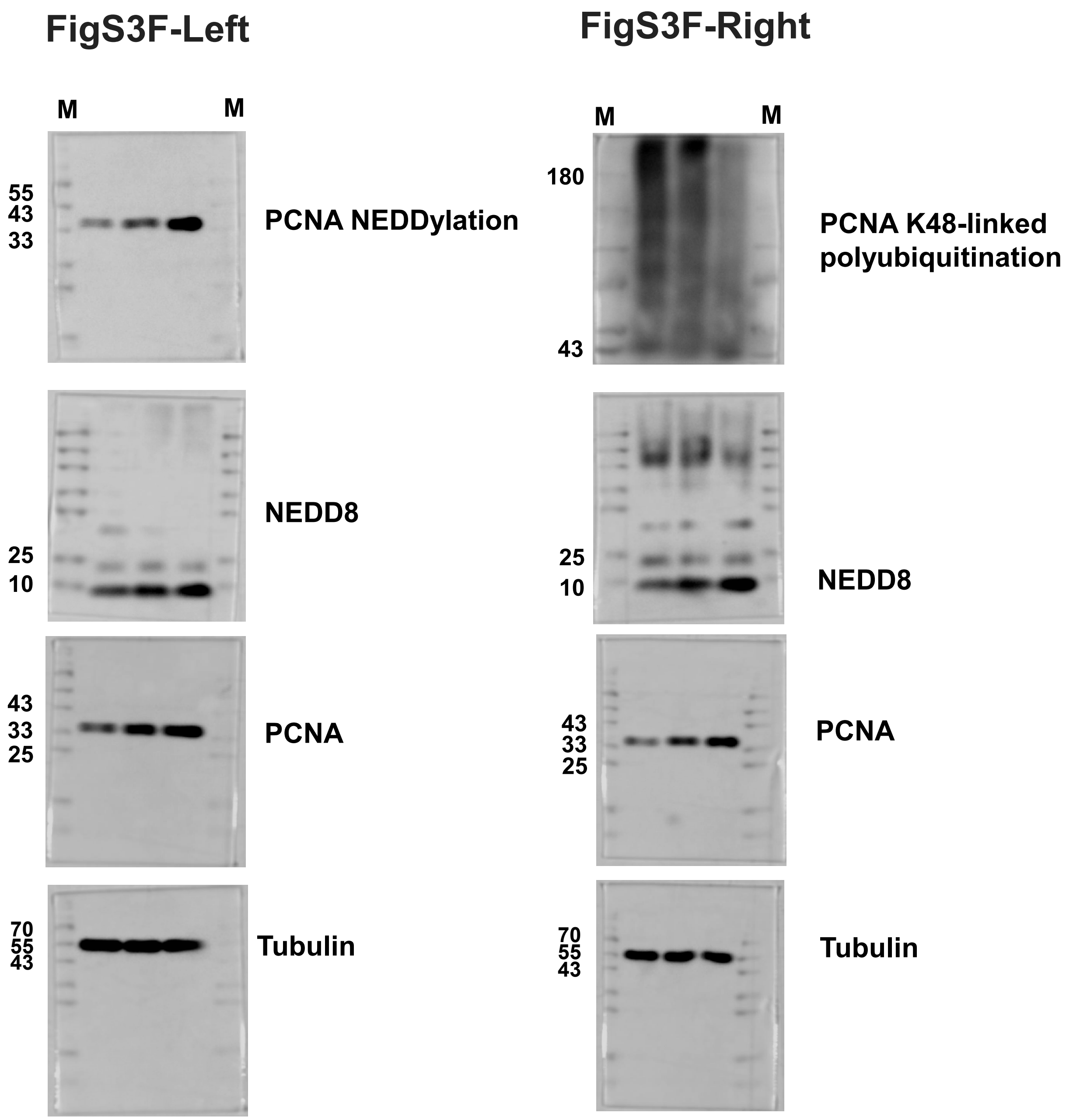

Supplementary Figure 3

FigS3G-Left

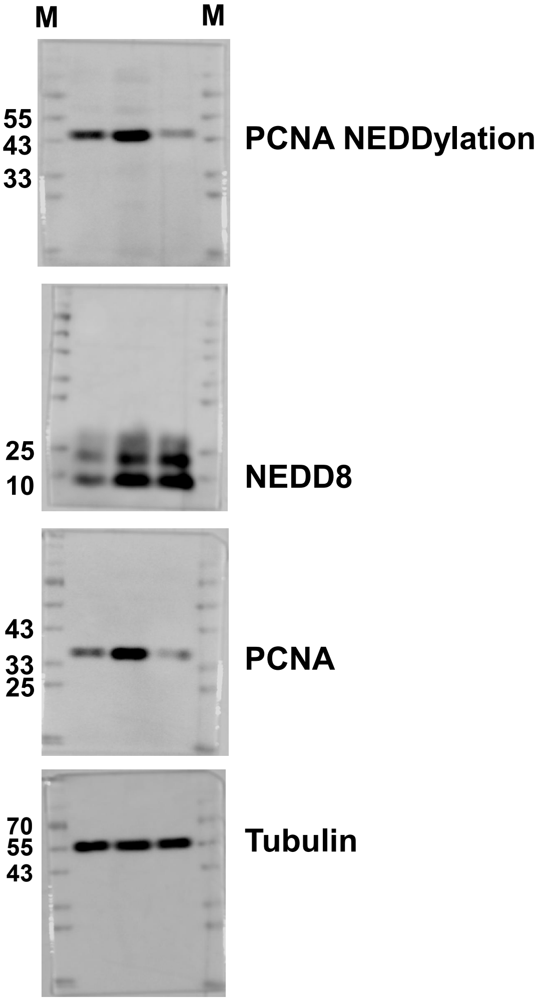

FigS3G-Right

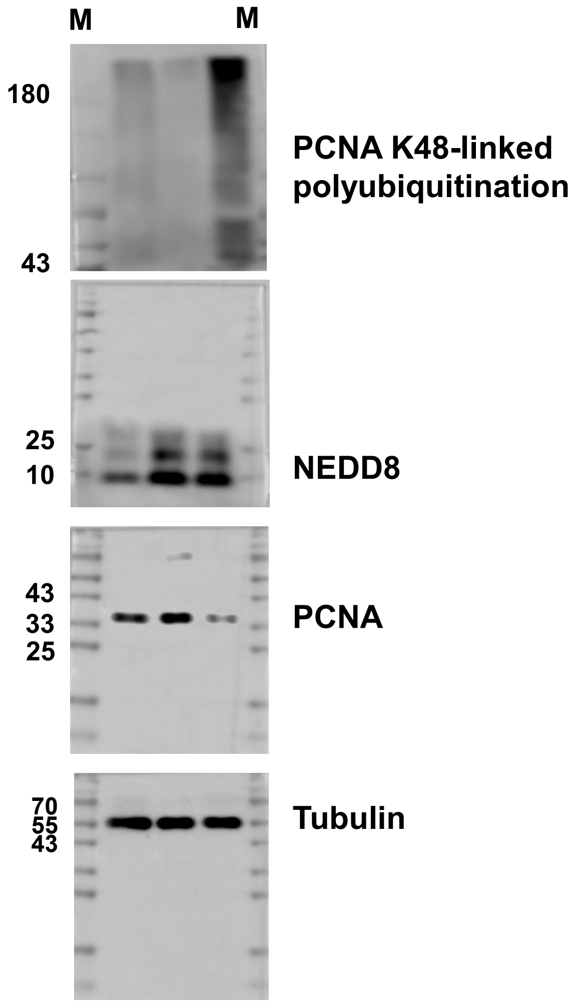

Supplementary Figure 4

FigS4A

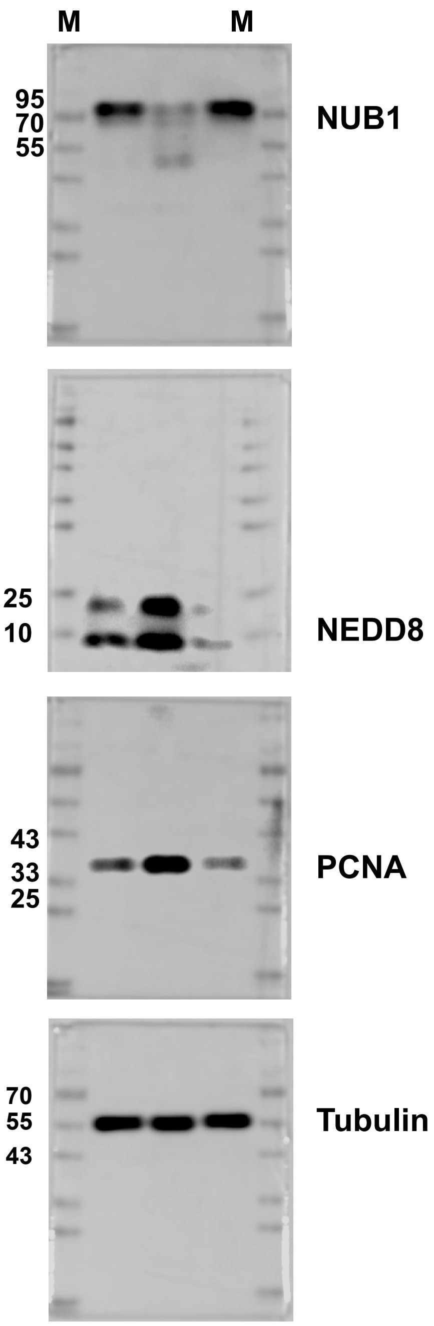

Supplementary Figure 4

FigS4B-Left

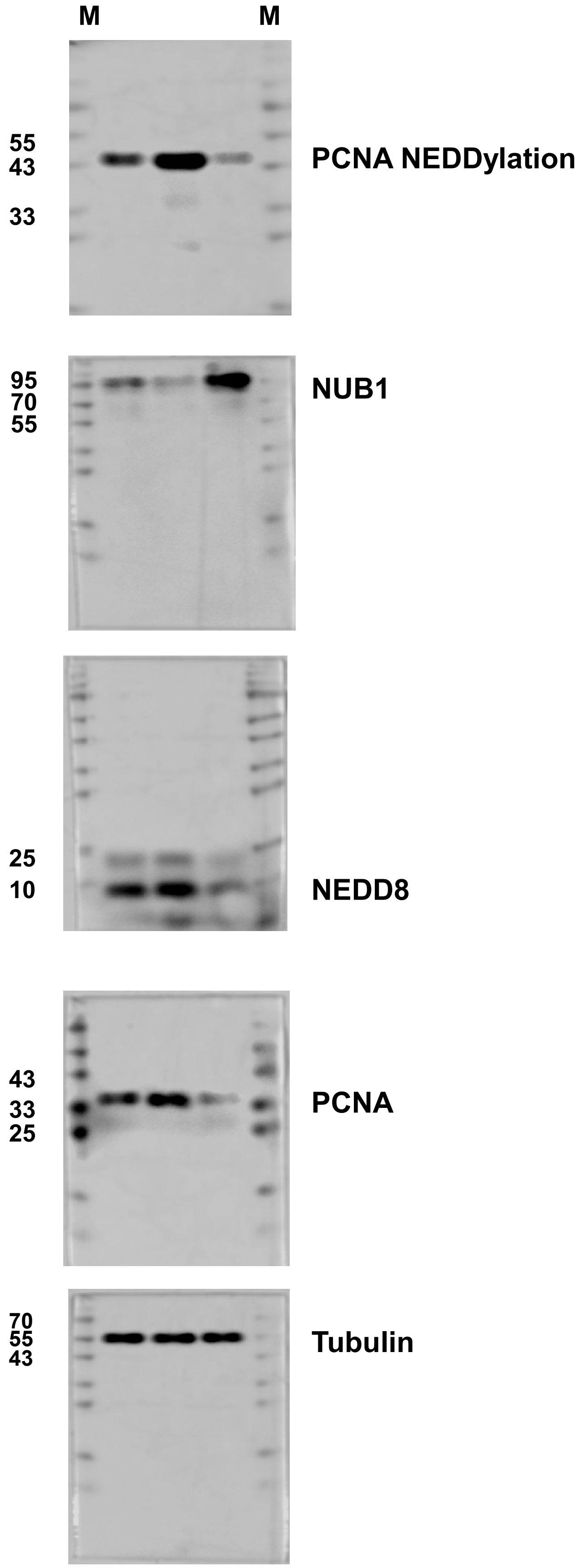

FigS4B-Right

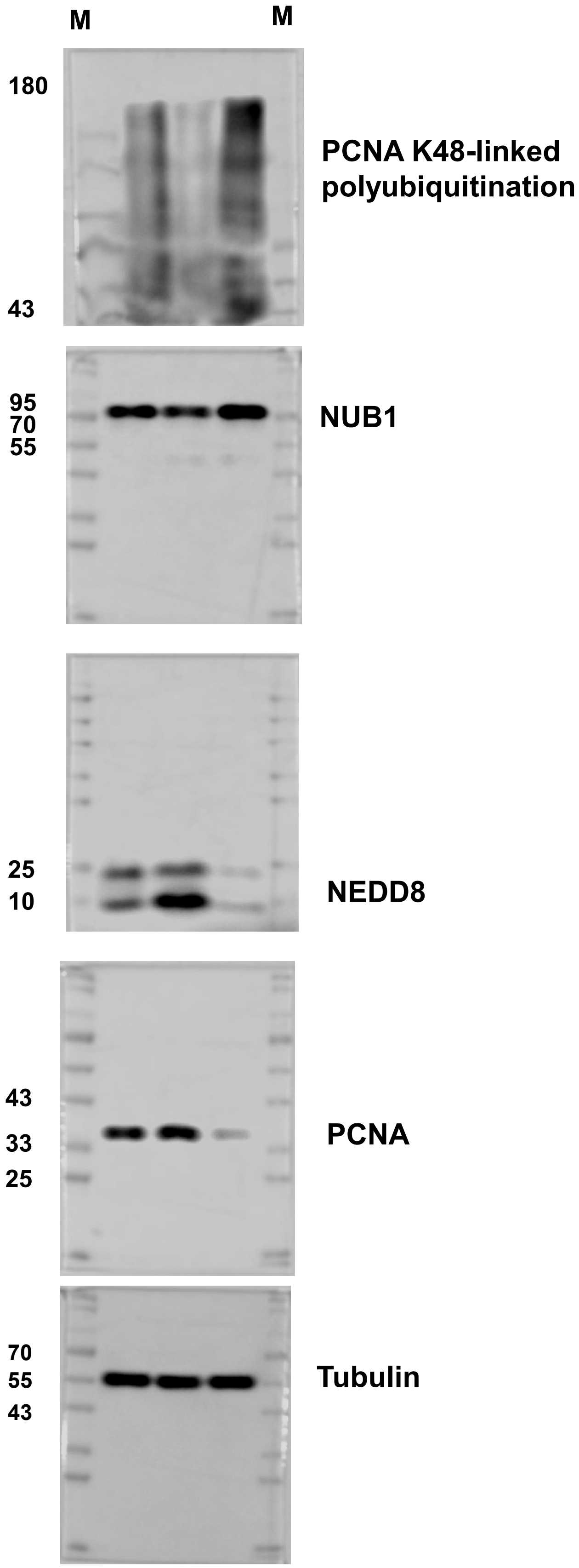

Supplementary Figure 4

FigS4C-Left

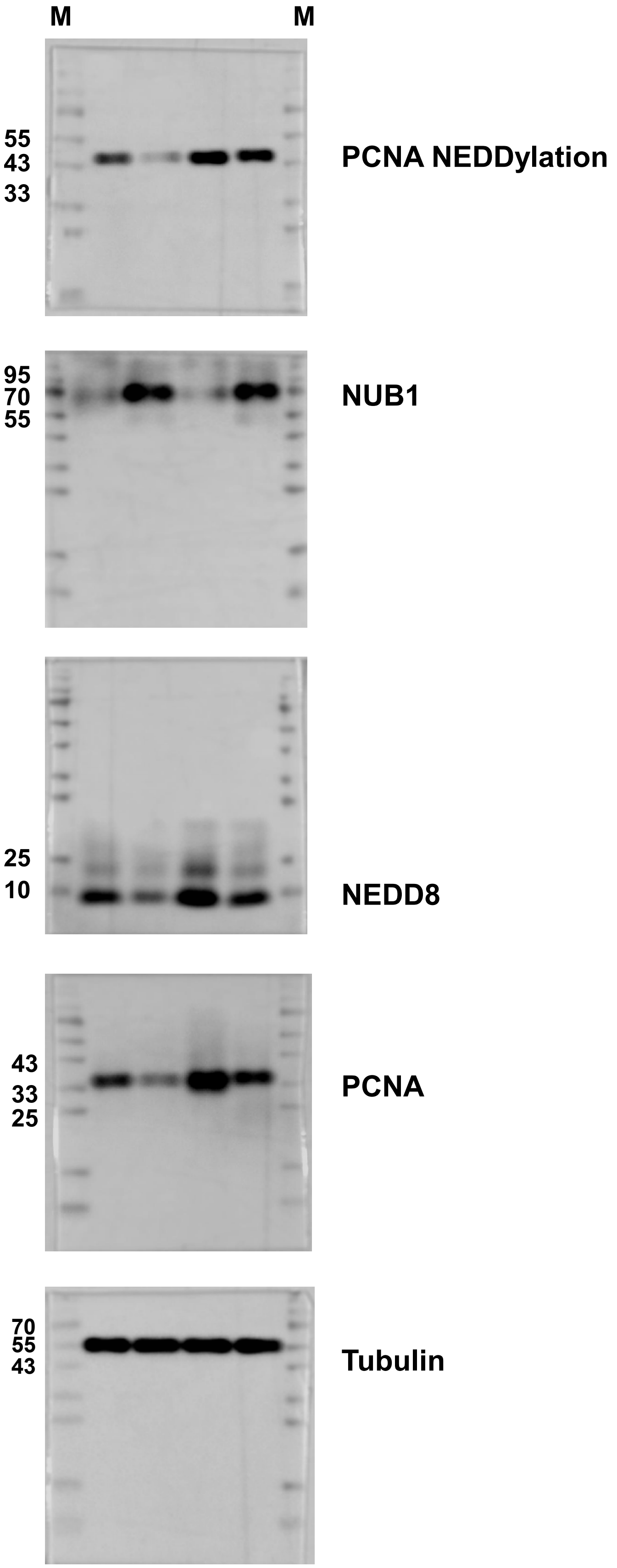

FigS4C-Right

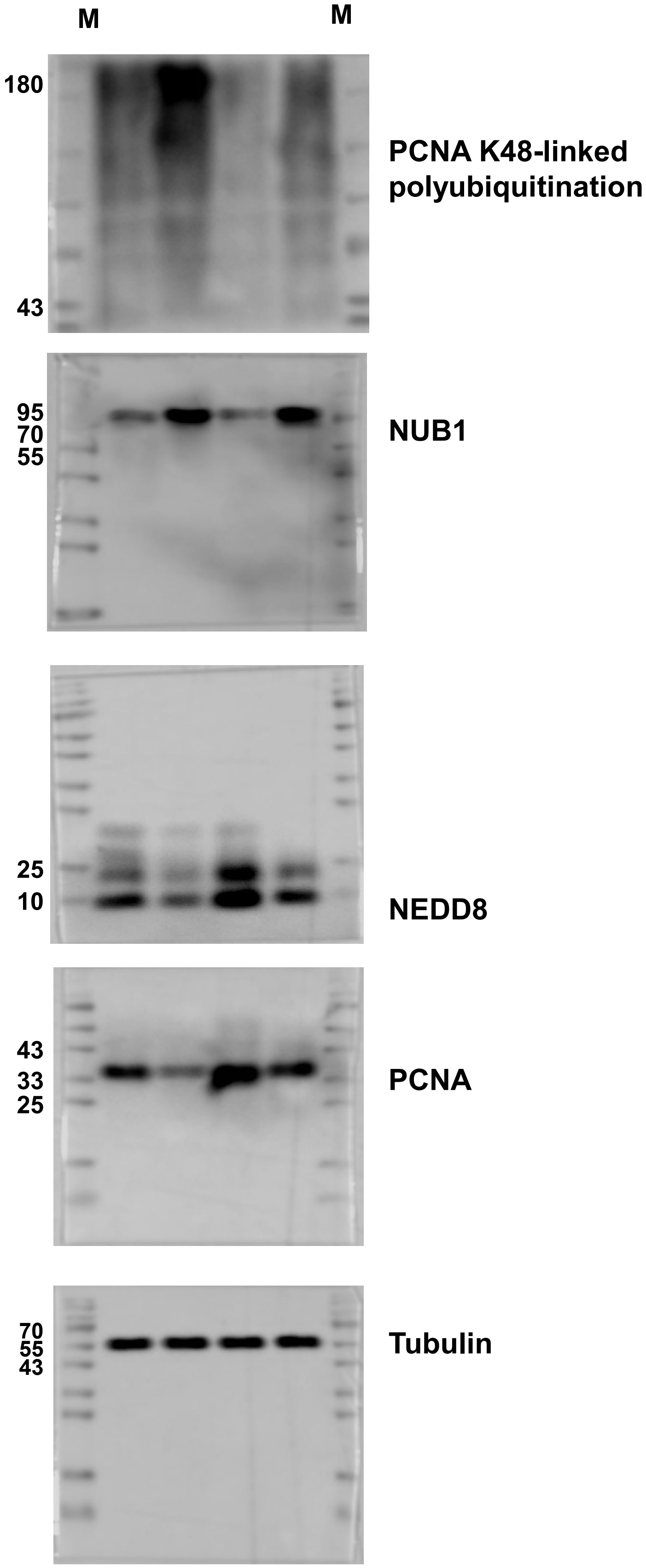

Supplementary Figure 4

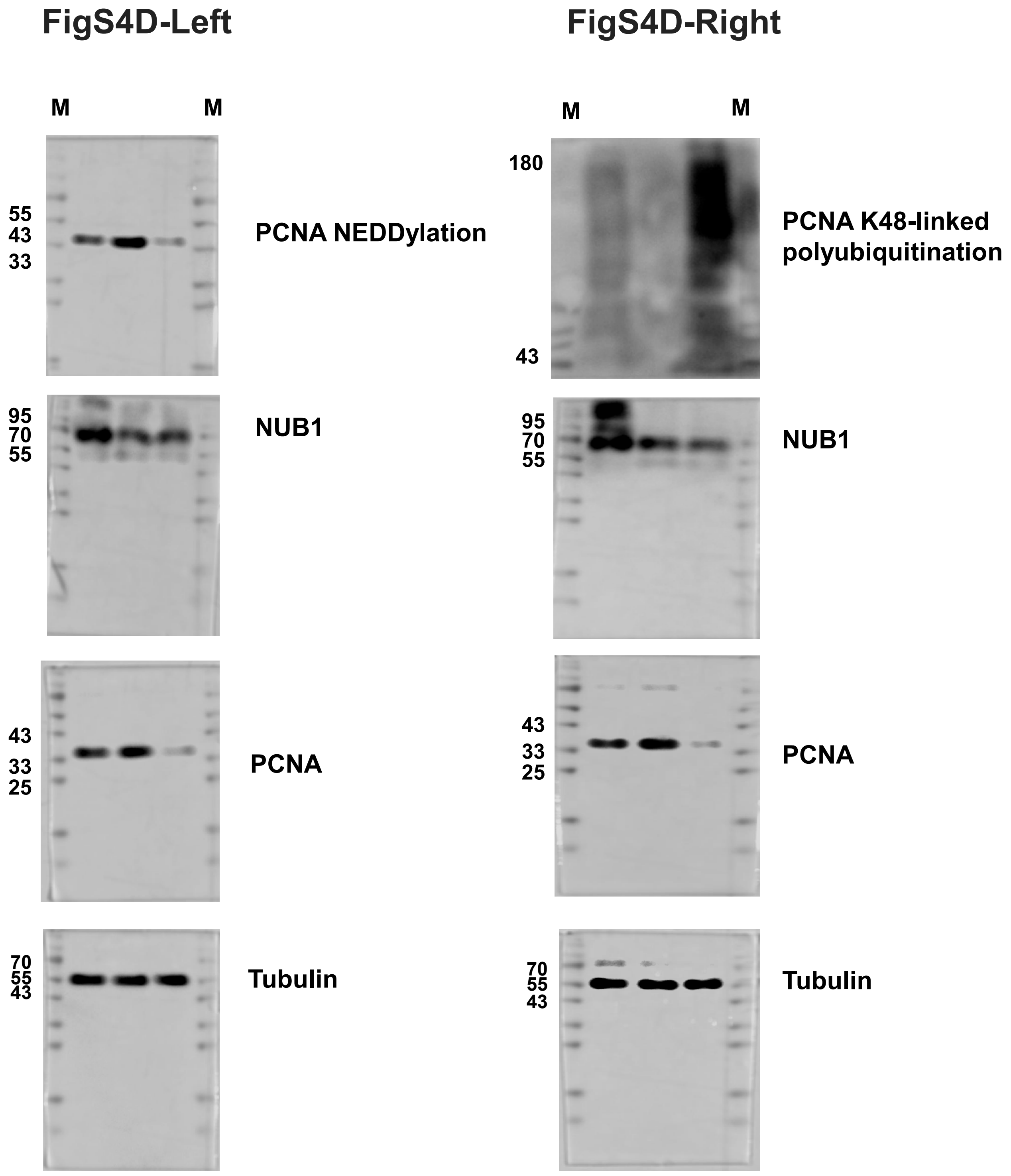

Supplementary Figure 4

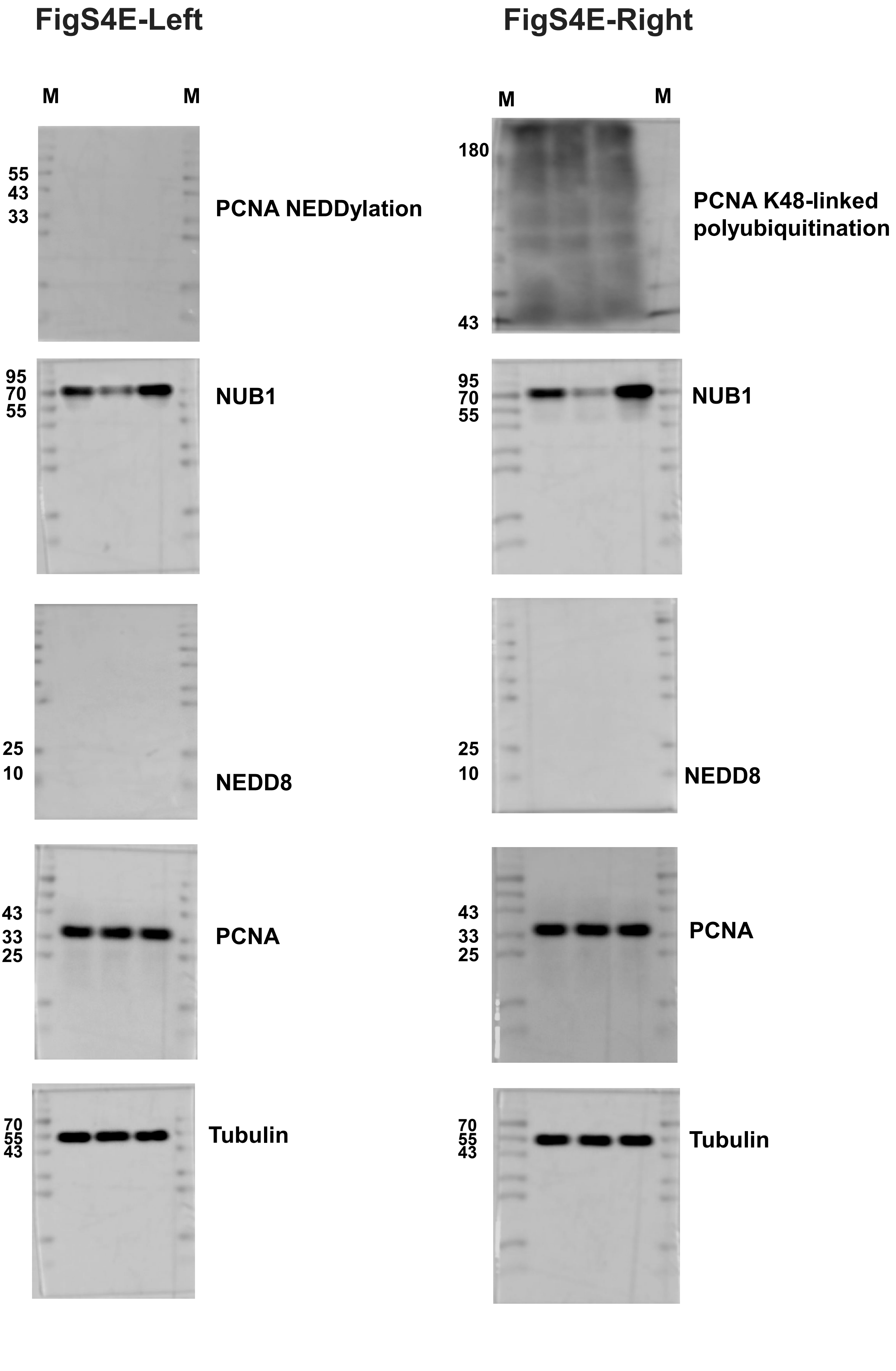

Supplementary Figure 4

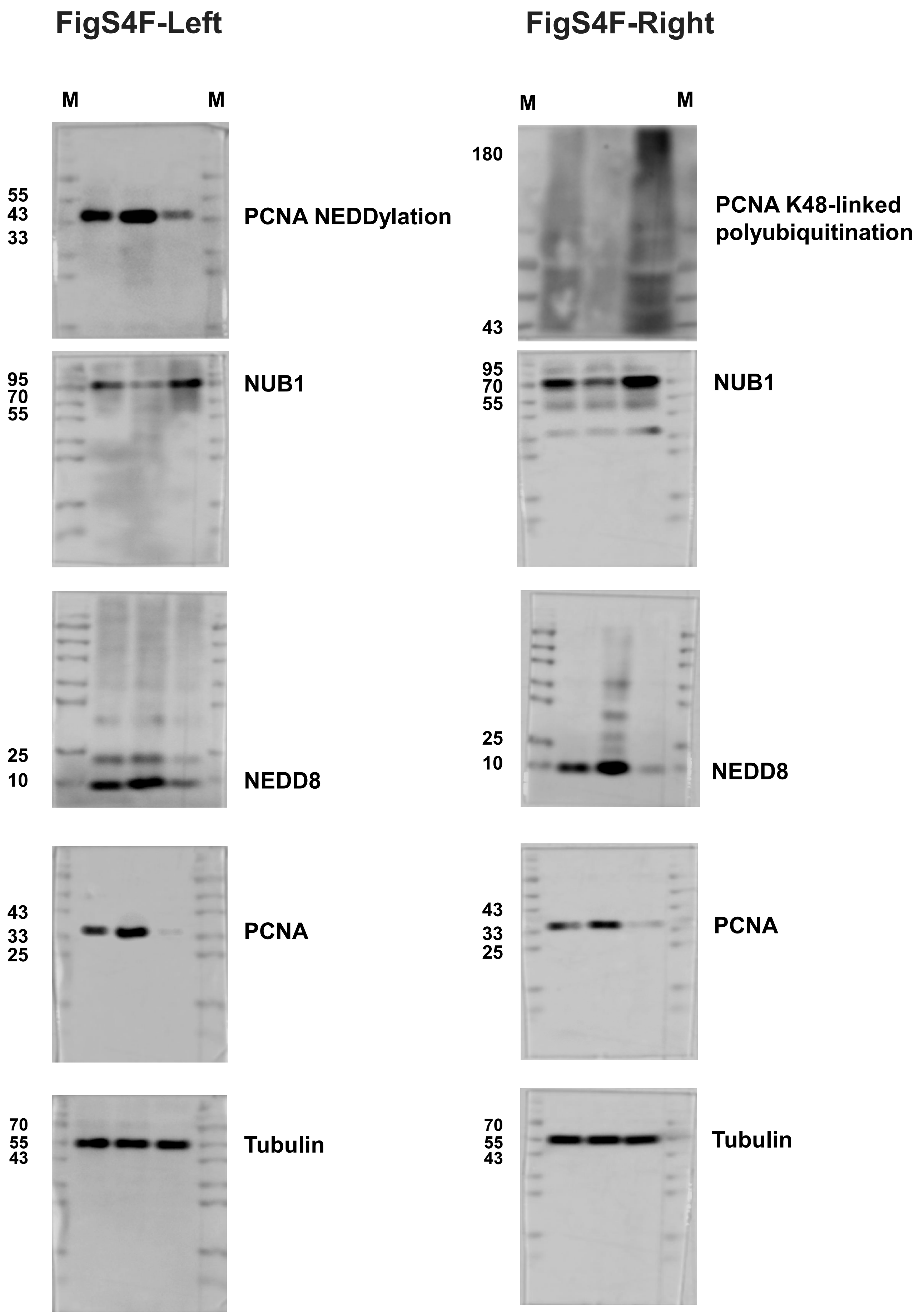

Supplementary Figure 5

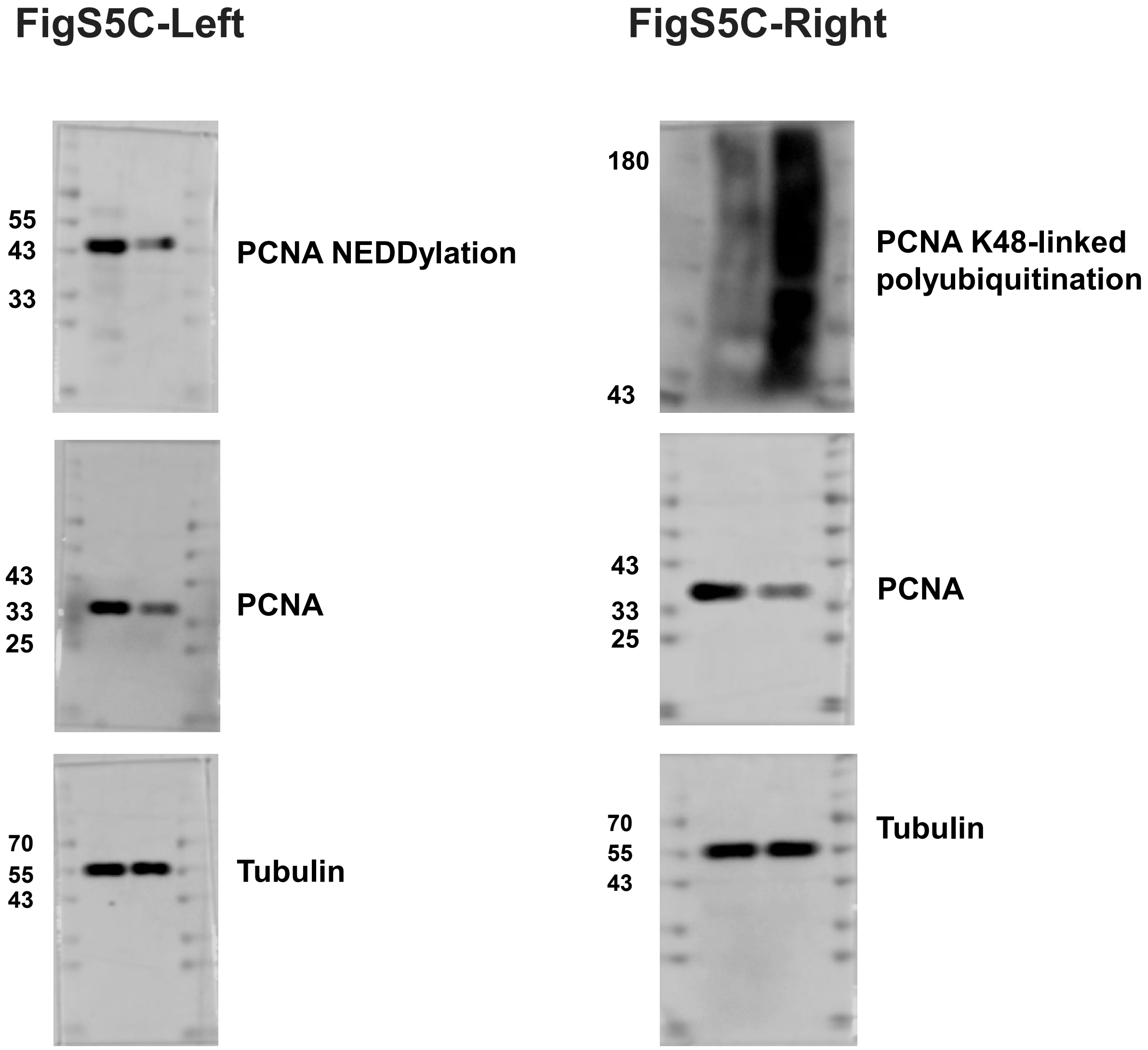

Supplement: Supplementary file 10 — Original western blots [file 41419_2025_7567_MOESM10_ESM.pdf]
